# Artemether-Lumefantrine and Dihydroartemisinin-Piperaquine Exert Inverse Selective Pressure on *Plasmodium Falciparum* Drug Sensitivity-Associated Haplotypes in Uganda

**DOI:** 10.1093/ofid/ofw229

**Published:** 2016-10-25

**Authors:** Aimee R. Taylor, Jennifer A. Flegg, Chris C. Holmes, Philippe J. Guérin, Carol H. Sibley, Melissa D. Conrad, Grant Dorsey, Philip J. Rosenthal

**Affiliations:** 1 WorldWide Antimalarial Resistance Network, University of Oxford, United Kingdom; 2 Department of Statistics, University of Oxford, United Kingdom; 3 Centre for Tropical Medicine and Global Health, Nuffield Department of Clinical Medicine, University of Oxford, United Kingdom; 4 School of Mathematical Sciences and Monash Academy for Cross and Interdisciplinary Mathematical Applications, Monash University, Melbourne, Australia; 5 Department of Genome Sciences, University of Washington, Seattle; 6 Department of Medicine, University of California, San Francisco

**Keywords:** artemether-lumefantrine, dihydroartemisinin-piperaquine, drug resistance, haplotype, Uganda

## Abstract

**Background:**

Altered sensitivity to multiple antimalarial drugs is mediated by polymorphisms in *pfmdr1*, which encodes the *Plasmodium falciparum* multidrug resistance transporter. In Africa the N86Y and D1246Y polymorphisms have been shown to be selected by treatment, with artemether-lumefantrine (AL) and dihydroartemisinin-piperaquine (DP) selecting for wild-type and mutant alleles, respectively. However, there has been little study of *pfmdr1* haplotypes, in part because haplotype analyses are complicated by multiclonal infections.

**Methods:**

We fit a haplotype frequency estimation model, which accounts for multiclonal infections, to the polymorphic *pfmdr1* N86Y, Y184F, and D1246Y alleles in samples from a longitudinal trial comparing AL and DP to treat uncomplicated *P falciparum* malaria in Tororo, Uganda from 2007 to 2012. We regressed estimates onto covariates of trial arm and selective drug pressure.

**Results:**

Yearly trends showed increasing frequency estimates for haplotypes with wild type *pfmdr1* N86 and D1246 alleles and decreasing frequency estimates for haplotypes with the mutant *pfmdr1* 86Y allele. Considering days since prior therapy, we saw evidence suggestive of selection by AL for haplotypes with N86 combined with 184F, D1246, or both, and against all haplotypes with 86Y, and evidence suggestive of selection by DP for 86Y only when

combined with Y184 and 1246Y (haplotype YYY) and against haplotypes NFD and NYY.

**Conclusions:**

Based on our model, AL selected several haplotypes containing N86, whereas DP selection was haplotype specific, demonstrating the importance of haplotype analyses. Inverse selective pressure of AL and DP on the complementary haplotypes NFD and YYY suggests that rotating artemisinin-based antimalarial combination regimens may be the best treatment option to prevent resistance selection.

Uganda has one of the highest estimated burdens of *Plasmodium falciparum* malaria in the world [1]. A mainstay of malaria control is treatment with artemisinin-based combination therapy (ACT) [2]. First-line treatment for uncomplicated malaria in Uganda is artemether-lumefantrine (AL), comprised of artemether, a fast-acting artemisinin derivative, and lumefantrine, a slower acting partner drug. Resistance to artemisinin and its derivatives has been reported in Southeast Asia but, as yet, has not been convincingly proved in Africa [3, 4]. Consistent with these data, efficacies of leading ACTs in Uganda remain high [5, 6]. In older studies, AL generally outperformed artesunate-amodiaquine (AS-AQ), likely due to a high prevalence of resistance to amodiaquine [5]. However, in a recent 3-site trial, recurrent infections were more frequent after treatment with AL than with AS-AQ, suggesting decreased parasite sensitivity to lumefantrine and/or increased sensitivity to amodiaquine associated with changing treatment practices in the country [6].

Monitoring of antimalarial drug efficacy is essential. Genetic markers can be used to monitor sensitivity to some drugs. Definitive lumefantrine resistance is yet to be identified, but single nucleotide polymorphisms in *pfcrt* and *pfmdr1* are associated with decreased sensitivity and are selected in new infections soon after prior therapy. In particular, the wild-type alleles *pfcrt* K76 and *pfmdr1* N86 and D1246 have been associated with decreased ex vivo sensitivity to lumefantrine [7, 8], and an increased prevalence of *pfcrt* K76 and *pfmdr1* N86, 184F, and D1246 has been observed in recurrent infections that emerged after treatment with AL [8–12]. Artesunate-AQ selects in the opposite direction at each allele, consistent with similar selective pressures for the aminoquinolines amodiaquine and chloroquine [13]. An alternative ACT, dihydroartemisinin-piperaquine (DP), has also been highly efficacious for the treatment of malaria in Uganda [14]. The DP partner drug piperaquine is another aminoquinoline with a particularly long half-life (3–4 weeks versus 3–5 days for lumefantrine and 1–3 weeks for the active metabolite of amodiaquine), protecting against recurrent infection [14] and offering monthly chemoprevention [15]. Recent studies in Uganda have shown selection for *pfcrt* and *pfmdr1* alleles in parasites that emerged after treatment with DP, the same selective pressures seen after treatment with AS-AQ and the opposite of selection seen after treatment with AL, although selective pressure appears to be more modest with DP than with AS-AQ [8, 12]. Potential markers of clinically relevant piperaquine resistance are under study [16] after reports of DP treatment failures in Southeast Asia [17].

Although available results provide insight into trends for single polymorphisms, they overlook interactions between alleles and hence may lack power to detect associations if the determinant of a phenotype is not just a single allele but a haplotype, defined here as a sequence of alleles within a gene. We previously characterized *pfmdr1* polymorphisms in samples from malaria episodes in a cohort randomized to treatment with AL or DP, but haplotypes were not characterized because of a high proportion of multiclonal infections that precluded direct haplotype assignment [12]. To better characterize the impacts of the 2 ACTs on *pfmdr1*, we analyzed haplotype frequencies estimated using the previously published *pfmdr1* data [12] and a model that accounts for heteroallelic genotyping outcomes [18].

## METHODS

### Data

In a longitudinal trial conducted in Tororo, Uganda, 312 children, 6 weeks to 12 months of age at enrollment, were randomized to receive either AL or DP for each episode of uncomplicated malaria from 2007 to 2012 [14, 19]. Blood samples were collected before therapy for each episode. A subset of samples was selected for genetic analysis: 50 per trial arm selected randomly from 312 first episodes of malaria, and then 50 per trial arm selected randomly from each 3-month interval, except that only 50 were available for the AL arm and 39 for the DP arm in the final 6 months of the study. Of the 1989 selected samples, 17 were from episodes treated with quinine due to either recent prior treatment failure or severe malaria. Deoxyribonucleic acid was extracted from each selected sample and genotyped at *P falciparum* polymorphisms associated with drug sensitivity, including polymorphisms at codons 86, 184, and 1246 in *pfmdr1*, as previously described [12]. Multiplicity of infection (MOI) was determined based on 10 samples per 3-month interval, as described [12].

### Haplotype Frequency Estimation

The frequencies of haplotypes, defined here by amino acids encoded at *pfmdr1* N86Y, Y184F, and D1246Y (eg, NYD for wild type at each allele), were estimated using a recently described haplotype frequency estimation model [18] (see Supplementary Data), which assumes independence between samples. The model was constructed within a Bayesian framework, requiring prior assignments on the parameters. We used a uniform Dirichlet prior on the haplotype frequencies, and we truncated geometric priors on the MOIs, which were treated as random variables under the model. Based on MOI data [12], the geometric priors were truncated at either 1 (or 2 if the sample was discernibly multiclonal) and 8, with the untruncated mean equal to the average MOI (2.94). The model was fit separately to baseline data (from first episodes) categorized by trial arm (AL and DP), and to post-baseline data categorized by trial arm and year (2008–2012), and by trial arm and days between prior therapy and recurrent infection (4–28, 29–42, 43–56, 57–70, >70 days, based on the distribution of recurrent malaria episodes across both trial arms [12]), henceforth referred to as days since prior therapy.

### Regression

To estimate trends, logit-transformed yearly *pfmdr1* haplotype frequency estimates were regressed onto categorical covariates for trial arm and year, and logit-transformed frequency estimates categorized by days since prior therapy were regressed onto categorical covariates for trial arm and the number of days since prior therapy. We assumed errors were normally distributed with mean zero and fixed variance. An interaction term (between trial arm and either year or days since prior therapy) was included in the matrix of covariates, allowing for different trends between the 2 trial arms. Inference was performed within a Bayesian framework, using Zellner’s g prior [20] to model the joint a priori distribution over the regression parameters. The matrix of covariates was considered nonstochastic. To propagate the uncertainty in the frequency estimates, we used a meta-analytic approach [21]. Trend estimates were calculated from the a posteriori regression parameters. The posterior probability (pp) of a trend increasing or decreasing was based on the proportion of estimates greater or less than zero, respectively (pp = 0.5 indicates a 50% chance of a trend being positive, ie, no trend).

## RESULTS

**Figure 1.**
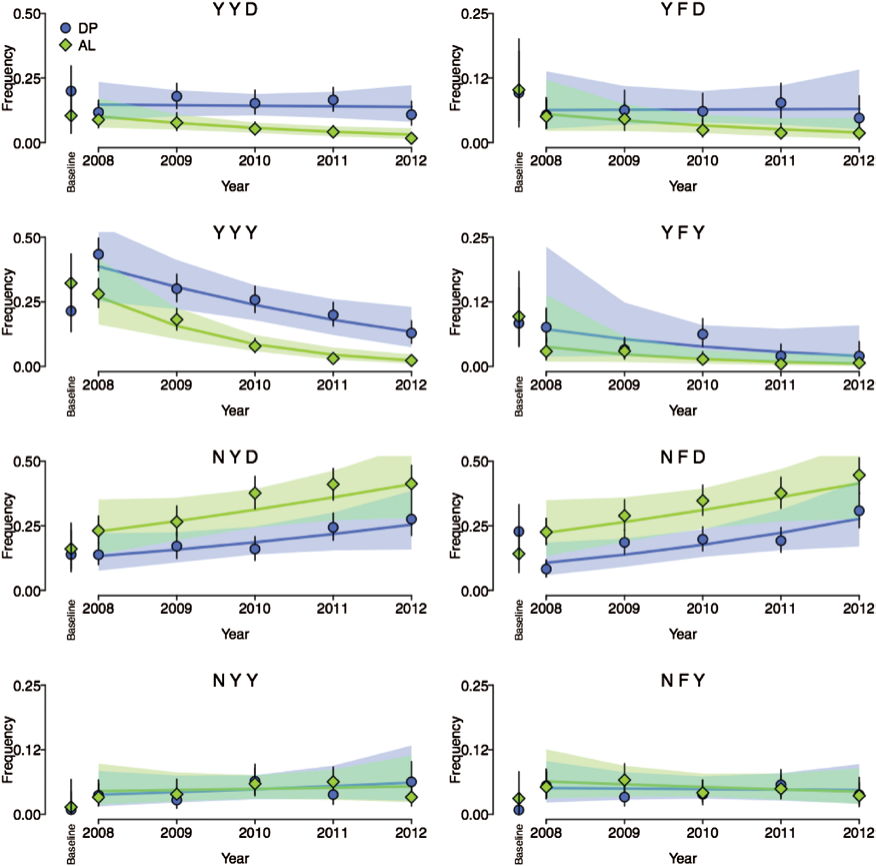
*pfmdr1* haplotype frequency estimates and trends over time. Points and vertical lines denote posterior median haplotype frequency estimates and their 95% credible intervals, respectively, for artemether-lumefantrine and dihydroartemisinin-piperaquine trial arms. Non-vertical lines denote the median trends constructed using a posteriori median estimates of the regression coefficients; shading shows the 95% credible intervals surrounding the median trends. Haplotypes are denoted by their amino acid sequences. Note that the vertical axes for haplotypes YFD, YFY, NYY, and NFY have been scaled to half that of haplotypes YYD, YYY, NYD, and NFD.

**Figure 2.**
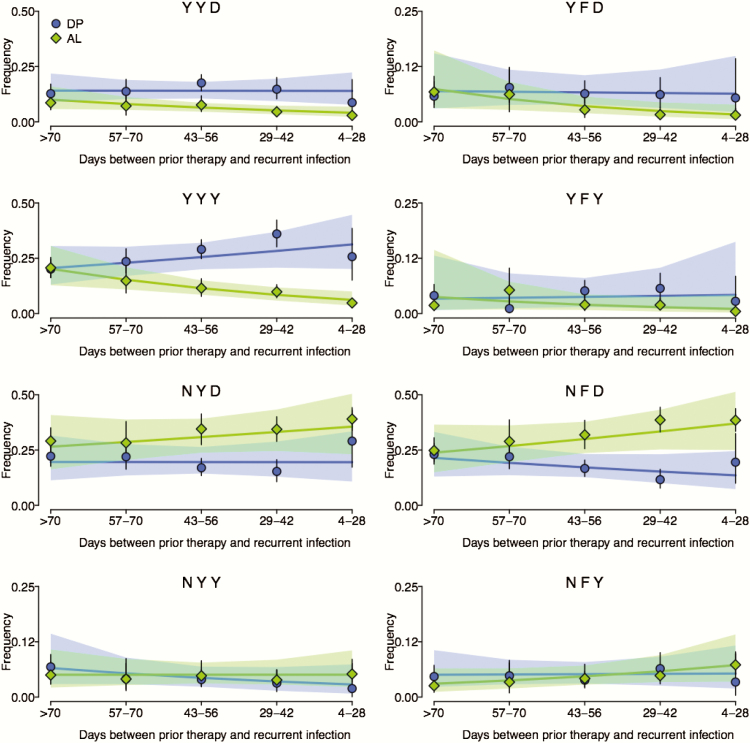
*pfmdr1* haplotype frequency estimates and trends over days since prior therapy. Points, lines, shading, and abbreviations are as defined for Figure 1.

Trial subjects received either AL or DP for each episode of malaria over 5 years, and *P falciparum* polymorphisms were assessed at 3-month intervals. Data were categorized by trial arm, year, and days since prior therapy, and haplotype frequencies were estimated. As expected, haplotypes considering *pfmdr1* N86Y, Y184F, and D1246Y did not differ between trial arms for first episodes (baseline estimates) (Figure 1). Haplotypes YYD, YYY, NYD, and NFD had relatively high baseline frequency estimates, whereas estimates for YFD, YFY, NYY, and NFY were comparatively low, especially the latter 2. Considering haplotypes over time (Figure 1), there were clear differences between trial arms: NYD and NFD predominated in the AL arm, whereas YYD, YYY, YFD, and YFY predominated in the DP arm, highlighting opposite selective pressures on the N86Y and D1246Y alleles. In both trial arms, estimates for haplotypes NYD and NFD increased (pp ≥ 0.95 and pp ≥ 0.96, respectively), whereas estimates for haplotypes YYY and YFY decreased (pp ≥ 0.99 and pp ≥ 0.88, respectively) over time. Estimates for haplotypes YYD and YFD decreased in the AL arm only (pp = 0.99 and 0.92, respectively). Considering the relatively rare haplotypes, NFY estimates decreased slightly in the AL arm (pp = 0.74) and NYY estimates increased slightly in the DP arm (pp = 0.77) over time. Considering days since prior therapy with AL or DP (Figure 2), trends were consistent with AL selection for haplotypes NYD, NFD, and NFY (pp = 0.81, 0.92, and 0.93, respectively), and against YYD, YFD, YYY, and YFY (pp = 0.98, 0.98, 1.00, and 0.87, respectively), and DP selection for YYY (pp = 0.90), and against NFD and NYY (pp = 0.85 and 0.88, respectively). There was no trend associated with selection of NYY by AL (pp = 0.50), or of YYD, YFD, YFY, NYD, and NFY by DP (pp = 0.51**–**0.58). More importantly, results were consistent with opposite selective pressures of AL and DP for the complementary haplotypes NFD and YYY.

## DISCUSSION

We estimated trends in frequency estimates of *pfmdr1* haplotypes associated with antimalarial drug sensitivity using previously published data from a longitudinal trial comparing AL and DP for the treatment of malaria in Tororo, Uganda [12, 14, 19]. Haplotype frequency estimates changed over time in a manner consistent with widespread use of AL to treat malaria in Uganda, and depending on days since prior therapy, in a manner consistent with inverse selective pressures of AL and DP.

Haplotype analyses are important because drug selection may act on a haplotype, rather than a single polymorphism. Haplotype analysis for our samples required a haplotype frequency estimation model to account for the polyclonal nature of most infections in Uganda [18]. The model generates population estimates, requiring separate regressions over time and considering days since prior therapy. Its main advantage is the comprehensive treatment of data from multiclonal infections that preclude haplotype assignment by direct counting. Specifically, each multiclonal infection was modeled as a collection of parasite clones, with MOI equal to the number of clones, allowing use of available MOI data. In practice, each clone represents a population of parasites, but there were insufficient data to model the intrahost parasite populations directly. To accommodate fluctuations in the proportions of clones that share common haplotypes, and to account for uncertainty in the MOI of each sample, MOIs were considered random variables under the model. It is important to note that our model generates frequency estimates, not empirical results. Alternative nonmodel-based haplotype analyses tend to collapse data from multiclonal samples into parsimonious haplotype assignments or to omit multiclonal samples [9, 10, 22, 23]. These analyses may lead to incorrect assignments (as cautioned against in [9]), or biased estimates and data loss [22, 23], but have the advantage of being empirical.

Overall, we observed trends that support and extend previous conclusions based on single polymorphisms in Uganda. Over time, the prevalence of the N86, 184F, and D1246 alleles increased [12], consistent with trends previously identified in Tororo [24], likely due to widespread use of AL to treat malaria across Uganda. The prevalence of the wild-type alleles was greater over time in the AL trial arm compared with the DP arm, consistent with opposite selective pressures seen in samples that emerged soon after prior therapy [12]. Based on our model, which we used to consider haplotypes that emerged under drug pressure, AL selected for haplotypes with N86 only when it was combined with 184F, 1246D, or both (haplotypes NFY, NYD, and NFD), and selected against all haplotypes including 86Y. In contrast, DP selected for 86Y, but only when it was combined with 184Y and 1246Y (haplotype YYY), and selected against the complementary haplotype NFD and also NYY. The fact that the frequency estimates of only 1 haplotype, YYY, increased over days since prior therapy with DP is consistent with studies based on prevalence data, suggesting that the selective pressure of DP on *pfmdr1* is less than that for AS-AQ (in the same direction) or AL (in the opposite direction). This is possibly because, although piperaquine is an aminoquinoline, it is much larger than amodiaquine or chloroquine and thus impacted less by alterations in drug transporters, potentially requiring epistasis between specific combinations of alleles (eg, 86Y, Y184, and 1246Y) for selection on *pfmdr1* to occur. Linkage between the YYY haplotype and another resistance mediator, such as the recently identified copy number variant on chromosome 14 [16], could also cause YYY-specific selection. Indeed, YYY-specific selection may explain why a study in Burkina Faso [25], where the *pfmdr1* 1246Y mutation is uncommon, did not detect selection by DP of *pfmdr1* polymorphisms, contrary to results in Uganda [12]. Considering trends over time (Figure 1), the frequencies of YYY, YYD, YFD, and YFY decreased (the former 2 in the AL arm only), whereas the frequencies of NFD and NYD increased, consistent with selection by AL (Figure 2), and with previous reports that increases in the prevalence and frequency of 184F and D1246 were not as marked as those for N86 [12, 24]. The trends associated with NYY and NFY over time were inconsistent with selection after prior therapy, but they were slight and had relatively low probability, cautioning against over interpretation.

## CONCLUSIONS

Our estimated haplotype frequency trends support previous findings based on single polymorphisms, namely that changes in frequency of particular alleles of *pfmdr1* in Uganda are dominated by the selective pressure of lumefantrine, consistent with increasing use of AL as first-line treatment over the last 10 years [12, 24], and that AL and DP exert inverse selection pressure but to different degrees. The imbalance between AL and DP selective pressure demonstrates the value of considering haplotypes in future analyses of trends. Our results have important implications for informing policies aiming to delay the selection of drug resistance. Consistent with other recent results, they suggest that deploying multiple first-line antimalarial therapies or rotating regimens may best impede the development of resistance to available ACTs [26].

## Supplementary Data

Supplementary materials are available at *Open Forum Infectious Diseases* online. Consisting of data provided by the authors to benefit the reader, the posted materials are not copyedited and are the sole responsibility of the authors, so questions or comments should be addressed to the corresponding author.

The haplotype frequency estimation model is provided in the Supplementary Data. Please email the corresponding author, Aimee R. Taylor, ataylor@hsph.harvard.edu, for further details. For access to the data, please email the alternate corresponding author, Philip J. Rosenthal, philip.rosenthal@ucsf.edu.

## Supplementary Material

ofw229_suppl_supplementary_data
