# Supplementary material for: Artemether-Lumefantrine and Dihydroartemisinin-Piperaquine Exert Inverse Selective Pressure on Plasmodium Falciparum Drug Sensitivity-Associated Haplotypes in Uganda
Source: Open Forum Infect Dis. 2016 Oct 25;4(1):ofw229. doi: 10.1093/ofid/ofw229 (PMC5413987; doi:10.1093/ofid/ofw229)
Supplement: ofw229_suppl_supplementary_data [file ofw229_suppl_supplementary_data.zip › FreqEstimationModel-master/inst/Thesis_methods_chapter.pdf]

Chapter 3

Frequency estimation using prevalence data

3.1 Background

Considerable progress has been made in the fight against malaria in the past 15 years, manifest in a reduction in transmission in many regions [290, 27]. Nevertheless, there is a continued need for genetic surveillance of antimalarial resistance [286], and the changing landscape of transmission renders comparable measures of antimalarial resistance more important than ever. To assess spatiotemporal trends, we require estimates of the frequencies of the key determinants of parasite resistance [95, 96, 276], including alleles at individual SNPs and haplotypes and genotypes spanning multiple SNPs (for example, the quintuple mutant genotype associated with clinical resistance to SP [123]).

In areas of high endemicity people are often infected with multiple parasite clones [225, 258]. Multiclonal infections pose an analytical challenge, since standard analyses of blood sample surveys cannot resolve the clones within multiclonal infections, nor reconstruct the allelic sequences that comprise the haplotypes and genotypes of the constituent clones [276]. Instead, most blood sample surveys generate prevalence data, summaries at the level of the

blood samples [96].

Several statistical models have been designed to overcome the challenge of multiclonal infections and estimate allele, haplotype and genotype frequencies using prevalence data [39, 102, 224, 129, 95, 276, 125, 223]. They are described in detail in chapter 1. With the aim of harnessing the full potential of genetic markers for the surveillance of antimalarial resistance, we present a model that complements and builds upon the existing methods. Several differences set our model apart from existing methods of malaria haplotype and genotype frequency estimation using prevalence data [102, 224, 129, 95, 276, 125]. First, in contrast to all previously published methods, the model makes use of all available data, including those that are incomplete due to unsuccessful genotyping outcomes or study design (see, for example, [83]). Second, in contrast to the Bayesian method by Wigger *et al.* [276] and the model underpinning the freely available online software MalHaploFreq [95], our model is not reliant upon experimentally-derived estimates of sample-wise MOIs. Third, in contrast to most existing approaches [39, 102, 224, 95, 125, 223], it enables rapid analysis of data from three or more SNPs.

Akin to Wigger *et al.* [276], we construct our model within a Bayesian framework. Construction as such provides a straightforward yet comprehensive treatment of uncertainty [88]. We model each infection as an unobserved clonal conglomerate, while taking into account the sample-wise uncertainty in the population-level frequency estimates. Inference within a Bayesian framework also allows the incorporation of specialist knowledge [88], enabling the MOIs to be modelled as random variables whose prior distributions are centred about a reported average.

Also similar to Wigger *et al.* [276], we use a Markov chain Monte Carlo (MCMC) algorithm to sample from the posterior distribution of the haplotype frequencies conditional on the prevalence data. MCMC samplers are of great consequence because they allow sampling from distributions that do not belong to standard families of distributions [90]. Recursive sampling

within the MCMC scheme enables the sampler to efficiently average over the unobserved clonal conglomerates, allowing the analysis of prevalence data for more than three SNPs. Unlike methods designed to generate point estimates, the MCMC method generates a sample set that approximates the full posterior distribution. Posterior summaries, including mean estimates and credible intervals, are readily available given the MCMC sample.

The aim of this chapter is to provide full details of the model and its implementation. For exposition, we focus on results based on simulated data (which also feature in Additional file 2 of [248]). A full demonstration of the model's utility using data from the field can be found in [248] and in chapter 4. In the next section we introduce our notation. In section 3.2.2 we provide a hypothetical example to illustrate the challenge associated with multiclonal infections. We provide full details of the model and its implementation in sections 3.2.3 and 3.2.4, respectively, followed by details of the simulated data (section 3.2.5), convergence (section 3.2.6) and sensitivity analyses (section 3.2.7). Results can be found in section 3.3. The chapter ends with a discussion (section 3.4).

3.2 Methods

3.2.1 Notation

Suppose that I *P. falciparum* positive blood samples, each derived from an independent episode of malaria, are genotyped at J SNPs associated with antimalarial resistance. Due to the multiclonal nature of malaria, when the ith blood sample is genotyped at the jth SNP, the observed datum, y_{ij}, is a summary of all the alleles at the jth SNP belonging to all ≥ 1 clones within the sample. Let $y_{ij} = w$ denote the detection of wild type alleles only, $y_{ij} = m$ denote the detection of mutant type alleles only, $y_{ij} = h$ denote the detection of both wild and mutant type alleles (a *h*eteroallelic SNP) and $y_{ij} =?$ represent a missing genotyping outcome (due to assay failure, for example). For example, $\mathbf{y}_i = (h, ?, w)$ denotes the detection of both wild and mutant

I is the number of blood samples in the dataset (each from a distinct episode of malaria).
J is the number of SNPs genotyped.
$R \leq 2^J$ is the number of haplotypes compatible with the data.
$\boldsymbol{\pi} = (\pi_1, \cdots, \pi_R)$ is a vector of haplotype frequencies. $\boldsymbol{\pi} \in \mathbb{S}^R$, where $\mathbb{S}^R$ denotes the R dimensional simplex, and hence $\sum_{r=1}^{R} \pi_r = 1$.
$\boldsymbol{y} = (\boldsymbol{y}_1, \ldots, \boldsymbol{y}_I)^T$ is the collection of data for the $i = 1, \ldots, I$ blood samples, where $\boldsymbol{y}_i = (y_{i1}, \cdots, y_{iJ})$ is the vector of sample-wise genotyping outcomes for the ith blood sample and y_{ij} is the genotyping outcome of the ith blood sample at the jth SNP, where $y_{ij} \in \{w, m, h, ?\}$ $\forall\, i = 1, \ldots I$ and $j = 1, \ldots J$.
$m_{\max}$ is the global maximum MOI set by the user.
$m_{i\min}$ is the minimum MOI possible for the ith blood sample.
$\boldsymbol{a} = (\boldsymbol{a}_1, \cdots, \boldsymbol{a}_I)^T$ is the collection of unobserved haplotype counts for the $i = 1, \ldots, I$ blood samples, where $\boldsymbol{a}_i = (a_{i1}, \ldots, a_{iR})$ is the vector of haplotype counts for the ith blood sample, and a_{ir} denotes the number of clones in the ith blood sample characterised by the rth haplotype, where $a_{ir} \in \{0, \ldots, m_{\max}\}$ $\forall\, i = 1, \ldots, I$, $r = 1, \ldots R$.
$\boldsymbol{m} = (m_1, \ldots, m_I)$ is the collection of unobserved MOIs for $i = 1, \ldots, I$ blood samples, where the sample-wise MOI, $m_i \in \{1, \cdots, m_{max}\}$ $\forall\, i = 1, \cdots, I$, is the total number of clones in the ith blood sample, $m_i = \sum_{r=1}^{R} a_{ir}$.
$\boldsymbol{H}$ is a $R \times J$ matrix summarising the allelic sequences of the R haplotypes over the J SNPs.
p_{ij} is the proportion of mutant type alleles at the jth SNP in the ith blood sample.
$\boldsymbol{\alpha} = (\alpha_1, \ldots, \alpha_R)$ is the hyperparameter of the Dirichlet prior on the haplotype frequencies, $\boldsymbol{\pi}$.
λ is the hyperparameter for the prior on the MOI, m_i.
ϕ is an additional hyperparameter for the prior on the MOI, m_i.

Table 3.1: Model notation.

type alleles at the first SNP in the ith blood sample, a missing genotype outcome at the second SNP and two or more wild type alleles at the third (since the first SNP is heteroallelic, the ith blood sample must comprise two or more clones). If $J > 1$ (as in the above example), the vector $\boldsymbol{y}_i = (y_{i1}, \ldots, y_{iJ})$ is a summary of the all the haplotypes or genotypes of the clones within the clonal conglomerate. Recall that the term haplotype applies if the SNPs are in the same gene, whereas the term genotype applies if the SNPs belong in multiple genes. Henceforth, haplotypes are referred to exclusively, noting that the same methods apply for genotypes. Let a_{ir} denote the unobserved haplotype count for the rth haplotype (the number of clones characterised by the rth haplotype) in the ith blood sample and $\boldsymbol{a}_i = (a_{i1}, \ldots, a_{iR})$ denote the vector of R haplotype counts for the ith blood sample, where R is the total number of haplotypes compatible across the dataset. Note that $\sum_{r=1}^{R} a_{ir} = m_i$ is the total number of clones in the ith blood sample, henceforth referred to as the MOI. Finally, let $\boldsymbol{\pi} = (\pi_1, \ldots, \pi_R)$ denote the vector of R haplotype frequencies (the proportions of parasite clones in the *P. falciparum* population characterised by haplotypes 1 to R). For reference, a full list of model notation can be found in table 3.1. To illustrate the structure of the data, let us consider the hypothetical example outlined below.

3.2.2 Running example

Suppose parasite DNA extracted from $I = 5$ blood samples is genotyped at $J = 3$ SNPs generating prevalence data (table 3.2) which can be represented by the 5×3 matrix $\boldsymbol{y}$,

Blood sample	SNP 1	SNP 2	SNP 3
1	*w*	*w*	*w*
2	*w*	*m*	*m*
3	*h*	*h*	*h*
4	*w*	*h*	?
5	*w*	*h*	*h*

Table 3.2: A hypothetical prevalence dataset based on five samples genotyped at three SNPs. For a given blood sample and SNP, w denotes the detection of wild type alleles only, m denotes the detection of mutant type alleles only, h denotes the detection of both wild and mutant type alleles and ? indicates the genotyping outcome is missing.

$$\boldsymbol{y} = \begin{array}{c} \\ i=1 \\ \vdots \\ \\ \\ i=5 \end{array} \begin{array}{c} \begin{array}{ccc} j=1 & \dots & j=3 \end{array} \\ \begin{pmatrix} w & w & w \\ w & m & m \\ h & h & h \\ w & h & ? \\ w & h & h \end{pmatrix} \end{array} . \tag{3.1}$$

Since both wild and mutant type alleles are detected at all three SNPs (see equation (3.1)), $R = 2^3 = 8$ haplotypes are compatible with the observed data. The allele sequences of the R

haplotypes are stored in the rows of the $R \times J$ matrix $\boldsymbol{H}$,

$$\boldsymbol{H} = \begin{array}{c} \\ r=1 \\ \vdots \\ \\ \\ \\ \\ \\ r=8 \end{array} \begin{array}{c} \begin{array}{ccc} j=1 & \dots & j=3 \end{array} \\ \begin{pmatrix} 0 & 0 & 0 \\ 1 & 0 & 0 \\ 0 & 1 & 0 \\ 0 & 0 & 1 \\ 1 & 1 & 0 \\ 1 & 0 & 1 \\ 0 & 1 & 1 \\ 1 & 1 & 1 \end{pmatrix} \end{array}, \tag{3.2}$$

where, arbitrarily, '0' denotes a wild type allele and '1' denotes a mutant type allele. Note that under the model, $R \leq 2^J$, because only haplotypes that are possible given the data are considered. Haplotypes that are impossible given the data are assigned zero frequency and do not feature in the $\boldsymbol{H}$ matrix. For example, if the hypothetical data are instead $\boldsymbol{y}_1 = (w, w, h)$ and $\boldsymbol{y}_2 = (m, w, m)$, the matrix of haplotypes would be,

$$\boldsymbol{H} = \begin{array}{c} \\ r=1 \\ \vdots \\ \\ r=4 \end{array} \begin{array}{c} \begin{array}{ccc} j=1 & \dots & j=3 \end{array} \\ \begin{pmatrix} 0 & 0 & 0 \\ 1 & 0 & 0 \\ 0 & 0 & 1 \\ 1 & 0 & 1 \end{pmatrix} \end{array}, \tag{3.3}$$

since the second SNP ($j = 2$) is homoallelic ($y_{12} = y_{22} = w$). Returning to the data in table 3.2 and equation (3.1), suppose the unobserved underlying haplotype count vectors for blood

samples $i = 1, \dots, 5$ are

$$\boldsymbol{a} = \begin{array}{c} i=1 \\ \vdots \\ \\ \\ i=5 \end{array} \overset{\begin{array}{cccccccc} 000_{r=1} & 100 & 010 & 001 & 110 & 101 & 011 & 111_{r=8} \end{array}}{\begin{pmatrix} 1 & 0 & 0 & 0 & 0 & 0 & 0 & 0 \\ 0 & 0 & 0 & 0 & 0 & 0 & 4 & 0 \\ 0 & 1 & 1 & 1 & 0 & 0 & 0 & 0 \\ 0 & 0 & 3 & 1 & 0 & 0 & 1 & 0 \\ 2 & 0 & 0 & 0 & 0 & 0 & 1 & 0 \end{pmatrix}}, \tag{3.4}$$

where a_{ir} denotes the number of clones with the rth haplotype in the ith sample. For reference, the allele sequences of haplotypes $r = 1, \dots, R$ are shown as column headings. For example, $\boldsymbol{a}_5 = (2,0,0,0,0,0,1,0)$ indicates that the 5th blood sample contains three clones, two with haplotype '000' and one with haplotype '011', such that when the first SNP is genotyped pure wild type alleles are detected and when the second and third SNPs are genotyped both wild and mutant type alleles are detected, giving rise to $\boldsymbol{y}_5 = (w, h, h)$, as seen in equation (3.1). Given $\boldsymbol{a}$, the empirical sample haplotype frequencies are directly calculable,

$$\begin{aligned} \boldsymbol{\pi} &= (\pi_1, \dots, \pi_R) \equiv (\pi_{000}, \pi_{100}, \pi_{010}, \pi_{001}, \pi_{110}, \pi_{101}, \pi_{011}, \pi_{111}), \\ &= \frac{\sum_{i=1}^{I} \boldsymbol{a}_i}{\sum_{i=1}^{I} \sum_{r=1}^{R} a_{ir}}, \\ &= \frac{\sum_{i=1}^{I} \boldsymbol{a}_i}{\sum_{i=1}^{I} m_i}, \\ &= \left(\frac{3}{16}, \frac{1}{16}, \frac{4}{16}, \frac{2}{16}, \frac{0}{16}, \frac{0}{16}, \frac{6}{16}, \frac{0}{16} \right). \end{aligned}$$

In this hypothetical example, the vector of haplotype frequencies is trivial to estimate because the sample haplotype counts are known. In reality, the haplotype counts are not observed. To estimate the vector of haplotype frequencies, the following model is proposed.

3.2.3 The model

We propose the following model (figure 3.1) to estimate the vector of haplotype frequencies, $\boldsymbol{\pi}$, conditional on prevalence data. A number of simplifying assumptions are made in its construction:

1. blood samples are independently distributed;
2. clones are independently distributed (for example, the probability of being infected with two clones with allelic sequences '000' and '011' is $\pi_{000} \times \pi_{011}$);
3. perfect detection (for example, if a person is infected with ten clones, nine of which are characterised by '000' and one by '100', the mutant allele is detected);
4. alleles are error-free (for example '0' is correctly identified as '0' and not as '1').

The terms in which these assumptions are introduced are indicated below; their implications are discussed in section 3.4. Latent variables include the MOIs, m_i for $i = 1, \ldots, I$, and the haplotype count vectors, $\boldsymbol{a}_i$ for $i = 1, \ldots I$. The prevalence data for the ith blood sample, y_{ij} for $j = 1, \ldots, J$, are modelled directly upon the unobserved haplotype count vector, $\boldsymbol{a}_i$. Since the model is constructed within a Bayesian framework, we put prior distributions on $\boldsymbol{\pi}$, $\boldsymbol{a}_i$ and m_i for $i = 1, \ldots I$. The priors are specified according to the dependencies in figure 3.1. The joint posterior density is,

$$\begin{aligned} \rho\left(\boldsymbol{\pi}, \boldsymbol{a}, \boldsymbol{m} \mid \boldsymbol{y}\right) &= \frac{\rho\left(\boldsymbol{y}|\boldsymbol{a}\right)\rho\left(\boldsymbol{a}|\boldsymbol{m}, \boldsymbol{\pi}\right)\rho\left(\boldsymbol{m}\right)\rho\left(\boldsymbol{\pi}\right)}{\rho(\boldsymbol{y})} \\ &\propto \prod_{i=1}^{I}\left\{\prod_{j=1}^{J}\left\{\rho\left(y_{ij}|\boldsymbol{a}_i\right)\right\}\rho\left(\boldsymbol{a}_i|m_i, \boldsymbol{\pi}\right)\rho\left(m_i\right)\right\}\rho\left(\boldsymbol{\pi}\right), \end{aligned} \tag{3.5}$$

where the product over $i = 1, \ldots, I$ results from the assumptions of independence between blood samples, and the product over $j = 1, \ldots, J$ results from an assumption of conditional independence between SNPs within the ith blood sample given the haplotype counts, $\boldsymbol{a}_i$. The

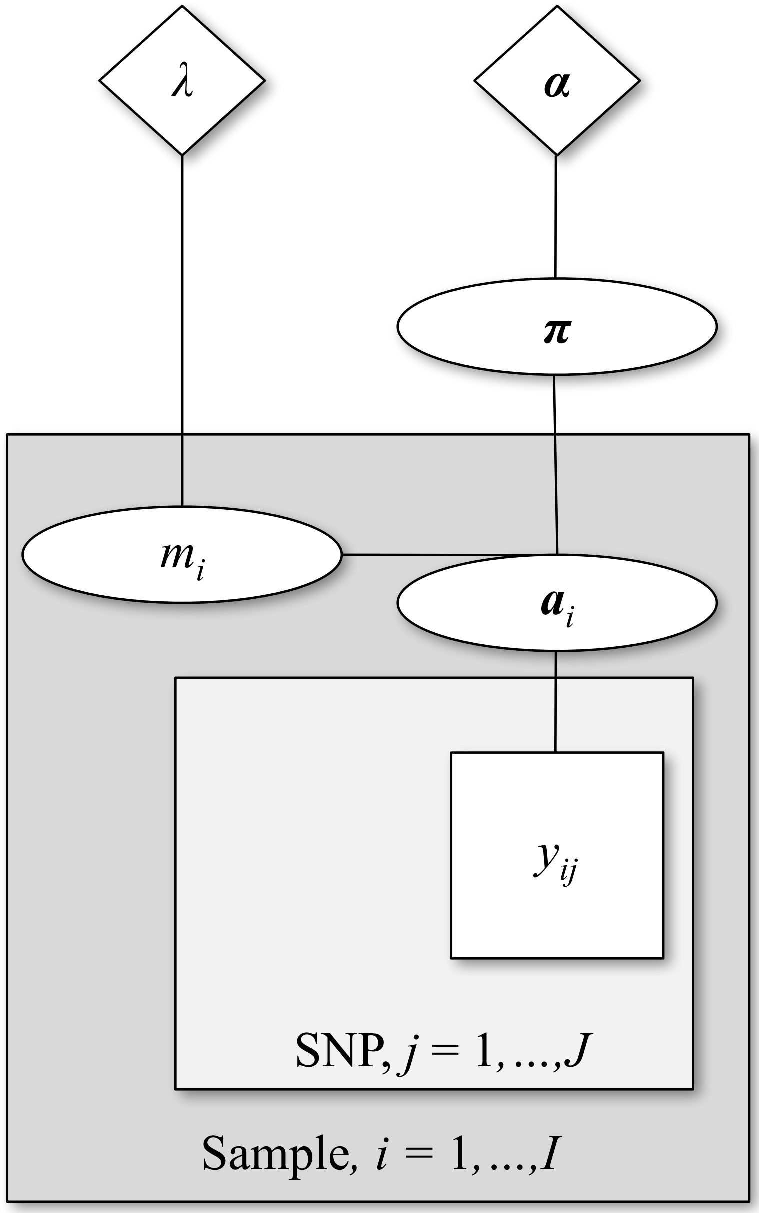

Figure 3.1: Haplotype frequency estimation model for prevalence data. The graph shows the model quantities and their conditional dependencies. The data y_{ij} for $I = 1,\ldots,I$ and $j = 1\ldots,J$ are represented by a square. Ellipses denote unobserved variables: the vectors of haplotype counts, $\boldsymbol{a}_i$ for $I = 1,\ldots,I$; the MOI, m_i for $I = 1,\ldots,I$; and the haplotype frequency vector, $\boldsymbol{\pi}$. The diamonds represent the hyperparameters of the priors on m_i and $\boldsymbol{\pi}$ (λ and $\boldsymbol{\alpha}$, respectively). The density of the joint distribution is $\rho\left(\boldsymbol{\pi},\boldsymbol{a},\boldsymbol{m},\boldsymbol{y}\right) = \rho\left(\boldsymbol{y} \mid \boldsymbol{a}\right)\rho\left(\boldsymbol{a} \mid \boldsymbol{m},\boldsymbol{\pi}\right)\rho\left(\boldsymbol{m}\right)\rho\left(\boldsymbol{\pi}\right)$.

likelihood, $\rho\left(y_{ij}|\boldsymbol{a}_i\right)$, is specified as follows,

$$\rho\left(y_{ij}|\boldsymbol{a}_i\right) = \begin{cases} 1 \text{ if } y_{ij} = w \text{ and } p_{ij} = 0, \\ 1 \text{ if } y_{ij} = m \text{ and } p_{ij} = 1, \\ 1 \text{ if } y_{ij} = h \text{ and } 0 < p_{ij} < 1, \\ 1 \text{ if } y_{ij} \text{ is missing}, \\ 0 \text{ otherwise}, \end{cases} \tag{3.6}$$

where p_{ij} is the proportion of mutant type alleles at the jth SNP in the ith blood sample. That is, the number of haplotypes with a mutant type allele at the jth SNP, $\boldsymbol{a}_i \cdot \boldsymbol{h}_j$, where $\boldsymbol{h}_j$ is the jth column vector of the matrix $\boldsymbol{H}$, enlisting all the allele states (mutant '1' or wild type '0') of the R possible haplotypes at the jth SNP, normalised by the total number of haplotypes in the ith blood sample ($m_i = \sum_{r=1}^{R} a_{ir}$),

$$p_{ij} = \frac{\boldsymbol{a}_i \cdot \boldsymbol{h}_j}{\sum_{r=1}^{R} a_{ir}}. \tag{3.7}$$

Note that in equation (3.7) above, 100% detectability of the minority allele is assumed. For example, if the ith blood sample is infected with ten clones, nine with haplotype '000' and one with haplotype '100', $p_{i1} > 0$. That is, the clone with haplotype '100' is detected despite constituting only 10% of the total haplotype count for the ith blood sample. The correct identification of each allele is also assumed. For example, the alleles of both haplotypes at the second and third SNPs are identified correctly as '0' not '1', hence $p_{i2} = p_{i3} = 0$. As mentioned above, we discuss the implications of these assumptions in section 3.4.

The priors on $\boldsymbol{a}_i$ and $\boldsymbol{\pi}$ are specified as follows,

$$\rho\left(\boldsymbol{a}_i|m_i, \boldsymbol{\pi}\right) = \mathscr{M}\text{ultinomial}(\boldsymbol{a}_i \mid m_i, \boldsymbol{\pi}), \tag{3.8}$$

which implies independence between clones, and

$$\rho\,(\boldsymbol{\pi}) = \mathscr{D}\text{irichlet}(\boldsymbol{\pi} \mid \boldsymbol{\alpha}), \tag{3.9}$$

where $\boldsymbol{\alpha} = (\alpha_1, \ldots, \alpha_R)$ is the hyperparameter of the prior on $\boldsymbol{\pi}$. We set $\alpha_r = 1$ for $r = 1, \ldots, R$ with the effect that all R haplotypes are regarded *a priori* as biologically feasible and equally probable. Doing so provides an objective basis against which the validity of the results can be compared. Alternatively, one can incorporate prior knowledge about the viability of the different haplotypes by varying the elements of $\boldsymbol{\alpha}$. Instead of specifying one definitive prior for the MOI, we propose four options (equations (3.10) to (3.13)), with a view to selecting the one that provides the best fit to the data. All four are probability distributions (which assume independence between clones) on the set of integers $\{m_{i\min}, \ldots, m_{\max}\}$, where $m_{i\min} = 2$ if the ith blood sample is discernibly multiclonal (if for some $j \in \{1, \ldots, J\}$, $y_{ij} = h$) and 1 otherwise, while $m_{\max}$ is a global maximum set by the user and based on auxiliary data where available. All four follow classic distributions,

1. $$\rho(m_i) = \mathscr{U}\text{niform}(m_i \mid m_{i\min}, m_{\max}); \tag{3.10}$$

2. $$\begin{aligned}\rho(m_i) &= \mathscr{P}\text{oisson}_{\text{truncated}}(m_i \mid \lambda, m_{i\min}, m_{\max}),\\ &= \frac{\mathscr{P}\text{oisson}(m_i \mid \lambda)}{\left(\sum_{m_i=m_{i\min}}^{m_{\max}} \mathscr{P}\text{oisson}(m_i \mid \lambda)\right)};\end{aligned} \tag{3.11}$$

3. $$\begin{aligned}\rho(m_i) &= \mathscr{G}\text{eometric}_{\text{truncated}}(m_i \mid \lambda, m_{i\min}, m_{\max}),\\ &= \frac{\mathscr{G}\text{eomtric}(m_i \mid \lambda)}{\left(\sum_{m_i=m_{i\min}}^{m_{\max}} \mathscr{G}\text{eometric}(m_i \mid \lambda)\right)};\end{aligned} \tag{3.12}$$

4. $$\begin{aligned}\rho(m_i) &= \mathscr{N}\text{egative } \mathscr{B}\text{inomial}_{\text{truncated}}(m_i \mid \lambda, \phi, m_{i\min}, m_{\max}),\\ &= \frac{\mathscr{N}\text{egative } \mathscr{B}\text{inomial}(m_i \mid \lambda, \phi)}{\left(\sum_{m_i=m_{i\min}}^{m_{\max}} \mathscr{N}\text{egative } \mathscr{B}\text{inomial}(m_i \mid \lambda, \phi)\right)};\end{aligned} \tag{3.13}$$

where λ denotes the mean of the Poisson, geometric and negative binomial distributions and can be interpreted as an approximate *a priori* average MOI; and ϕ denotes the dispersion

parameter of the negative binomial distribution whose density is parameterised as follows,

$$\mathcal{N}\text{egative }\mathcal{B}\text{inomial}(m_i \mid \lambda, \phi) = \frac{\Gamma(m_i + \phi)}{\Gamma(\phi)\, m_i!} \left(\frac{\phi}{\lambda + \phi}\right)^{\phi} \left(1 - \frac{\phi}{\lambda + \phi}\right)^{m_i} \tag{3.14}$$

The parameter λ is set by the user and based on auxiliary data where available. In this chapter, the Poisson prior is used for the sensitivity analyses based on simulated data. Upon analysing real data, the prior providing the best fit is selected using posterior predictive checks (see section A.2 for example).

3.2.4 The sampler

One cannot evaluate the posterior density given by equation (3.5) directly: it does not belong to a standard family of distributions and evaluating the normalising constant, $\rho(\boldsymbol{y})$, requires integrating over all possible $\boldsymbol{a}_i$, m_i and $\boldsymbol{\pi}$. Nevertheless, it is a distribution whose density can be evaluated pointwise up to a normalising constant. We therefore use a MCMC algorithm to sample from the distribution whose density is given by equation (3.5). More specifically, we use a Gibbs sampler. The Gibbs sampler works by breaking the problem down into blocks of variables which are iteratively sampled. For a given block, the variables within it are sampled from their full conditional distribution given the data and all variables outside the block at their current values. The variables under our model are $\boldsymbol{\pi}$, $\boldsymbol{a}_i$ and $m_i \;\; \forall\, i = 1, \ldots, I$; the target density is $\rho(\boldsymbol{\pi}, \boldsymbol{a}, \boldsymbol{m} \mid \boldsymbol{y})$ (equation (3.5)); and the blocks are

1. $\boldsymbol{a}_i$ and m_i given $\boldsymbol{\pi}$ and $\boldsymbol{y}$ for each $i = 1, \ldots, I$ independently,
2. $\boldsymbol{\pi}$ given $\boldsymbol{a}, \boldsymbol{m}$ and $\boldsymbol{y}$.

In other words, on each iteration of the sampler, for each $i = 1, \ldots, I$, we update the MOI and haplotype count vector, $\boldsymbol{a}_i$ and m_i, conditional on the current estimate of the haplotype frequency vector, $\boldsymbol{\pi}$, and the data, $\boldsymbol{y}$. Second, we update the haplotype frequency vector, $\boldsymbol{\pi}$,

given the collection of all the haplotype count vectors, $\boldsymbol{a}$, the collection of all the MOIs, $\boldsymbol{m}$, and the data, $\boldsymbol{y}$. The full conditional distribution of the variables in the first block, $\boldsymbol{a}_i$ and m_i, does not belong to a standard family of distributions, hence we use a Metropolis-Hastings step to update $\boldsymbol{a}_i$ and m_i. Due to conjugacy, the full conditional distribution of $\boldsymbol{\pi}$ is a Dirichlet distribution whose parameter vector is based on the haplotype count vectors. Hence we can Gibbs sample haplotype frequency vectors exactly. The mathematical details of the updates within both blocks are outlined in detail below. Starting at iteration $t = 0$, initial estimates, $\boldsymbol{\pi}^{(t)}$, $\boldsymbol{m}^{(t)}$ and $\boldsymbol{a}^{(t)}$, are either drawn from their respective priors (see section 3.2.3) or set equal to some specified values. For $t > 0$, the sampler proceeds as follows.

Update MOI and haplotype counts

The density of the joint conditional distribution of $\boldsymbol{a}_i$ and m_i is given by

$$\rho\left(\boldsymbol{a}_i, m_i \mid \boldsymbol{\pi}^{(t-1)}, \boldsymbol{y}\right) \propto \prod_{j=1}^{J} \left\{\rho\left(y_{ij} \mid \boldsymbol{a}_i\right)\right\} \rho\left(\boldsymbol{a}_i \mid m_i, \boldsymbol{\pi}^{(t-1)}\right) \rho\left(m_i\right) \ \forall\, i = 1, \ldots, I, \tag{3.15}$$

where $\rho\left(y_{ij} \mid \boldsymbol{a}_i\right)$ and $\rho\left(\boldsymbol{a}_i \mid m_i, \boldsymbol{\pi}^{(t-1)}\right)$ are given by equations (3.6) and (3.8), respectively, and $\rho\left(m_i\right)$ depends on the choice of MOI prior (equations (3.10) to (3.13)). Regardless of the MOI prior choice, the joint conditional distribution (equation (3.15)) does not belong to a standard family of distributions, hence cannot be sampled directly. Instead we use a Metropolis-Hastings update to sample from the joint conditional distribution whose density is given by equation (3.15). The Metropolis-Hastings step relies on the availability of a proposal distribution whose density can be evaluated. To ensure the MCMC algorithm only explores space compatible with the observed data, we use a proposal, q, conditioned upon the current vector of haplotype counts and the current MOI,

$$\left(\boldsymbol{a}_i^{\star}, m_i^{\star}\right) \sim q\left(\cdot \mid \boldsymbol{a}_i^{(t-1)}, m_i^{(t-1)}\right). \tag{3.16}$$

The joint proposal (equation (3.16)) is broken down into two stages, $m_i^\star \sim q_m\left(\cdot \mid \boldsymbol{a}_i^{(t-1)}, m_i^{(t-1)}\right)$ and $\boldsymbol{a}_i^\star \sim q_a\left(\cdot \mid m_i^\star, \boldsymbol{a}_i^{(t-1)}, m_i^{(t-1)}\right)$, described in detail below.

Propose a new MOI: The proposal q_m is implemented as follows. For, $i = 1,\ldots,I$, $m_i^\star$ is generated by either adding or subtracting a clone to the existing MOI,

$$m_i^\star = \begin{cases} m_i^{(t-1)} \pm 1 \text{ with probability } = 1/2 & \text{if } m_{\text{masked}i}^{(t-1)} > 0 \text{ and } m_i^{(t-1)} < m_{\max}, \\ m_i^{(t-1)} + 1 \text{ with probability } = 1 & \text{if } m_{\text{masked}i}^{(t-1)} = 0, \\ m_i^{(t-1)} - 1 \text{ with probability } = 1 & \text{if } {m_i}^{(t-1)} = m_{\max}, \end{cases} \tag{3.17}$$

where

$$m_{\text{masked}i}^{(t-1)} = \sum_{r=1}^{R} a_{\text{masked}ir}^{(t-1)} \text{ and} \tag{3.18}$$

$$\boldsymbol{a}_{\text{masked}i}^{(t-1)} = f(\boldsymbol{a}_i^{(t-1)} \mid \boldsymbol{y}_i). \tag{3.19}$$

The function, $f : \boldsymbol{a}_i^{(t-1)} \rightarrow \boldsymbol{a}_{\text{masked}_i}^{(t-1)}$ conditional on $\boldsymbol{y}_i$, ensures that the proposed MOI, $m_i^\star$, is compatible with $\boldsymbol{y}_i$. Essentially, $\boldsymbol{a}_{\text{masked}_i}^{(t-1)}$ is a template of $\boldsymbol{a}_i^{(t-1)}$, but with all counts whose removal would render $\boldsymbol{a}_i^{(t-1)}$ incompatible with $\boldsymbol{y}_i$ set equal to zero, thus 'masked', preventing their removal. The counts whose removal would render $\boldsymbol{a}_i^{(t-1)}$ incompatible with $\boldsymbol{y}_i$ include those that contribute either a solitary mutant allele or a solitary wild type allele. The function f is determined algorithmically as follows.

> First assign $\boldsymbol{a}_{\text{masked}_i}^{(t-1)} \leftarrow \boldsymbol{a}_i^{(t-1)}$. Second, if $\boldsymbol{a}_i^{(t-1)} \cdot \mathbf{h}_j = 1$, locate the solitary mutant count (r for which $a_{ir}^{(t-1)} \times h_{rj} = 1$) and, if $y_{ij} \neq ?$, set $a_{\text{masked}_{ir}}^{(t-1)} \leftarrow 0$ (see footnote[1]). Third, if $\boldsymbol{a}_i^{(t-1)} \cdot \mathbf{h}_j = \left(\sum_{r=1}^{R} a_{ir}^{(t-1)}\right) - 1$, locate the solitary wild type count (r for which $a_{ir}^{(t-1)} > 0$ and $a_{ir}^{(t-1)} \times h_{rj} = 0$) and, if $y_{ij} \neq ?$, set

[1] As an aside, in section 6.2.4 we encounter an application where $m_{i\min} = 2$ for all $i = 1,\ldots,I$. In this case, if $m_i^{(t-1)} = 2$, $a_{\text{masked}_{ir}}^{(t-1)} \leftarrow 0$ for all r corresponding to $a_{ir}^{(t-1)} > 0$.

$$a_{\text{masked}_{ir}}^{(t-1)} \leftarrow 0.$$

For example, if the hypothetical $\boldsymbol{a}$ given by equation (3.4) were our estimate at iteration $t-1$, $\boldsymbol{a}_{\text{masked}}^{(t-1)}$ would be given by

$$\boldsymbol{a}_{\text{masked}}^{(t-1)} = \begin{array}{c} \\ i=1 \\ \vdots \\ \\ \\ i=5 \end{array} \begin{array}{c} \begin{array}{cccccccc} 000_{r=1} & 100 & 010 & 001 & 110 & 101 & 011 & 111_{r=8} \end{array} \\ \begin{pmatrix} \mathbf{0} & 0 & 0 & 0 & 0 & 0 & 0 & 0 \\ 0 & 0 & 0 & 0 & 0 & 0 & 4 & 0 \\ 0 & \mathbf{0} & \mathbf{0} & \mathbf{0} & 0 & 0 & 0 & 0 \\ 0 & 0 & 3 & \mathbf{0} & 0 & 0 & 1 & 0 \\ 2 & 0 & 0 & 0 & 0 & 0 & \mathbf{0} & 0 \end{pmatrix} \end{array}, \tag{3.20}$$

where the elements that have been 'masked' are highlighted in bold, leading to,

$$\boldsymbol{m}_{\text{masked}}^{(t-1)} = \sum_{r=1}^{R} \boldsymbol{a}_{\text{masked}r}^{(t-1)} = \begin{array}{c} i=1 \\ \vdots \\ \\ \\ i=5 \end{array} \begin{pmatrix} 0 \\ 4 \\ 0 \\ 4 \\ 2 \end{pmatrix}. \tag{3.21}$$

For any $m_i^{\star}$, $m_i^{(t-1)}$ and $\boldsymbol{a}_i^{(t-1)}$, we can calculate $q_m\left(m_i^{\star} \mid m_i^{(t-1)}, \boldsymbol{a}_i^{(t-1)}\right)$, which is equal to $1/2$ or 1 as described by equation (3.17) and equation (3.22) below,

$$q_m\left(m_i^{\star} \mid m_i^{(t-1)}, \boldsymbol{a}_i^{(t-1)}\right) = \begin{cases} 1/2 & \text{if } m_{\text{masked}i}^{(t-1)} > 0 \text{ and } m_i^{(t-1)} < m_{\max}, \\ 1 & \text{if } m_{\text{masked}i}^{(t-1)} = 0, \\ 1 & \text{if } m_i^{(t-1)} = m_{\max}. \end{cases} \tag{3.22}$$

The probability density of the reverse step, $q_m\left(m_i^{(t-1)} \mid m_i^\star, \boldsymbol{a}_i^\star\right)$, is given by

$$q_m\left(m_i^{(t-1)} \mid m_i^\star, \boldsymbol{a}_i^\star\right) = \begin{cases} 1/2 & \text{if } m^\star_{\text{masked}i} > 0 \text{ and } m_i^\star < m_{\max}, \\ 1 & \text{if } m^\star_{\text{masked}i} = 0, \\ 1 & \text{if } m_i{}^\star = m_{\max}, \end{cases} \tag{3.23}$$

where $m^\star_{\text{masked}i}$ is derived from $\boldsymbol{a}_i^\star$ following equations (3.17) and (3.19). Note that the probability density of the forward step does not necessarily equal the probability of the reverse step, see for example figure 3.2, where $q_m\left(m_i^\star \mid m_i^{(t-1)}, \boldsymbol{a}_i^{(t-1)}\right) = 1$, but $q_m\left(m_i^{(t-1)} \mid m_i^\star, \boldsymbol{a}_i^\star\right) = 1/2$.

Propose a new haplotype count The proposal q_a is implemented as follows. For $i = 1, \dots I$, the newly proposed haplotype count vector, $\boldsymbol{a}_i^\star$, is generated by either adding or subtracting a haplotype count vector representing a single clone, $\boldsymbol{a}_{\text{single clone}}$, to or from the current haplotype count vector, $\boldsymbol{a}_i^{(t-1)}$, conditional upon $m_i^\star$:

$$\boldsymbol{a}_i^\star = \begin{cases} \boldsymbol{a}_i^{(t-1)} - \boldsymbol{a}_{\text{single clone}}, \text{ where } \boldsymbol{a}_{\text{single clone}} \sim \mathscr{M}\text{ultinomial}\left(1, \boldsymbol{p}_{\text{sub}i}{}^{(t-1)}\right) \text{ if } m_i^\star = m_i^{(t-1)} - 1, \\ \boldsymbol{a}_i^{(t-1)} + \boldsymbol{a}_{\text{single clone}}, \text{ where } \boldsymbol{a}_{\text{single clone}} \sim \mathscr{M}\text{ultinomial}\left(1, \boldsymbol{p}_{\text{add}i}\right) \text{ if } m_i^\star = m_i^{(t-1)} + 1. \end{cases} \tag{3.24}$$

The probability vectors $\boldsymbol{p}_{\text{sub}i}{}^{(t-1)}$ and $\boldsymbol{p}_{\text{add}i}$ are calculated as follows,

$$\boldsymbol{p}_{\text{sub}i}{}^{(t-1)} = \frac{\boldsymbol{a}^{(t-1)}_{\text{masked}i}}{\sum_{r=1}^R a^{(t-1)}_{\text{masked}ir}} \text{ and } \boldsymbol{p}_{\text{add}i} = \frac{\boldsymbol{a}_{\text{compatible}_i}}{\sum_{r=1}^R a_{\text{compatible}_{ir}}}, \tag{3.25}$$

where $\boldsymbol{a}^{(t-1)}_{\text{masked}i}$ is given by equation (3.19) above. The vector $\boldsymbol{a}_{\text{compatible}_i}$ is the ith row of the look up matrix $\boldsymbol{a}_{\text{compatible}}$ in which the compatibilities of the $r = 1, \dots, R$ haplotypes with $\boldsymbol{y}_i$ are

recorded. For example, for the hypothetical dataset (equation (3.1)),

$$\boldsymbol{a}_{\text{compatible}} = \begin{array}{c} \\ \boldsymbol{y}_1 = (w,w,w) \\ \boldsymbol{y}_2 = (w,m,m) \\ \boldsymbol{y}_3 = (h,h,h) \\ \boldsymbol{y}_4 = (w,h,?) \\ \boldsymbol{y}_5 = (w,h,h) \end{array} \begin{array}{c} \begin{array}{cccccccc} 000_{r=1} & 100 & 010 & 001 & 110 & 101 & 011 & 111_{r=8} \end{array} \\ \left(\begin{array}{cccccccc} 1 & 0 & 0 & 0 & 0 & 0 & 0 & 0 \\ 0 & 0 & 0 & 0 & 0 & 0 & 1 & 0 \\ 1 & 1 & 1 & 1 & 1 & 1 & 1 & 1 \\ 1 & 0 & 1 & 1 & 0 & 0 & 1 & 0 \\ 1 & 0 & 1 & 1 & 0 & 0 & 1 & 0 \end{array}\right) \end{array} \tag{3.26}$$

where '1' denotes compatible and '0' denotes incompatible, and compatibility is defined as follows. If only wild or mutant type alleles are detected at the jth SNP of the ith sample ($y_{ij} = w$ or $y_{ij} = m$, respectively) only allele sequences with wild or mutant type alleles at the jth SNP are compatible, respectively; whereas if both wild and mutant type alleles are detected at the jth SNP of the ith sample, or if the datum is missing ($y_{ij} = h$ or $y_{ij} = ?$), allele sequences with both wild and mutant types alleles at the jth SNP are compatible. Akin to the dependence of $\boldsymbol{p}_{\text{sub}_i}^{(t-1)}$ upon $\boldsymbol{a}_{\text{masked}_i}^{(t-1)}$, the dependence of $\boldsymbol{p}_{\text{add}_i}$ upon $\boldsymbol{a}_{\text{compatible}_i}$ ensures the compatibility of $\boldsymbol{a}_i^\star$ with $\boldsymbol{y}_i$.

Note that given any $\boldsymbol{a}_i^\star$, $m_i^\star$, $\boldsymbol{a}_i^{(t-1)}$ and $m_i^{(t-1)}$, we can compute $q_a\left(\boldsymbol{a}_i^\star \mid m_i^\star, \boldsymbol{a}_i^{(t-1)}, m_i^{(t-1)}\right)$ following the multinomial distribution described above (equation (3.24)). That is to say,

$$q_a(\boldsymbol{a}_i^\star \mid m_i^\star, \boldsymbol{a}_i^{(t-1)}, m_i^{(t-1)}) = \begin{cases} p_{\text{add}_{ir}} \text{ if } m_i^\star = m_i^{(t-1)} + 1, \\ p_{\text{sub}_{ir}}^{(t-1)} \text{ if } m_i^\star = m_i^{(t-1)} - 1, \end{cases} \tag{3.27}$$

where the r specifies the rth element corresponding to $a_{\text{single clone}_r} = 1$ (the only element of $\boldsymbol{a}_{\text{single clone}}$ not equal to zero). The probability density of the reverse step, $q_a\left(\boldsymbol{a}_i^{(t-1)} \mid m_i^{(t-1)}, \boldsymbol{a}_i^\star, m_i^\star\right)$,

is also governed by equation (3.24), leading to

$$q_a(\boldsymbol{a}_i^{(t-1)} \mid m_i^{(t-1)}, \boldsymbol{a}_i^\star, m_i^\star) = \begin{cases} p_{\text{add}ir} \text{ if } m_i^{(t-1)} = m_i^\star + 1, \\ p^\star_{\text{sub}ir} \text{ if } m_i^{(t-1)} = m_i^\star - 1. \end{cases} \tag{3.28}$$

Acceptance probability: Having generated $m_i^\star$ and $\boldsymbol{a}_i^\star$, the newly proposed parameters are either rejected, in which case $\left(m_i^{(t)}, \boldsymbol{a}_i^{(t)}\right) \leftarrow \left(m_i^{(t-1)}, \boldsymbol{a}_i^{(t-1)}\right)$, or accepted with probability,

$$\mathbb{P}\left(\left(\boldsymbol{a}_i^{(t)}, m_i^{(t)}\right) \leftarrow (\boldsymbol{a}_i^\star, m_i^\star)\right) = \min\left\{1, \frac{\rho\left(\boldsymbol{a}_i^\star, m_i^\star \mid \boldsymbol{\pi}, \boldsymbol{y}_i\right)}{\rho\left(\boldsymbol{a}_i^{(t-1)}, m_i^{(t-1)} \mid \boldsymbol{\pi}, \boldsymbol{y}_i\right)} \frac{q\left(\boldsymbol{a}_i^{(t-1)}, m_i^{(t-1)} \mid \boldsymbol{a}_i^\star, m_i^\star\right)}{q\left(\boldsymbol{a}_i^\star, m_i^\star \mid \boldsymbol{a}_i^{(t-1)}, m_i^{(t-1)}\right)}\right\}, \tag{3.29}$$

where

$$\frac{\rho\left(\boldsymbol{a}_i^\star, m_i^\star \mid \boldsymbol{\pi}, \boldsymbol{y}_i\right)}{\rho\left(\boldsymbol{a}_i^{(t-1)}, m_i^{(t-1)} \mid \boldsymbol{\pi}, \boldsymbol{y}_i\right)} = \frac{\rho\left(\boldsymbol{a}_i^\star \mid m_i^\star, \boldsymbol{\pi}\right)}{\rho\left(\boldsymbol{a}_i^{(t-1)} \mid m_i^{(t-1)}, \boldsymbol{\pi}\right)} \frac{\rho\left(m_i^\star\right)}{\rho\left(m_i^{(t-1)}\right)}, \tag{3.30}$$

since $\prod_{j=1}^{J}\left\{\rho\left(y_{ij} \mid \boldsymbol{a}_i^\star\right)\right\} = \prod_{j=1}^{J}\left\{\rho\left(y_{ij} \mid \boldsymbol{a}_i^{(t-1)}\right)\right\} = 1$ by construction, and

$$\frac{q\left(\boldsymbol{a}_i^{(t-1)}, m_i^{(t-1)} \mid \boldsymbol{a}_i^\star, m_i^\star\right)}{q\left(\boldsymbol{a}_i^\star, m_i^\star \mid \boldsymbol{a}_i^{(t-1)}, m_i^{(t-1)}\right)} = \frac{q_m\left(m_i^{(t-1)} \mid \boldsymbol{a}_i^\star, m_i^\star\right)}{q_m\left(m_i^\star \mid \boldsymbol{a}_i^{(t-1)}, m_i^{(t-1)}\right)} \frac{q_a\left(\boldsymbol{a}_i^{(t-1)} \mid m_i^{(t-1)}, \boldsymbol{a}_i^\star, m_i^\star\right)}{q_a\left(\boldsymbol{a}_i^\star \mid m_i^\star, \boldsymbol{a}_i^{(t-1)}, m_i^{(t-1)}\right)}. \tag{3.31}$$

Each term on the right hand sides of equations (3.30) and (3.31) can be computed: $\rho\left(\boldsymbol{a}_i^\star \mid m_i^\star, \boldsymbol{\pi}\right)$ is a multinomial distribution (equation (3.8)), $\rho\left(m_i^\star\right)$ is one of the four prior distributions on the MOI (equations (3.10) to (3.13)), $q_m\left(m_i^{(t-1)} \mid \boldsymbol{a}_i^\star, m_i^\star\right)$ is equal to 1 or $1/2$ according to equation (3.23), $q_a\left(\boldsymbol{a}_i^{(t-1)} \mid m_i^{(t-1)}, \boldsymbol{a}_i^\star, m_i^\star\right)$ is equal to $p_{\text{add}ir}$ or $p^\star_{\text{sub}ir}$ according to equation (3.28), and likewise for the terms in the denominators (see, for example, figure 3.2). Note that although the notation does not make it explicit, the joint proposal (equation 3.16) is parameterised by $\boldsymbol{y}_i$, $m_{\max}$, $\boldsymbol{H}$ and $m_{i\min}$. These values do not feature in the notation, however, because they are fixed. Also note that there are no tuning parameters in the stage-wise proposals (equations

(3.17) and (3.24)), hence, under the Gibbs sampler described above, the acceptance rate of the update cannot be adjusted.

Update the vector of haplotype frequencies:

$\boldsymbol{\pi}$ is updated by sampling from its full conditional distribution with density,

$$\begin{aligned}
p(\boldsymbol{\pi} \mid \boldsymbol{a}^{(t)}, \boldsymbol{m}^{(t)}, \boldsymbol{y}) &\propto \prod_{i=1}^{\mathrm{I}} \left\{ p\left(\boldsymbol{a}_i^{(t)} \mid m_i^{(t)}, \boldsymbol{\pi}\right) \right\} p(\boldsymbol{\pi} \mid \boldsymbol{\alpha}), \\
&= \prod_{i=1}^{\mathrm{I}} \left\{ \mathscr{M}\text{ultinomial}\left(\boldsymbol{a}_i^{(t)} \mid m_i^{(t)}, \boldsymbol{\pi}\right) \right\} \mathscr{D}\text{irichlet}(\boldsymbol{\pi} \mid \boldsymbol{\alpha}), \\
&= \mathscr{D}\text{irichlet}\left(\boldsymbol{\pi} \mid \alpha_1 + \sum_{i=1}^{\mathrm{I}} a_{i1}^{(t)}, \dots, \alpha_R + \sum_{i=1}^{\mathrm{I}} a_{iR}^{(t)} \right).
\end{aligned}$$

Overview

The sampler is run for T iterations until convergence (see section 3.2.6). To spare computer memory, we sometimes set a thinning interval, meaning that only traces for each multiple of the thinning interval are retained. Following the general recommendation of Gelman *et al.* [88], we discard the first $t = 1, \dots, T/2$ traces as burnin, leaving the MCMC sample, $\{\boldsymbol{\pi}^n, \boldsymbol{a}^n, \boldsymbol{m}^n\}_{n=1}^N$, which approximates the joint posterior (equation (3.5)), where N is the size of the MCMC sample post burnin and thinning. The sample $\{\boldsymbol{\pi}^n\}_{n=1}^N$, which approximates $p(\boldsymbol{\pi} \mid \boldsymbol{y})$, is obtained by discarding $\boldsymbol{a}^n$ and $\boldsymbol{m}^n$ from the joint sample, $\{\boldsymbol{\pi}^n, \boldsymbol{a}^n, \boldsymbol{m}^n\}_{n=1}^N$. Note that the inverse of the likelihood function (equation (3.6)) maps $\{0, 1\}$ onto w, m or h conditional on $p_{ij} = (\boldsymbol{a}_i \cdot \boldsymbol{h}_j)/\sum_{r=1}^R a_{ir}$. By specifying an initial estimate of $\boldsymbol{a}_i^{(t)}$ at $t = 0$ for $i = 1, \dots, I$, $p_{ij}^{(t)}$ is also specified for $j = 1, \dots, J$, and thus each missing datum is assigned an initial estimate, $\hat{y}_{ij}^{(t)}$. Each time a new set of haplotypes is sampled for $t = 1, \dots, T$, new imputed values for the missing data, $\hat{y}_{ij}^t$, are assigned. Imputation in this way assumes that the probability that a datum is missing does not depend on its value and that the missing mechanism is 'ignorable'. In other words, the parameters governing the missingness mechanism (for example, DNA quantity) are

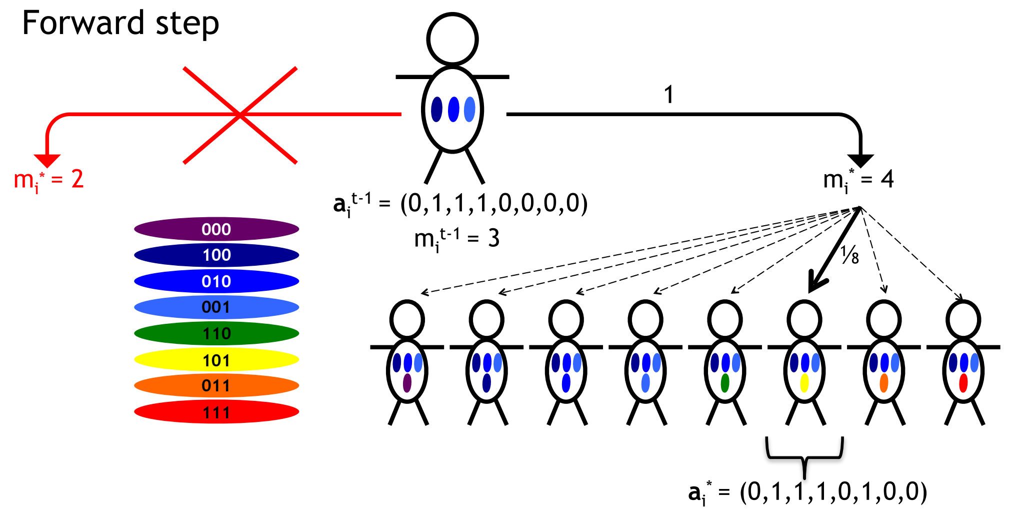

(a) Forward step. At iteration $t-1$ of the sampler, an individual with sample data $\boldsymbol{y}_i = (h,h,h)$ is thought to be infected with $m_i^{(t-1)} = 3$ clones with haplotypes '100', '010' and '001', hence $\boldsymbol{a}_i^{(t-1)} = (0_{000}, 1_{100}, 1_{010}, 1_{001}, 0_{110}, 0_{101}, 0_{011}, 0_{111})$. The proposal involves the addition of a clone with probability one (subtraction is prohibited since $m_{\text{masked}_i}^{(t-1)} = 0$, see $i=3$ equation (3.21)), hence $m_i^\star = 4$. All eight haplotypes are compatible with the observed data, hence $\boldsymbol{p}_{\text{add}\,i} = (1/8, \ldots, 1/8)$, again see $i=3$ equation (3.26)). We propose haplotype '101' (yellow ellipse), resulting in $\boldsymbol{a}_i^\star = (0_{000}, 1_{100}, 1_{010}, 1_{001}, 0_{110}, 1_{101}, 0_{011}, 0_{111},)$.

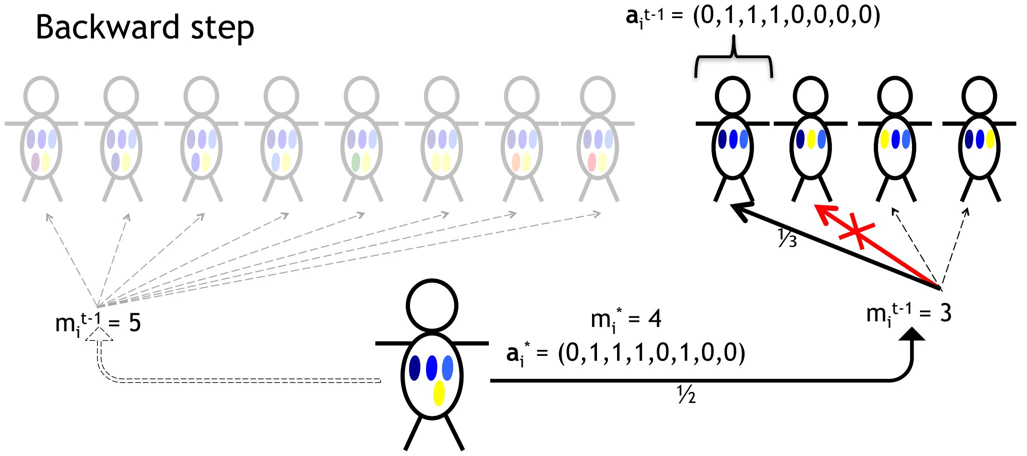

(b) Backward step. The reversal of the above proposal involves removal of the clone with haplotype '101' to recover $\boldsymbol{a}_i^{(t-1)}$. Following equations (3.18), (3.19) and (3.25), we have $\boldsymbol{a}_{\text{masked}_i}^\star = (0_{000}, 1_{100}, \mathbf{0}_{010}, 1_{001}, 0_{110}, 1_{101}, 0_{011}, 0_{111})$, $m_{\text{masked}_i}^\star = \sum_{r=1}^R a_{\text{masked}_{ir}}^\star = 3$ and $\boldsymbol{p}_{\text{sub}_i}^\star = {a_{\text{masked}_i}^\star}/{\sum_{r=1}^R a_{\text{masked}_{ir}}^\star} = (0, 1/3, 0, 1/3, 0, 1/3, 0, 0)$. In other words, the probability of removing a clone is $1/2$ since addition is also possible (we could go down the lefthand branch resulting in $m_i^\star = 5$, since $m_{\text{masked}_i}^\star > 0$ and $m_i^\star < m_{\max}$). We want to go down the righthand branch resulting in $m_i^{(t-1)} = 3$. Having chosen to remove a clone, the probability that we remove the clone with haplotype '101' (the yellow ellipse) is $1/3$, since we could also remove the clone with haplotype '100' (darkest blue ellipse) or the clone with haplotype '001' (lightest blue ellipse) without invalidating the compatibility of the ensuing vector of haplotype counts with the observed data.

Figure 3.2: A schematic of the proposal for the MOIs and haplotype counts. Malaria clones are represented by ellipses, colour-coded by haplotype (see stacked ellipse legend, subplot 3.2a). Branches with zero probability are depicted in red. They have zero probability because their outcomes violate compatibility with the observed data $\boldsymbol{y}_i = (h,h,h)$. Proposed branches are depicted by solid black lines. Alternative branches, that the proposal could have but did not take, are depicted by dashed lines. Each proposed branch is labeled by its probability, $\mathbb{P}(\text{proposed branch}) = 1/\text{number of available branches}$. The probabilities are equivalent to the terms in the proposal ratio, $q_m\left(m_i^{(t-1)} \mid \boldsymbol{a}_i^\star, m_i^\star\right) / q_m\left(m_i^\star \mid \boldsymbol{a}_i^{(t-1)}, m_i^{(t-1)}\right) \times q_a\left(\boldsymbol{a}_i^{(t-1)} \mid m_i^{(t-1)}, \boldsymbol{a}_i^\star, m_i^\star\right) / q_a\left(\boldsymbol{a}_i^\star \mid m_i^\star, \boldsymbol{a}_i^{(t-1)}, m_i^{(t-1)}\right) = {1/2}/{1} \times {1/3}/{1/8}$.

not related to the parameters of interest (the frequencies) [132]. This assumption, does not hold if genotyping fails because of an unanticipated allele. Since sequencing is often used to identify *de novo* mutations before genotyping, the ignorable assumption is likely to hold. However, if the proposed model is used to analyse genotyping data in which failed assay attempts are likely due to unanticipated alleles, samples with missing data should be discarded.

3.2.5 Simulated data

Data are simulated to enable assessment of model performance. A haplotype frequency vector is drawn from a uniform Dirichlet distribution. A stated number of blood samples per dataset are then generated as follows. Unless otherwise stated, for each blood sample, a MOI is drawn from a non-zero conditioned Poisson distribution with $\lambda = 3$. For each blood sample, the haplotype count vector is drawn from a multinomial distribution with size equal to the MOI and probability vector equal to the vector of haplotype frequencies. The haplotype frequencies in the simulated dataset are calculated. Unless otherwise stated, for each blood sample, an observation is generated assuming 100% detectability using the inverse of the likelihood function (equation (3.6)).

3.2.6 Convergence

For every run of the sampler, log-posterior and frequency trace plots are visually inspected to monitor convergence. In addition to habitual visual inspection, a preliminary study to assess the number of iterations required for convergence is performed using 50 simulated datasets comprising one to five SNPs and 100 blood samples. For each dataset, the sampler is for 10,000, 20,000 and 50,000 iterations ($50 \times 3 = 150$ analyses in total). For each analysis, the within and between sequence variances of three parallel chains, initialised at different initial frequency vectors, are compared. Initial frequency vectors are generated by setting all but one of the initial frequencies (selected at random) to 0.02. The remaining frequency is fixed such that the

frequencies sum to unity. For datasets with only one SNP, one of the chains is initialised from a frequency vector equal to (0.5, 0.5). Comparison is based on the potential scale reduction factor (PSRF), a metric of convergence recommended by Gelman *et al.* [88]. The PSRF is an indicator of the factor by which the discrepancy in variation might be reduced if the current chains are continued for an infinite number of iterations. A value close to one supports the conjecture that the chain has converged. Gelman *et al.* advise running the chain long enough such that every PSRF < 1.1, with higher precision for final analyses. The PSRF values reported in this chapter are calculated for each haplotype frequency according to the equations on pages 303 and 304 of [88]. In total, 50,000 iterations are found to be sufficient, taking approximately five minutes to analyse a dataset comprising 100 blood samples and five SNPs.

3.2.7 Sensitivity analyses

Model performance is assessed using a series of simulated datasets, investigating the precision and accuracy of the frequency point estimates as a function of the data. For each dataset, frequency point estimates are defined by the medians of the MCMC sample. Their 95% credible intervals range from the 2.5th to the 97.5th percentiles of the MCMC sample. Accuracy is defined as the absolute error between the point estimate and the true frequency in the simulated sample, while precision is defined by the standard deviation of the marginal MCMC sample. Note that this is non-standard counterintuitive (in that lower values correspond to more accurate and precise estimates), and that both accuracy and precision decrease with the number of SNPs because the frequency mass is shared over a greater number of haplotypes. We also investigate the sensitivity of the frequency estimates to missing data, their initial values, the MOI prior and the assumption of perfect detectability. It is important to note that the tabulated results in the following section are averaged over the frequency estimates within each analysis, as well as across the analyses of ten different datasets for each combination of variables investigated. Doing so accounts for variation in the haplotype frequencies and datasets, but may also mask

haplotype specific effects. To see how average results translate into specific estimates, for each dataset we plot the frequency point estimates and their 95% credible intervals (see for example figure 3.3). Additional details of the specific sensitivity analyses are outlined below.

Precision and accuracy as a function of the data: In total, 150 simulated datasets varying in both width (one to five SNPs) and height (50, 100 and 1000 blood samples) are analysed and the average frequency and precision of the point estimates calculated as outlined above. For comparison, the datasets are also analysed using an approximate method: all blood samples with one or more heteroallelic SNPs are discarded, leaving a dataset with no discernibly multiclonal blood samples from which frequencies could be directly calculated using proportions. The frequencies of any unobserved sequences are set to zero to ensure accuracy is averaged over the same number of haplotypes as under the model.

The sensitivity of the frequency point estimates to missing data: From each of the 50 datasets used to assess convergence (section 3.2.6) data are erased from 0, 25, 50 and 75 of the blood samples selected at random. The number of genotyping outcomes erased per blood sample is selected at random, so too are the outcomes erased. Given each level of erosion, the datasets are analysed twice: first opting to impute missing data and second opting to discard blood samples with incomplete data.

The sensitivity of the frequency point estimates to their initial values: Each of the 50 datasets used to assess convergence (section 3.2.6) are reanalysed. For datasets with only one SNP, results generated post running three parallel chains with initial frequency vectors equal to (0.02, 0.98), (0.08, 0.98) and (0.5, 0.5) are compared. For datasets with two to five SNPs, results from five different chains are compared. The initial frequency vectors are selected at random from a set of frequency vectors containing a vector of uniform frequencies and all vectors generated by setting all but one of the frequencies to 0.02.

The sensitivity of the frequency point estimates to the MOI prior specification: Each of the 50 datasets used to assess convergence (section 3.2.6) are reanalysed another three times: first incorrectly assuming the distribution over the MOI is uniform; second, incorrectly assuming it is a truncated negative binomial (with $\lambda = 3$ and $\phi = 0.5$); and third, incorrectly assuming it is truncated geometric (with $\lambda = 3$). The same 50 datasets are further reanalysed twice, this time correctly assuming a Poisson prior, but with $\lambda = 1$, and then $\lambda = 5$, instead of $\lambda = 3$.

The sensitivity of frequency point estimates to the assumption that all clones are detected equally: For one to five SNPs, ten cohorts of 100 blood samples are generated as outlined above (section 3.2.5) but with parameter λ equal to one, three, five and seven. Observations are then calculated: first assuming 100% detectability; second, assuming 90% detectability (minority alleles that contributed less than 10% to a given SNP are ignored); and finally assuming 70% detectability (minority alleles that contributed less than 30% to a given SNP are ignored). All the datasets ($5 \times 10 \times 4 \times 3 = 600$ in total) are analysed assuming 100% detectability.

3.3 Results

Precision and accuracy as a function of the data: As one would hope from a valid model and functioning sampler, for a given number of SNPs, precision and accuracy increase with the number of samples in the dataset (table 3.3). Importantly, for any given dataset, the accuracies of the estimates generated under the statistical model are superior to those generated by discarding multiclonal samples (table 3.3).

The sensitivity of the frequency point estimates to missing data: Unsurprisingly, for a given number of SNPs, the impact of missing data on the mean accuracy and precision of

Number of SNPs	Number of blood samples	Statistical model		Approximate method
		Precision	Accuracy	Accuracy
1	50	0.034	0.015	0.075
	100	0.027	0.016	0.101
	1000	0.010	0.006	0.083
2	50	0.038	0.022	0.092
	100	0.034	0.022	0.058
	1000	0.011	0.007	0.046
3	50	0.039	0.035	0.074
	100	0.029	0.022	0.047
	1000	0.010	0.007	0.026
4	50	0.032	0.024	0.053
	100	0.024	0.017	0.040
	1000	0.008	0.007	0.014
5	50	0.021	0.017	0.040
	100	0.017	0.013	0.028
	1000	0.007	0.006	0.010

Table 3.3: Precision and accuracy as a function of the width (number of SNPs) and height (number of samples) of the simulated datasets. Lower values are indicative of higher accuracy and precision. Note that due to the way accuracy and precision are defined (see the introductory paragraph to section 3.2.7), neither accuracy nor precision is comparable across different numbers of SNPs.

**Accuracy**	Number of blood samples with incomplete data			
Number of SNPs	0	25	50	75
1	0.16	0.20 (0.20)	0.34 (0.33)	0.42 (0.43)
2	0.23	0.28 (0.28)	0.32 (0.33)	0.40 (0.42)
3	0.23	0.26 (0.26)	0.24 (0.28)	0.37 (0.46)
4	0.17	0.20 (0.20)	0.22 (0.25)	0.31 (0.30)
5	0.13	0.14 (0.15)	0.15 (0.16)	0.17 (0.19)

Table 3.4: The impact of incomplete data upon the mean accuracy of the frequency estimates. Lower values indicate higher accuracy. For those datasets with missing data, the mean accuracy obtained from analyses based on only the blood samples with complete data are included in parentheses.

**Precision**	Number of blood samples with incomplete data			
Number of SNPs	0	25	50	75
1	0.27	0.31 (0.31)	0.38 (0.38)	0.54 (0.54)
2	0.35	0.39 (0.40)	0.42 (0.45)	0.54 (0.64)
3	0.29	0.32 (0.33)	0.36 (0.39)	0.45 (0.53)
4	0.24	0.26 (0.27)	0.29 (0.31)	0.33 (0.38)
5	0.17	0.18 (0.18)	0.19 (0.20)	0.21 (0.23)

Table 3.5: The impact of incomplete data upon the mean precision of the frequency estimates. Lower values indicate higher precision. For datasets with missing data, the mean precision obtained from analyses based on only the blood samples with complete data are included in parentheses.

the frequency point estimates is unfavourable (compare column two with columns three, four and five, tables 3.4 and 3.5, respectively). However, in general, estimates are more accurate and precise upon imputation (compare the numbers within and outside the parenthesis). In summary, imputation enables use of all available data, whereas, for datasets with two or more SNPs, partial data are squandered when blood samples with incomplete data are discarded.

The sensitivity of the frequency point estimates to their initial values: Frequency point estimates are robust to their initial values. The mean difference between estimates obtained from chains initiated at different values is < 0.01, while the maximum is 0.02 (haplotype 00000, dataset B, figure 3.3).

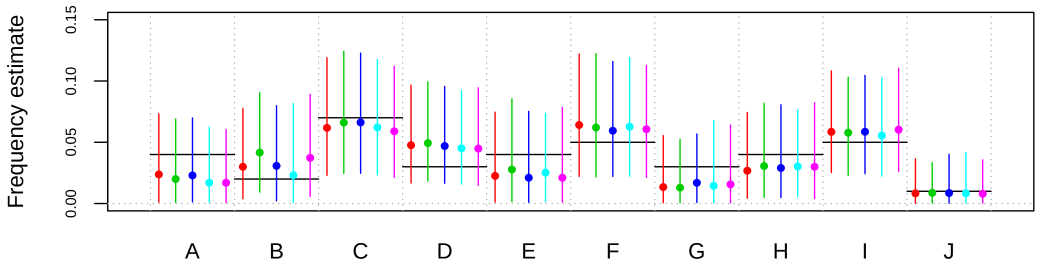

Figure 3.3: The sensitivity of a frequency point estimate to different initial values. The plot shows frequency point estimates (dots) with 95% credible intervals (vertical lines) for the haplotype with the allele sequence 00000, generated by five chains (five different colours) initiated at a different initial frequency vectors) for ten different datasets (A–J). For each dataset, the frequency of the haplotype with the allele sequence 00000 in the simulated sample is depicted by a black horizontal bar.

The sensitivity of the frequency point estimates to the MOI prior distribution: Unsurprisingly, the model with the correctly specified Poisson distribution over the MOI gives rise to the most accurate haplotype frequencies on average (table 3.6). At the level of the individual point estimates, the differences between estimates generated under the uniform, Poisson and negative binomial distributions are relatively small and the prior had little to no effect on precision (for example, see figure 3.4). Likewise, on average, the correct λ parameter specification gives rise to the most accurate frequency estimates (table 3.7). The detrimental effect of overestimating λ appears to be slightly less than that of underestimating it, but the range tested is small. In fact, it seems that no λ specification is preferable to misspecification (compare values in columns three and four of table 3.7 to values under the uniform prior in table 3.6). Overestimation has a spuriously favourable effect on precision, probably because overestimation augmented the number of clones per blood sample, thus leading to a greater number of haplotype assignments on which to base the haplotype frequencies. Sensitivity of the model to the parameter λ motivates the repeat analysis of field data, each time varying λ in order to establish the sensitivity of the results (for example, see section 4.2.3). Based on the accuracy when λ is unspecified compared with misspecified, if the *a priori* average MOI is unknown, a uniform prior is to be worth investigating.

Number of SNPs	MOI prior distribution			
	Uniform	Poisson	N. Binomial	Geometric
1	0.051	**0.016**	**0.016**	0.024
2	0.032	**0.023**	0.027	0.028
3	0.027	**0.022**	0.023	0.023
4	**0.017**	0.018	**0.017**	0.018
5	**0.013**	**0.013**	**0.013**	**0.013**

Table 3.6: The impact of MOI prior misspecification on the mean accuracy of the frequency estimates. All data are generated under a model with a MOI Poisson prior with $\lambda = 3$. Note that N. Binomial refers to a negative binomial distribution. Lower values (highlighted in bold) indicate higher accuracy.

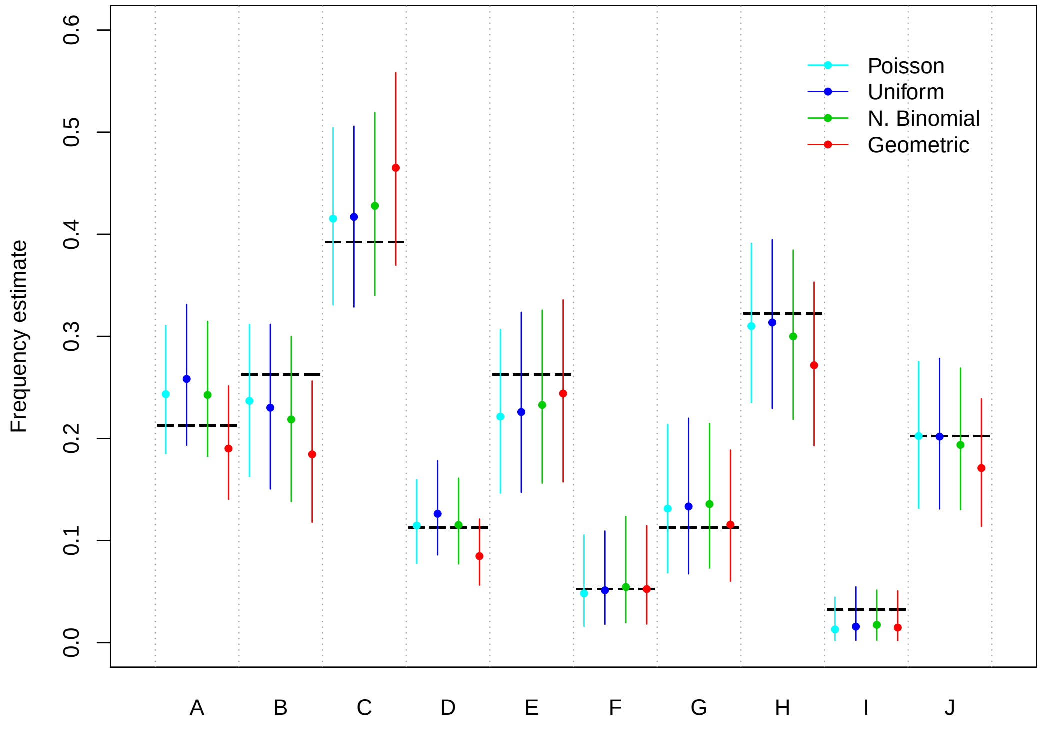

Figure 3.4: The impact of MOI prior misspecification on the frequency estimates of a single haplotype. The plot shows frequency point estimates (dots) and their 95% credible intervals (vertical lines) for the haplotype with allele sequence 11, colour-coded by the MOI prior distribution, across ten different datasets (A–J). The frequency in the simulated data set is denoted by the black horizontal bar. Note that N. Binomial refers to a negative binomial distribution. The data are simulated under the Poisson prior.

Number of SNPs	MOI prior parameter λ		
	Correct ($\lambda = 3$)	Underestimate ($\lambda = 1$)	Overestimate ($\lambda = 5$)
1	**0.016**	0.060	0.058
2	**0.023**	0.044	0.032
3	**0.023**	0.028	0.026
4	**0.017**	0.021	**0.017**
5	**0.013**	0.014	**0.013**

Table 3.7: The impact of the MOI prior parameter misspecification on the mean accuracy of the frequency estimates. Lower values indicate higher accuracy.

The sensitivity of frequency point estimates to the assumption that all clones are detected equally: Estimates are robust to suboptimal detectability when data are generated using MOI parameter, λ, of one or three (for example see figure 3.5). For data generated using $\lambda \geq 5$, estimates are robust to 90% detectability, but the accuracy decreases when the detectability drops to 70%, (figure 3.5). Unsurprisingly, the detrimental effect of suboptimal detectability has more impact upon datasets generated under a comparatively large MOI parameter, λ, since blood samples with a large number of clones are more likely to qualify as blood samples in which alleles might be in a minority. Suboptimal detectability appears to have a small spuriously positive effect on precision, seemingly due to the relative decline in the number of heteroallelic alleles. Suboptimal detectability primarily affects datasets comprised of three or fewer SNPs (table 3.8). In addition to the method used to define accuracy (see introductory paragraph to section 3.2.7), this may, in part, be due to the method used to generate the data, explained as follows. The data are generated using a frequency vector drawn from a uniform Dirichlet distribution. Since the number of possible haplotypes increases exponentially with the number of SNPs, haplotypes frequencies tend to be more uniform in datasets comprised of four or more SNPs. Since the likelihood that a single SNP is dominated by a single allele is smaller in a dataset comprising a large number of haplotypes over which mass is evenly distributed, suboptimal detectability primarily affects datasets comprised of three or fewer SNPs, especially

Number of SNPs	MOI parameter, λ	Limit of detection		
		100%	90%	70%
1	1	0.011	0.011	0.012
	3	0.015	0.015	0.051
	5	0.012	0.012	0.107
	7	0.033	0.029	0.160
2	1	0.017	0.017	0.017
	3	0.018	0.018	0.041
	5	0.028	0.028	0.081
	7	0.035	0.031	0.123
3	1	0.011	0.011	0.011
	3	0.019	0.019	0.025
	5	0.028	0.025	0.049
	7	0.039	0.04	0.065
4	1	0.010	0.010	0.010
	3	0.016	0.016	0.017
	5	0.023	0.023	0.028
	7	0.033	0.033	0.031
5	1	0.009	0.009	0.009
	3	0.013	0.014	0.014
	5	0.017	0.017	0.016
	7	0.019	0.019	0.018

Table 3.8: The impact of suboptimal detectability on the accuracy of the frequency estimates. For one to five SNPs, ten datasets are generated given detectability equal to 100%, 90% and 70%. The datasets are analysed assuming optimal detectability (100%). Lower values indicate higher accuracy.

when mass is unevenly distributed (for example, see the estimate for haplotype 010, cohort 26, $\lambda = 5$, figure 3.5).

3.4 Discussion

In this chapter, we present a statistical model designed to estimate population-level frequencies of *P. falciparum* allele and multi-SNP haplotype and genotype frequencies using prevalence data from malaria endemic regions where multiclonal infections are commonplace. Multiclonal

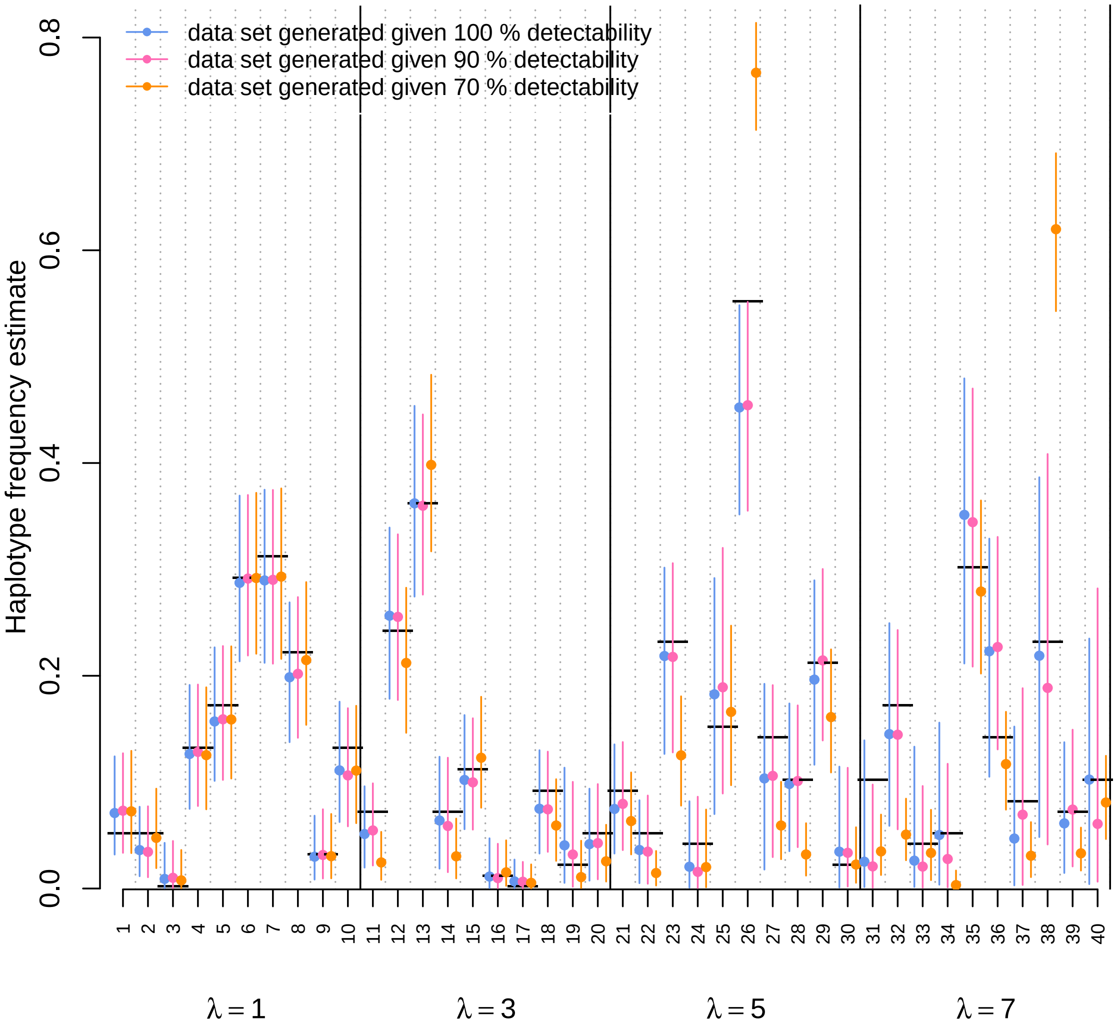

Figure 3.5: The impact of suboptimal detectability on the frequency estimates for a single haplotype with allele sequence 010. The plot shows haplotype frequency estimates (points) and their 95% credible intervals (vertical lines), given suboptimal detectability. Ten cohorts of one hundred infected blood samples are generated per specified MOI parameter, λ (40 cohorts in total: 1–10, $\lambda = 1$; 11–20, $\lambda = 3$; 21–30, $\lambda = 5$; 31–40, $\lambda = 7$). To enable comparison between results given different limits of detectability, three datasets are generated per cohort: one given 100% detectability (blue), another given 90% detectability (pink), and another given 70% detectability (orange). Each of the three datasets generated from a common cohort have the same frequencies in the simulated datasets (black horizontal bar), since the haplotypes in the infected blood samples remained the same despite changing the detectability. The datasets are all analysed assuming 100% detectability.

infections hamper the genetic surveillance of antimalarial resistance. The model is designed to overcome the problems associated with multiclonal infections. Its application generates comparable frequency estimates, allowing markers of resistance to be tracked in malaria endemic regions, yielding important information on the dynamics of resistance.

Importantly, the model does not require measurements of the sample-wise MOIs. Instead, it uses an *a priori* estimate of the average MOI (based on auxiliary data where available), an initial estimate of the vector of haplotype frequencies, and all available prevalence data to infer the haplotypes of the unobserved clones within individual blood samples. The initial frequency estimates are then revised based on clonal assignments. The implementation algorithm cycles over the aforementioned steps thousands of times until convergence. Application of the model reconstructs haplotypes within samples, provides a consistent method of frequency estimation, and avoids the loss of information that results from the usual adjustments made for multiclonal blood samples and unsuccessful genotyping outcomes.

To assess the impact of various model choices a suite of sensitivity analyses is performed using simulated data. The simulation study demonstrates that the frequencies estimated using the model are more accurate than estimates based on simply calculating proportions after discarding discernibly multiclonal blood samples. The model is robust to changes in the initial frequency estimates, but sensitive to deviations in the prior on the MOI. In light of these results, we recommend an investigation to find the MOI prior distribution that provides the best fit to the data (see section A.2 for an example), followed by repeat analyses of the data, each time varying the MOI prior parameter value within a reasonable range (such as the limits of its 95% confidence interval), to establish the sensitivity of the results to its value (see section 4.2.3 in the following chapter).

A number of simplifying assumptions are made in the construction of the model; they are listed below. As with any model, it is important to note that although the assumptions are likely to be violated in practice, the model may still be useful, as famously remarked upon by George

Box [31]. The assumptions include

1. blood samples are independently distributed;
2. clones are independently distributed (for example, the probability of being infected with two clones with allelic sequences '000' and '011' is $\pi_{000} \times \pi_{011}$);
3. perfect detection (for example, if a person is infected with ten clones, nine of which are characterised by '000' and one by '100', the mutant allele is detected);
4. alleles are error-free (for example '0' is correctly identified as '0' and not as '1').

The first two assumptions are common to all of the existing statistical methods of *P. falciparum* haplotype frequency estimation [102, 224, 129, 95, 276, 125], while more realistic assumptions regarding detectability and SNP miscalls are incorporated into alternative models ([95] and [276], respectively). We now discuss each assumption in turn.

Depending on the study design, the assumption of independence between samples is a valid one. For example, blood samples surveyed in a cross sectional study (such as [22]) should be independent. On the contrary, repeat sampling from the same child (for an example, see [51]) might lead to dependence. In chapter 4, we analyse the data from [51] under the assumption of independence. In chapter 5, we relax the assumption by adding an extension to our model.

The assumption that clones are independent depends on the manner in which multiclonal infections are acquired. An individual infected with clones obtained from multiple successive bites in a high transmission setting is likely to harbour independent clones, whereas the assumption is unlikely to hold for a person infected with multiple clones following a single inoculation from a mosquito harbouring a multiclonal infection [102]. Since both mechanisms are likely to occur, especially in high transmission settings, the assumption that clones are independently distributed is questionable. Reasons as to why the assumption might not harm inference are discussed in length by Hill and Babiker [102]. Perhaps the most compelling argument put forward by Hill and Babiker is the agreement between the experimentally-derived within-vector diversity (based on diploid oocysts from dissected mosquitoes collected in

the same village as the prevalence data), and the within-host diversity estimated under the assumption of independence. We agree with Hill and Babiker that the assumption is tenuous but pragmatic, noting (as do they) that there is not enough information in the data to support a model that distinguishes between inoculation with recombinant and independent clones [102]. In addition, we note that dependence between haplotypes will unlikely harm average estimates, since correlation typically leads to over-dispersed but unbiased realisations (see binomial example in [84]).

The validity of the assumption that all SNPs are correctly identified depends on the technology used to generate the data and differs for different SNPs. For example, concordances between calls based on microarray technology and calls based on RFLP analyses ranging from 63.9% (for *pdfhfr*-51) to 100% (for *pfmdr1*-86 and *pfdhps*-581) have been reported [145]. The model by Wigger *et al.*, includes an error probability term, which is fixed and equal for all SNPs [276] (see chapter 2 for a full description). Based on simulated data, Wigger *et al.* conclude that the error model is beneficial if the miscall rate exceeds 1–2%. Following Wigger *et al.*, it would be interesting to incorporate an error term into our model to account for miscalled SNPs. It is noted, however, that when analysing microarray data from the field (in which the probability of an error is thought to be 0.05 based on SNP-wise comparison with RFLP base calls [145]), the frequency estimates are statistically indifferent unless a large number of samples (> 500) are analysed [276]. If a large number of samples are analysed, omission of a fixed random error is likely to cause the model to overfit noise, hence underestimate dominant frequencies and overestimate rare frequencies [276]. A simple way to avoid overfitting noise without adding an error term, is to analyse the data twice, setting rare frequencies to zero in the second analysis [276].

The assumption that all clones in the blood sample are perfectly detected is almost certainly violated, especially when analysing data generated by PCR based methods [96]. Detection limits are thought to range between 80% and 99% [133, 113, 114, 58]. To assess the impact

of this assumption, simulated data are generated under imperfect detectability. Our model is robust to imperfect detection, providing the MOI prior parameter, λ, is less than or equal to three, or the limit of detectability is 90% or more.

Further to the problem of imperfect detection, is the fact that the blood sample itself might not contain a representative sample of the infection. This might occur because of low parasite numbers and/or because *P. falciparum* infections undergo complex cycles of sequestration [77]. Even when imperfect detection due to experimental procedures is taken into account (see [95], for example), what actually is estimated is the proportion of accessible parasite clones among the within-host parasite population. However, if the parameters of sequestration are independent to the frequencies of interest, which we assume they are, the inaccessible clones are ignorable and the estimates based on the accessible clones should be accurate; that is to say, collectively, the blood samples should equitably represent the host-infecting parasite population.

Following Hastings *et al.* [95, 96], we define frequency in terms of parasite clones (recall that, following convention, we use the word clone to denote a collection of genetically identical parasites). Alternatively, one could define frequency in terms of the proportion of parasites. In fact, one could see the former definition as an approximation of the latter, assuming clones represent populations of equal size. Unfortunately, there is not enough information in prevalence data to support a model that accommodates estimates in terms of biomass. Hence, all models that generate *P. falciparum* allele, haplotype and genotype frequencies based on prevalence data either define frequency in terms of parasite clones, or assume clones represent clones of equal size [39, 102, 224, 129, 95, 276, 125, 223].

As outlined in the introduction, several differences set our model apart from existing methods of *P. falciparum* allele, haplotype and genotype estimation using prevalence data. In contrast to preceding methods, the model presented here is able to analyse prevalence data for more than three SNPs, using all available data, including those that are incomplete due

to unsuccessful genotyping assays, without reliance upon experimentally-derived estimates of the sample-wise MOI, within a Bayesian framework, thus providing a readily extendable framework in which uncertainty is captured in a straightforward yet comprehensive manner. However, superior assumptions regarding detectability and experimental error are incorporated into alternative models [95, 276] (see above). It is especially important to take into account the suboptimal detectability of minority clones, addressed by Hastings *et al.* [95], when the experimentally-derived MOI estimates are regarded as fixed [96]. The latter is not the case in the current model (patient-level MOIs are treated as unobserved random variables), perhaps explaining why our model is comparatively robust to imperfect detectability.

In summary, genetic monitoring of *P. falciparum* plays an important role in the timely surveillance of antimalarial drug resistance. However, multiclonal infections present an analytic challenge, especially in areas of high transmission. We provide a full description of a model designed to overcome the challenge of multiclonal infections and estimate the frequencies of *P. falciparum* allele, multi-SNP haplotypes and genotypes. Its validity is demonstrated using a suite of sensitivity analyses, while its utility is demonstrated elsewhere using prevalence data for markers of resistance to SP [248]. Its applicability, however, extends beyond markers of SP resistance, as demonstrated in the following chapter. To the best of our knowledge, this is the first model that combines rapid analysis of three or more SNPs, using all available data without reliance upon measurements of the MOI in individual blood samples.

References

[1] I. E. A-Elbasit, G. ElGhazali, T. M. E. A-Elgadir, A. A. Hamad, H. A. Babiker, M. I. Elbashir, and H. A. Giha. Allelic polymorphism of MSP2 gene in severe P. falciparum malaria in an area of low and seasonal transmission. *Parasitology Research*, 102:29–34, 2007.

[2] A. O. Achieng, P. Muiruri, L. A. Ingasia, B. H. Opot, D. W. Juma, R. Yeda, B. S. Ngalah, B. R. Ogutu, B. Andagalu, H. M. Akala, and E. Kamau. Temporal trends in prevalence of Plasmodium falciparum molecular markers selected for by artemether–lumefantrine treatment in pre-ACT and post-ACT parasites in western Kenya. *International Journal for Parasitology: Drugs and Drug Resistance*, 5:92–99, 2015.

[3] D. Aguiar, W. S. W. Wong, and S. Istrail. Tumor haplotype assembly algorithms for cancer genomics. *Pacific Symposium on Biocomputing*, 2014.

[4] J. Albert. *Bayesian Computation with R*. Springer, 2 edition, 2009.

[5] M. Alifrangis, M. M. Lemnge, A. M. Rønn, M. D. Segeja, S. M. Magesa, I. F. Khalil, and I. C. Bygbjerg. Increasing prevalence of wildtypes in the dihydrofolate reductase gene of Plasmodium falciparum in an area with high levels of sulfadoxine/pyrimethamine resistance after introduction of treated bed nets. *The American journal of tropical medicine and hygiene*, 69(3):238–43, 2003.

[6] M. Alifrangis, S. Enosse, R. Pearce, C. Drakeley, C. Roper, I. F. Khalil, W. M. Nkya, A. M. Rønn, T. G. Theander, and I. C. Bygbjerg. A simple, high-throughput method to detect Plasmodium falciparum single nucleotide polymorphisms in the dihydrofolate reductase, dihydropteroate synthase, and P. falciparum chloroquine resistance transporter genes using polymerase chain reaction- and enzy. *The American journal of tropical medicine and hygiene*, 72(2):155–62, 2005.

[7] C. Amaratunga, S. Sreng, S. Suon, E. S. Phelps, K. Stepniewska, P. Lim, C. Zhou, S. Mao, J. M. Anderson, N. Lindegardh, H. Jiang, J. Song, X. Z. Su, N. J. White, A. M. Dondorp, T. J. C. Anderson, M. P. Fay, J. Mu, S. Duong, and R. M. Fairhurst. Artemisinin-resistant Plasmodium falciparum in Pursat province, western Cambodia: A parasite clearance rate study. *The Lancet Infectious Diseases*, 12:851–858, 2012.

[8] L. N. Amenga-Etego. *Plasmodium falciparum population genetics in northern Ghana*. PhD thesis, University of Oxford, 2012.

[9] T. J. C. Anderson, X. Z. Su, M. Bockarie, M. Lagog, and K. P. Day. Twelve microsatellite markers for characterization of Plasmodium falciparum from finger-prick blood samples. *Parasitology*, 119(02):113–25, 1999.

[10] T. J. Anderson, B. Haubold, J. T. Williams, J. G. Estrada-Franco, L. Richardson, R. Mollinedo, M. Bockarie, J. Mokili, S. Mharakurwa, N. French, J. Whitworth, I. D. Velez, A. H. Brockman, F. Nosten, M. U. Ferreira, and K. P. Day. Microsatellite markers reveal a spectrum of population structures in the malaria parasite Plasmodium falciparum. *Molecular biology and evolution*, 17(10):1467–82, 2000.

[11] T. J. C. Anderson, S. Nair, D. Sudimack, J. T. Williams, M. Mayxay, P. N. Newton, J.-P. Guthmann, F. M. Smithuis, T. Tinh Hien, I. V. F. van den Broek, N. J. White, and F. Nosten. Geographical distribution of selected and putatively neutral SNPs in Southeast Asian malaria parasites. *Molecular biology and evolution*, 22(12):2362–74, 2005.

[12] T. J. C. Anderson, J. Patel, and M. T. Ferdig. Gene copy number and malaria biology. *Trends in parasitology*, 25(7):336–343, 2009.

[13] F. Ariey, T. Fandeur, R. Durand, M. Randrianarivelojosia, R. Jambou, E. Legrand, M. T. Ekala, C. Bouchier, S. Cojean, J. B. Duchemin, V. Robert, J. Le Bras, and O. Mercereau-Puijalon. Invasion of Africa by a single pfcrt allele of South East Asian type. *Malaria Journal*, 5:34–39, 2006.

[14] F. Ariey, B. Witkowski, C. Amaratunga, J. Beghain, A.-C. Langlois, N. Khim, S. Kim, V. Duru, C. Bouchier, L. Ma, P. Lim, R. Leang, S. Duong, S. Sreng, S. Suon, C. M. Chuor, D. M. Bout, S. Ménard, W. O. Rogers, B. Genton, T. Fandeur, O. Miotto, P. Ringwald, J. Le Bras, A. Berry, J.-C. Barale, R. M. Fairhurst, F. Benoit-Vical, O. Mercereau-Puijalon, and D. Ménard. A molecular marker of artemisinin-resistant Plasmodium falciparum malaria. *Nature*, 505:50–55, 2014.

[15] E. Arinaitwe, T. G. Sandison, H. Wanzira, A. Kakuru, J. Homsy, J. Kalamya, M. R. Kamya, N. Vora, B. Greenhouse, P. J. Rosenthal, J. Tappero, and G. Dorsey. Artemether-lumefantrine versus dihydroartemisinin-piperaquine for falciparum malaria: a longitudinal, randomized trial in young Ugandan children. *Clinical infectious diseases : an official publication of the Infectious Diseases Society of America*, 49:1629–37, 2009.

[16] E. A. Ashley, M. Dhorda, R. M. Fairhurst, C. Amaratunga, P. Lim, S. Suon, S. Sreng, J. M. Anderson, S. Mao, B. Sam, C. Sopha, C. M. Chuor, C. Nguon, S. Sovannaroth, S. Pukrittayakamee, P. Jittamala, K. Chotivanich, K. Chutasmit, C. Suchatsoonthorn, R. Runcharoen, T. T. Hien, N. T. Thuy-Nhien, N. V. Thanh, N. H. Phu, Y. Htut, K.-T. Han, K. H. Aye, O. A. Mokuolu, R. R. Olaosebikan, O. O. Folaranmi, M. Mayxay, M. Khanthavong, B. Hongvanthong, P. N. Newton, M. A. Onyamboko, C. I. Fanello, A. K. Tshefu, N. Mishra, N. Valecha, A. P. Phyo, F. Nosten, P. Yi, R. Tripura, S. Borrmann, M. Bashraheil, J. Peshu, M. A. Faiz, A. Ghose, M. A. Hossain, R. Samad, M. R. Rahman, M. M. Hasan, A. Islam, O. Miotto, R. Amato, B. MacInnis, J. Stalker, D. P. Kwiatkowski, Z. Bozdech, A. Jeeyapant, P. Y. Cheah, T. Sakulthaew, J. Chalk, B. Intharabut, K. Silamut, S. J. Lee, B. Vihokhern, C. Kunasol, M. Imwong, J. Tarning, W. J. Taylor, S. Yeung, C. J. Woodrow, J. A. Flegg, D. Das, J. Smith, M. Venkatesan, C. V. Plowe, K. Stepniewska, P. J. Guerin, A. M. Dondorp, N. P. Day, and N. J. White. Spread of Artemisinin Resistance in Plasmodium falciparum Malaria. *New England Journal of Medicine*, 371:411–423, 2014.

[17] S. A. Assefa, M. D. Preston, S. Campino, H. Ocholla, C. J. Sutherland, and T. G. Clark. estMOI: Estimating multiplicity of infection using parasite deep sequencing data. *Bioinformatics*, 30(9):1292–1294, 2014.

[18] I. Astrovskaya, N. Manusco, B. Tork, S. Mangul, A. Artyomenko, P. Skums, L. Ganova-Raeva, I. Mandoiu, and A. Zelikovsky. Inferring viral quasispecies spectra from shortgun and aplicon next-generation sequencing reads. In M. S. Poptsova, editor, *Genome analysis: current procedures and applications*, chapter 12. Caister Academic Press, 2014.

[19] S. Auburn, S. Campino, O. Miotto, A. A. Djimde, I. Zongo, M. Manske, G. Maslen, V. Mangano, D. Alcock, B. MacInnis, K. A. Rockett, T. G. Clark, O. K. Doumbo, J. B. Ouédraogo, and D. P. Kwiatkowski. Characterization of within-host Plasmodium falciparum diversity using next-generation sequence data. *PloS one*, 7(2):e32891, 2012.

[20] C. Aurrecoechea, J. Brestelli, B. P. Brunk, J. Dommer, S. Fischer, B. Gajria, X. Gao, A. Gingle, G. Grant, O. S. Harb, M. Heiges, F. Innamorato, J. Iodice, J. C. Kissinger, E. Kraemer, W. Li, J. A. Miller, V. Nayak, C. Pennington, D. F. Pinney, D. S. Roos, C. Ross, C. J. Stoeckert Jr, C. Treatman, and H. Wang. PlasmoDB: a functional genomic database for malaria parasites. *Nucleic acids research*, 37(suppl 1), 2008.

[21] J. K. Baird. Resurgent malaria at the millennium: control strategies in crisis. *Drugs*, 59(4):719–43, 2000.

[22] V. Baraka, D. S. Ishengoma, F. Fransis, D. T. R. Minja, R. A. Madebe, D. Ngatunga, and J.-P. Van Geertruyden. High-level Plasmodium falciparum sulfadoxine-pyrimethamine resistance with the concomitant occurrence of septuple haplotype in Tanzania. *Malaria Journal*, 14:439, 2015.

[23] H. P. Beck, I. Felger, W. Huber, S. Steiger, T. Smith, N. Weiss, P. Alonso, and M. Tanner. Analysis of multiple Plasmodium falciparum infections in Tanzanian children during the phase III trial of the malaria vaccine SPf66. *The Journal of infectious diseases*, 175:921–6, 1997.

[24] M. S. Beier, I. K. Schwartz, J. C. Beier, P. V. Perkins, F. Onyango, J. K. Koros, G. H. Campbell, P. M. Andrysiak, and A. D. Brandling-Bennett. Identification of malaria species by ELISA in sporozoite and oocyst infected Anopheles from western Kenya. *The American Journal of Tropical Medicine and Hygiene*, 39(4):323–327, 1988.

[25] O. Bembom. seqLogo: Sequence logos for DNA sequence alignments. *R package version 1.36.0.*

[26] E. Berger, D. Yorukoglu, J. Peng, and B. Berger. HapTree: a novel Bayesian framework for single individual polyplotyping using NGS data. *PLoS computational biology*, 10(3):e1003502, 2014.

[27] S. Bhatt, D. J. Weiss, E. Cameron, D. Bisanzio, B. Mappin, U. Dalrymple, K. E. Battle, C. L. Moyes, A. Henry, M. A. Penny, T. A. Smith, A. Bennett, J. Yukich, T. P. Eisele, P. A. Eckhoff, E. A. Wenger, O. Brie, J. T. Griffin, C. A. Fergus, M. Lynch, F. Lindgren, J. M. Cohen, C. L. J. Murray, D. L. Smith, S. I. Hay, R. E. Cibulskis, and P. W. Gething. The effect of malaria control on Plasmodium falciparum in Africa between 2000 and 2015. *Nature*, 526, 2015.

[28] Y. L. Boo, H. T. Lim, P. W. Chin, S. Y. Lim, and F. K. Hoo. A case of severe Plasmodium knowlesi in a splenectomized patient. *Parasitology international*, 65(1):55–57, 2015.

[29] S. Borrmann, J. Straimer, L. Mwai, A. Abdi, A. Rippert, J. Okombo, S. Muriithi, P. Sasi, M. M. Kortok, B. Lowe, S. Campino, S. Assefa, S. Auburn, M. Manske, G. Maslen, N. Peshu, D. P. Kwiatkowski, K. Marsh, A. Nzila, and T. G. Clark. Genome-wide screen identifies new candidate genes associated with artemisinin susceptibility in Plasmodium falciparum in Kenya. *Scientific reports*, 3:3318, 2013.

[30] M. K. Bouyou-Akotet, N. P. M'Bondoukwé, and D. P. Mawili-Mboumba. Genetic polymorphism of merozoite surface protein-1 in Plasmodium falciparum isolates from patients with mild to severe malaria in Libreville, Gabon. *Parasite*, 22(12), 2015.

[31] G. E. P. Box and N. R. Draper. *Empirical Model-Building and Response Surfaces*. Wiley New York, 1987.

[32] B. J. Brabin. An analysis of malaria in pregnancy in Africa. *Bulletin of the World Health Organization*, 61(6):1005–1016, 1983.

[33] M. T. Bretscher, F. Valsangiacomo, S. Owusu-Agyei, M. A. Penny, I. Felger, and T. Smith. Detectability of Plasmodium falciparum clones. *Malaria Journal*, 9:234, 2010.

[34] D. R. Brooks, P. Wang, M. Read, W. M. Watkins, P. F. Sims, and J. E. Hyde. Sequence variation of the hydroxymethyldihydropterin pyrophosphokinase: dihydropteroate synthase gene in lines of the human malaria parasite, Plasmodium falciparum, with differing resistance to sulfadoxine. *European journal of biochemistry*, 224(2):397–405, 1994.

[35] S. Brooks, A. Gelman, G. Jones, and X. Meng, editors. *Handbook of Markov Chain Monte Carlo*. CRC press, 2011.

[36] S. R. Browning and B. L. Browning. Haplotype phasing: existing methods and new developments. *Nature reviews. Genetics*, 12(10):703–714, 2011.

[37] S. Campino, S. Auburn, K. Kivinen, I. Zongo, J.-B. Ouedraogo, V. Mangano, A. Djimde, O. K. Doumbo, S. M. Kiara, A. Nzila, S. Borrmann, K. Marsh, P. Michon, I. Mueller, P. Siba, H. Jiang, X.-Z. Su, C. Amaratunga, D. Socheat, R. M. Fairhurst, M. Imwong, T. Anderson, F. Nosten, N. J. White, R. Gwilliam, P. Deloukas, B. MacInnis, C. I. Newbold, K. Rockett, T. G. Clark, and D. P. Kwiatkowski. Population genetic analysis of Plasmodium falciparum parasites using a customized Illumina GoldenGate genotyping assay. *PLoS ONE*, 6(6):e20251, 2011.

[38] V. I. Carrara, J. Zwang, E. A. Ashley, R. N. Price, K. Stepniewska, M. Barends, A. Brockman, T. Anderson, R. McGready, L. Phaiphun, S. Proux, M. van Vugt, R. Hutagalung, K. M. Lwin, A. P. Phyo, P. Preechapornkul, M. Imwong, S. Pukrittayakamee, P. Singhasivanon, N. J. White, and F. Nosten. Changes in the treatment responses to artesunate-mefloquine on the northwestern border of Thailand during 13 years of continuous deployment. *PloS one*, 4(2):e4551, 2009.

[39] R. Carter and I. A. Mcgregor. Enzyme Variation in Plasmodium Falciparum in the Gambia. *Trans R Soc Trop Med Hyg*, 67(6):830–837, 1973.

[40] R. Carter and K. N. Mendis. Evolutionary and historical aspects of the burden of malaria. *Clinical microbiology reviews*, 15(4):564–594, 2002.

[41] L. K. Certain and C. H. Sibley. Plasmodium falciparum: a novel method for analyzing haplotypes in mixed infections. *Experimental parasitology*, 115(3):233–241, 2007.

[42] S. Chaorattanakawee, D. L. Saunders, D. Sea, N. Chanarat, K. Yingyuen, S. Sundrakes, P. Saingam, N. Buathong, S. Sriwichai, S. Chann, Y. Se, Y. Yom, T. K. Heng, N. Kong, W. Kuntawunginn, K. Tangthongchaiwiriya, C. Jacob, S. Takala-Harrison, C. Plowe, J. T. Lin, C. M. Chuor, S. Prom, S. D. Tyner, P. Gosi, P. Teja-Isavadharm, C. Lon, and C. A. Lanteri. Ex vivo drug susceptibility testing and molecular profiling of clinical Plasmodium falciparum isolates from Cambodia from 2008 to 2013 suggest emerging piperaquine resistance. *Antimicrobial Agents and Chemotherapy*, 59(8):4631–4643, 2015.

[43] I. H. Cheeseman, B. A. Miller, S. Nair, S. Nkhoma, A. Tan, J. C. Tan, S. Al Saai, A. P. Phyo, C. L. Moo, K. M. Lwin, R. McGready, E. Ashley, M. Imwong, K. Stepniewska, P. Yi, A. M. Dondorp, M. Mayxay, P. N. Newton, N. J. White, F. Nosten, M. T. Ferdig, and T. J. C. Anderson. A major genome region underlying artemisinin resistance in malaria. *Science*, 336:79–82, 2012.

[44] I. H. Cheeseman, M. McDew-White, A. P. Phyo, K. Sriprawat, F. Nosten, and T. J. Anderson. Pooled sequencing and rare variant association tests for identifying the determinants of emerging drug resistance in malaria parasites. *Mol Biol Evol*, 32(4):1080–1090, 2015.

[45] S. J. Cheesman, J. C. de Roode, A. F. Read, and R. Carter. Real-time quantitative PCR for analysis of genetically mixed infections of malaria parasites: technique validation and applications. *Molecular and Biochemical Parasitology*, 131:83–91, 2003.

[46] L. M. Childs and C. O. Buckee. Dissecting the determinants of malaria chronicity: why within-host models struggle to reproduce infection dynamics. *Journal of the Royal Society, Interface*, 12(104), 2015.

[47] W. Chin, P. G. Contacos, G. R. Coatney, and H. K. King. The evaluation of sulfonamides, alone or in combination with pyrimethamine, in the treatment of multi-resistant falciparum malaria. *American Journal of Tropical Medicine and Hygiene*, 15(6), 1966.

[48] R. H. B. Christensen. ordinal—Regression Models for Ordinal Data. *R package version 2015.6-28. http://www.cran.r-project.org/package=ordinal/*, 2015.

[49] T. D. Clark, B. Greenhouse, D. Njama-Meya, B. Nzarubara, C. Maiteki-Sebuguzi, S. G. Staedke, E. Seto, M. R. Kamya, P. J. Rosenthal, and G. Dorsey. Factors determining the heterogeneity of malaria incidence in children in Kampala, Uganda. *The Journal of infectious diseases*, 198(3):393–400, 2008.

[50] D. Clyde and G. Shute. Resistance of Plasmodium falciparum in Tanganyika to pyrimethamine administered at weekly intervals. *Transactions of the Royal Society of Tropical Medicine and Hygiene*, 51(6):505–513, 1957.

[51] M. D. Conrad, N. Leclair, E. Arinaitwe, H. Wanzira, A. Kakuru, V. Bigira, M. Muhindo, M. R. Kamya, J. W. Tappero, B. Greenhouse, G. Dorsey, and P. J. Rosenthal. Comparative impacts over 5 years of artemisinin-based combination therapies on P.falciparum polymorphisms that modulate drug sensitivity in Ugandan children. *The Journal of infectious diseases*, 210(3):344–353, 2014.

[52] D. J. Conway, B. M. Greenwood, and J. S. McBride. The epidemiology of multiple-clone Plasmodium falciparum infections in Gambian patients. *Parasitology*, 130(01):1–5, 1991.

[53] J. F. Cortese, A. Caraballo, C. E. Contreras, and C. V. Plowe. Origin and dissemination of Plasmodium falciparum drug-resistance mutations in South America. *The Journal of infectious diseases*, 186(7):999–1006, 2002.

[54] A. F. Cowman, M. J. Morry, B. A. Biggs, G. A. Cross, and S. J. Foote. Amino acid changes linked to pyrimethamine resistance in the dihydrofolate reductase-thymidylate synthase gene of Plasmodium falciparum. *Proceedings of the National Academy of Sciences*, 85(23):9109–9113, 1988.

[55] S. L. Croft, S. Duparc, S. J. Arbe-Barnes, J. C. Craft, C.-S. Shin, L. Fleckenstein, I. Borghini-Fuhrer, and H.-J. Rim. Review of pyronaridine anti-malarial properties and product characteristics. *Malaria Journal*, 11(270), 2012.

[56] S. Dahlström, P. E. Ferreira, M. I. Veiga, N. Sedighi, L. Wiklund, A. Mårtensson, A. Färnert, C. Sisowath, L. Osório, H. Darban, B. Andersson, A. Kaneko, G. Conseil, A. Björkman, and J. P. Gil. Plasmodium falciparum multidrug resistance protein 1 and artemisinin-based combination therapy in Africa. *The Journal of infectious diseases*, 200(9):1456–64, 2009.

[57] R. Daniels, S. K. Volkman, D. A. Milner, N. Mahesh, D. E. Neafsey, D. J. Park, D. Rosen, E. Angelino, P. C. Sabeti, D. F. Wirth, and R. C. Wiegand. A general SNP-based molecular barcode for Plasmodium falciparum identification and tracking. *Malaria Journal*, 7(223), 2008.

[58] R. Daniels, E. J. Hamilton, K. Durfee, D. Ndiaye, D. F. Wirth, D. L. Hartl, and S. K. Volkman. Methods to Increase the Sensitivity of High Resolution Melting Single Nucleotide Polymorphism Genotyping in Malaria. *Journal of visualized experiments : JoVE*, (105):1–8, 2015.

[59] A. A. Dempster, N. N. Laird, and D. D. B. Rubin. Maximum likelihood from incomplete data via the EM algorithm. *Journal of the Royal Statistical Society Series B Methodological*, 39(1):1–38, 1977.

[60] Division of Parasitic Diseases and Malaria. Laboratory diagnosis of malaria: Plasmodium spp. Life Cycle of Plasmodium spp. *http://www.cdc.gov/dpdx/resources/pdf/benchAids/malaria/Parasitemia_and_LifeCycle.pdf*, 2013.

[61] A. Djimdé, O. K. Doumbo, J. F. Cortese, K. Kayentao, S. Doumbo, Y. Diourte, A. Dicko, X.-Z. Su, T. Nomura, D. A. Fidock, T. E. Wellems, and C. V. Plowe. A molecular marker for chloroquine-resistant falciparum malaria. *New England Journal of Medicine*, 344(4):257–263, 2001.

[62] A. A. Djimde, A. Dolo, A. Ouattara, S. Diakite, C. V. Plowe, and O. K. Doumbo. Molecular diagnosis of resistance to antimalarial drugs during epidemics and in war zones. *The Journal of infectious diseases*, 190(4):853–855, 2004.

[63] C. B. Do and S. Batzoglou. What is the expectation maximization algorithm? *Nature biotechnology*, 26(8):897–899, 2008.

[64] A. M. Dondorp, F. Nosten, P. Yi, D. Das, A. P. Phyo, J. Tarning, K. M. Lwin, F. Ariey, W. Hanpithakpong, S. J. Lee, Others, P. Ringwald, K. Silamut, M. Imwong, K. Chotivanich, P. Lim, T. Herdman, S. S. An, S. Yeung, P. Singhasivanon, N. P. J. Day, N. Lindegardh, D. Socheat, and N. J. White. Artemisinin resistance in Plasmodium falciparum malaria. *The New England journal of medicine*, 361(5):455–67, 2009.

[65] A. M. Dondorp, R. M. Fairhurst, L. Slutsker, J. R. MacArthur, J. G. Breman, P. J. Guerin, T. E. Wellems, P. Ringwald, R. D. Newman, and C. V. Plowe. The threat of artemisinin-resistant malaria. *New England Journal of Medicine*, 365(12):1073–1075, 2011.

[66] P. Druilhe, P. Daubersies, J. Patarapotikul, C. Gentil, L. Chene, T. Chongsuphajaisiddhi, S. Mellouk, and G. Langsley. A primary malarial infection is composed of a very wide range of genetically diverse but related parasites. *Journal of Clinical Investigation*, 101(9):2008–2016, 1998.

[67] S. Duane, A. Kennedy, B. J. Pendleton, and D. Roweth. Hybrid Monte Carlo. *Physics Letters B*, 195(2):216–222, 1987.

[68] R. T. Eastman, N. V. Dharia, E. A. Winzeler, and D. A. Fidock. Piperaquine resistance is associated with a copy number variation on chromosome 5 in drug-pressured Plasmodium falciparum parasites. *Antimicrobial Agents and Chemotherapy*, 55(8):3908–3916, 2011.

[69] A. Ecker, A. M. Lehane, J. Clain, and D. A. Fidock. PfCRT and its role in antimalarial drug resistance. *Trends in parasitology*, 28(11):504–514, 2012.

[70] A. A. Escalante, H. M. Grebert, S. C. Chaiyaroj, M. Magris, S. Biswas, B. L. Nahlen, and A. A. Lal. Polymorphism in the gene encoding the apical membrane antigen-1 (AMA-1) of Plasmodium falciparum. X. Asembo Bay Cohort Project. *Molecular and biochemical parasitology*, 113(2):279–87, 2001.

[71] C. A. Espinal, L. M. Uribe, A. Eslava, and M. E. Rodriguez. Resistencia del Plasmodium falciparum a la combinacion sulfa-primetamina. *Biomedica*, 1(4), 1981.

[72] Europe PMC Funders Group. Mitigating the threat of artemisinin resistance in Africa: improvement of drug-resistance surveillance and response systems. *Lancet Infectious Diseases*, 12(11):888–896, 2013.

[73] European Medicines Agency. First malaria vaccine receives positive scientific opinion from EMA. *Press release*, 2015.

[74] L. Excoffier and M. Slatkin. Maximum-likelihood estimation of molecular haplotype frequencies in a diploid population. *Mol. Biol. Evol.*, 12(5):921–927, 1995.

[75] N. Falk, N. Maire, W. Sama, S. Owusu-Agyei, T. Smith, H.-P. Beck, and I. Felger. Comparison of PCR-RFLP and Genescan-based genotyping for analyzing infection dynamics of Plasmodium falciparum. *The American journal of tropical medicine and hygiene*, 74(6):944–50, 2006.

[76] D. R. V. Färnert A, Arez AP, Babiker HA, Beck HP, Benito A, Björkman A, Bruce MC, Conway DJ, Day KP, Henning L, Mercereau-Puijalon O, Ranford-Cartwright LC, Rubio JM, Snounou G, Walliker D, Zwetyenga J. Genotyping multicentre of Plasmodium study

falciparum infections by PCR: a comparative multicentre study. *Transactions of the Royal Society of Tropical Medicine & Hygiene*, 95:225–232, 2001.

[77] A. Farnert, G. Snounou, I. Rooth, and A. Bjorkman. Daily dynamics of Plasmodium falciparum subpopulations in asymptomatic children in a holoendemic area. *American Journal of Tropical Medicine and Hygiene*, 56(5):538–547, 1997.

[78] I. Felger and H.-P. Beck. Genotyping of Plasmodium falciparum. PCR-RFLP analysis. *Methods in Molecular Medicin*, 72:117–129, 2002.

[79] I. Felger, T. Smith, D. Edoh, A. Kitua, P. Alonso, M. Tanner, and H. P. Beck. Multiple plasmodium falciparum infections in Tanzanian infants. *Transactions of the Royal Society of Tropical Medicine and Hygiene*, 93(suppl 1):29–34, 1999.

[80] I. Felger, A. Irion, S. Steiger, and H. P. Beck. Epidemiology of multiple Plasmodium falciparum infections. *Transactions of the Royal Society of Tropical Medicine & Hygiene*, 100, 1999.

[81] I. Felger, M. Maire, M. T. Bretscher, N. Falk, A. Tiaden, W. Sama, H.-P. P. Beck, S. Owusu-Agyei, and T. A. Smith. The Dynamics of Natural Plasmodium falciparum Infections. *PLoS ONE*, 7(9):e45542, 2012.

[82] D. A. Fidock, T. Nomura, A. K. Talley, R. A. Cooper, S. M. Dzekunov, M. T. Ferdig, L. M. Ursos, A. B. Sidhu, B. Naudé, K. W. Deitsch, X. Z. Su, J. C. Wootton, P. D. Roepe, and T. E. Wellems. Mutations in the P. falciparum digestive vacuole transmembrane protein PfCRT and evidence for their role in chloroquine resistance. *Molecular cell*, 6(4):861–71, 2000.

[83] D. Francis, S. L. Nsobya, A. Talisuna, A. Yeka, M. R. Kamya, R. Machekano, C. Dokomajilar, P. J. Rosenthal, and G. Dorsey. Geographic differences in antimalarial drug efficacy in Uganda are explained by differences in endemicity and not by known molecular markers of drug resistance. *The Journal of infectious diseases*, 193(7):978–86, 2006.

[84] K. Galinsky, C. Valim, A. Salmier, B. de Thoisy, L. Musset, E. Legrand, A. Faust, M. Baniecki, D. Ndiaye, R. F. Daniels, D. L. Hartl, P. C. Sabeti, D. F. Wirth, S. K. Volkman, and D. E. Neafsey. COIL: a methodology for evaluating malarial complexity of infection using likelihood from single nucleotide polymorphism data. *Malaria Journal*, 14(1):4, 2015.

[85] M. Gardner, N. Hall, E. Fung, O. White, M. Berriman, R. Hyman, J. Carlton, A. Pain, K. Nelson, S. Bowman, I. Paulsen, K. James, J. Eisen, K. Rutherford, S. Salzberg, A. Craig, S. Kyes, M. Chan, V. Nene, S. J. Shallom, B. Suh, J. Peterson, S. Angiuoli, M. Pertea, J. Allen, J. Selengut, D. Haft, M. W. Mather, A. B. Vaidya, D. M. A. Martin, A. H. Fairlamb, M. J. Fraunholz, D. S. Roos, S. A. Ralph, G. I. McFadden, L. M. Cummings, G. M. Subramanian, C. Mungall, J. C. Venter, D. J. Carucci, S. L. Hoffman, C. Newbold, R. W. Davis, C. M. Fraser, and B. Barrell. Genome sequence of the human malaria parasite Plasmodium falciparum. *Nature*, 419:498–511, 2002.

[86] W. Gatei, S. Kariuki, W. Hawley, F. ter Kuile, D. Terlouw, P. Phillips-Howard, B. Nahlen, J. Gimnig, K. Lindblade, E. Walker, M. Hamel, S. Crawford, J. Williamson, L. Slutsker, and Y. P. Shi. Effects of transmission reduction by insecticide-treated bed nets (ITNs) on parasite genetics population structure: I. The genetic diversity of Plasmodium falciparum parasites by microsatellite markers in western Kenya. *Malaria Journal*, 9(1):353, 2010.

[87] A. Gelman, A. Jakulin, M. G. Pittau, and Y.-S. Su. A weakly informative default prior distribution for logistic and other regression models. *The Annals of Applied Statistics*, 2(4):1360–1383, 2008.

[88] A. Gelman, J. B. Carlin, H. S. Stern, and D. B. Rubin. *Bayesian Data Analysis*. Chapman & Hall/CRC Press, second edition, 2009.

[89] P. I. German and F. T. Aweeka. Clinical pharmacology of artemisinin-based combination therapies. *Clinical pharmacokinetics*, 47(2):91–102, 2008.

[90] W. R. Gilks, S. Richardson, and D. J. Spiegelhalter, editors. *Markov Chain Monte Carlo in Practice*. Chapman & Hall, first edition, 1996.

[91] A. Guerra-Neira, J. M. Rubio, J. R. Royo, J. C. Ortega, A. S. Auñón, P. B. Diaz, and A. B. Llanes. Plasmodium diversity in non-malaria individuals from the Bioko Island in Equatorial Guinea (West Central-Africa). *International journal of health geographics*, 5:27, 2006.

[92] V. Gupta, G. Dorsey, A. E. Hubbard, P. J. Rosenthal, and B. Greenhouse. Gel versus capillary electrophoresis genotyping for categorizing treatment outcomes in two anti-malarial trials in Uganda. *Malaria Journal*, 9:19, 2010.

[93] H. Haario, E. Saksman, and J. Tamminen. An adaptive Metropolis algorithm. *Bernoulli*, 7(2):223–242, 2001.

[94] J. A. Hasler, I. Johansson, and C. M. Masimirembwa. Inhibitory effects of antiparasitic drugs on cytochrome P450 2D6. *European journal of clinical pharmacology*, 48(1):35–38, 1995.

[95] I. M. Hastings and T. A. Smith. MalHaploFreq: A computer programme for estimating malaria haplotype frequencies from blood samples. *Malaria Journal*, 7(130), 2008.

[96] I. M. Hastings, C. Nsanzabana, and T. A. Smith. A comparison of methods to detect and quantify the markers of antimalarial drug resistance. *The American journal of tropical medicine and hygiene*, 83(3):489–95, 2010.

[97] I. M. Hastings. Malaria control and the evolution of drug resistance: an intriguing link. *Trends in parasitology*, 19(2):70–73, 2003.

[98] T. Havryliuk, P. Orjuela-Sánchez, and M. U. Ferreira. Plasmodium vivax: microsatellite analysis of multiple-clone infections. *Experimental parasitology*, 120(4):330–6, 2008.

[99] S. I. Hay, D. J. Rogers, J. F. Toomer, and R. W. Snow. Annual Plasmodium falciparum entomological inoculation rates (EIR) across Africa: literature survey, Internet access and review. *Transactions of the Royal Society of Tropical Medicine and Hygiene*, 94(2):113–127, 2011.

[100] U. Hess, P. M. Timmermans, and M. Jones. Combined chloroquine/Fansidar-resistant falciparum malaria appears in East Africa. *American Journal of Tropical Medicine and Hygiene*, 32(2):217–220, 1983.

[101] T. T. Hien, N. T. Thuy-Nhien, N. H. Phu, M. F. Boni, N. V. Thanh, N. T. Nha-Ca, L. H. Thai, C. Q. Thai, P. V. Toi, P. D. Thuan, L. T. Long, L. T. Dong, L. Merson, C. Dolecek, K. Stepniewska, P. Ringwald, N. J. White, J. Farrar, and M. Wolbers. In vivo susceptibility of Plasmodium falciparum to artesunate in Binh Phuoc Province, Vietnam. *Malaria Journal*, 11(355), 2012.

[102] W. G. Hill and H. A. Babiker. Estimation of numbers of malaria clones in blood samples. *Proceedings of the Royal Society of London B: Biological sciences*, 262(1365):249–57, 1995.

[103] M. Hoffman and A. Gelman. The no-U-turn sampler: Adaptively setting path lengths in Hamiltonian Monte Carlo. *Journal of Machine Learning Research*, 15(2008):1–31, 2014.

[104] S. L. Hoffman, J. Vekemans, T. L. Richie, and P. E. Duffy. The March Toward Malaria Vaccines. *American Journal of Preventive Medicine*, 49(6):S319–S333, 2015.

[105] G. Holmgren, J. P. Gil, P. M. Ferreira, M. I. Veiga, C. O. Obonyo, and A. Björkman. Amodiaquine resistant Plasmodium falciparum malaria in vivo is associated with selection of pfcrt 76T and pfmdr1 86Y. *Infection, Genetics and Evolution*, 6(4):309–314, 2006.

[106] C. Hopkins Sibley, P. J. Guerin, and R. Pascal. Monitoring antimalarial resistance: launching a cooperative effort. *Trends in parasitology*, 26(5):221–224, 2010.

[107] F. Huang, S. Takala-Harrison, C. G. Jacob, H. Liu, X. Sun, H. Yang, M. M. Nyunt, M. Adams, S. Zhou, Z. Xia, P. Ringwald, M. D. Bustos, L. Tang, and C. V. Plower. A single mutation in K13 predominates in Southern China and is associated with delayed clearance of Plasmodium falciparum following artemisinin treatment. *Journal of Infectious Diseases*, 2015.

[108] E. S. Hurwitz, D. Johnson, and C. C. Campbell. Resistance of Plasmodium falciparum malaria to sulfadoxine-pyrimethamine ('Fansidar') in a refugee camp in Thailand. *The Lancet*, 317(8229):1068–1070, 1981.

[109] A. Iliadis, D. Anastassiou, and X. Wang. A sequential Monte Carlo framework for haplotype inference in CNV/SNP genotype data. *EURASIP journal on bioinformatics & systems biology*, 2014(1):7, 2014.

[110] Illumina. An Introduction to Next-Generation Sequencing Technology Table of Contents. Technical Report (Illumina, 2015.

[111] M. Imwong, S. Hanchana, B. Malleret, L. Rénia, N. P. J. Day, A. Dondorp, F. Nosten, G. Snounou, and N. J. White. High throughput ultra-sensitive molecular techniques to quantify low density malaria parasitaemias. *Journal of clinical microbiology*, 52(9):3303–3309, 2014.

[112] H. A. Ismail, U. Ribacke, L. Reiling, J. Normark, T. Egwang, F. Kironde, J. G. Beeson, M. Wahlgren, and K. E. M. Persson. Acquired antibodies to merozoite antigens in children from Uganda with uncomplicated or severe plasmodium falciparum malaria. *Clinical and Vaccine Immunology*, 20(8):1170–1180, 2013.

[113] J. J. Juliano, J. J. Kwiek, K. Cappell, V. Mwapasa, and S. R. Meshnick. Minority-variant pfcrt K76T mutations and chloroquine resistance, Malawi. *Emerging Infectious Diseases*, 13(6):872–827, 2007.

[114] J. J. Juliano, P. Trottman, V. Mwapasa, and R. S. Meshnick. Short Report: Detection of the Dihydrofolate Reductase–164L Mutation in Plasmodium falciparum Infections from Malawi by Heteroduplex Tracking Assay Jonathan. *American Journal of Tropical Medicine and Hygiene*, 78(6):892–894, 2008.

[115] J. J. Juliano, M. Randrianarivelojosia, B. Ramarosandratana, F. Ariey, V. Mwapasa, and S. R. Meshnick. Nonradioactive heteroduplex tracking assay for the detection of minority-variant chloroquine-resistant Plasmodium falciparum in Madagascar. *Malaria Journal*, 8(47), 2009.

[116] J. Kapisi, V. Bigira, T. Clark, S. Kinara, F. Mwangwa, J. Achan, M. Kamya, S. Soremekun, and G. Dorsey. Efficacy and safety of artemether-lumefantrine for the treatment of uncomplicated malaria in the setting of three different chemopreventive regimens. *Malaria Journal*, 14(1):53, 2015.

[117] D. Kessner, T. L. Turner, and J. Novembre. Maximum likelihood estimation of frequencies of known haplotypes from pooled sequence data. *Molecular biology and evolution*, 30(5):1145–58, 2013.

[118] W. C. Kiarie, L. Wangai, E. Agola, F. T. Kimani, and C. Hungu. Chloroquine sensitivity: diminished prevalence of chloroquine-resistant gene marker pfcrt-76 13 years after cessation of chloroquine use in Msambweni, Kenya. *Malaria Journal*, 14(328), 2015.

[119] M. S. Kiwuwa, U. Ribacke, K. Moll, J. Byarugaba, K. Lundblom, A. Färnert, K. Fred, and M. Wahlgren. Genetic diversity of Plasmodium falciparum infections in mild and severe malaria of children from Kampala, Uganda. *Parasitology Research*, 112(4):1691–1700, 2013.

[120] C. Koepfli, S. Schoepflin, M. Bretscher, E. Lin, B. Kiniboro, P. A. Zimmerman, P. Siba, T. A. Smith, I. Mueller, and I. Felger. How much remains undetected? Probability of molecular detection of human Plasmodia in the field. *PloS one*, 6(4):e19010, 2011.

[121] L. Konate, J. Zwetyenga, C. Rogier, E. Bischoff, D. Fontenille, A. Tall, A. Spiegel, J.-F. Trape, and O. Mercereau-Puijalon. Variation of Plasmodium falciparum mspl block 2 and msp2 allele prevalence and of infection complexity in two neighbouring Senegalese villages with different transmission conditions. *Transactions of the Royal Society of Tropical Medicine and Hygiene*, 93(suppl 1):S1/21–S1/28, 1999.

[122] D. J. Krogstad. Malaria as a Reemerging Disease. *Epidemiologic Reviews*, 18(1):77–89, 1996.

[123] J. G. Kublin, F. K. Dzinjalamala, D. D. Kamwendo, E. M. Malkin, J. F. Cortese, L. M. Martino, R. A. G. Mukadam, S. J. Rogerson, A. G. Lescano, M. E. Molyneux, P. A. Winstanley, P. Chimpeni, T. E. Taylor, and C. V. Plowe. Molecular markers for failure of sulfadoxine-pyrimethamine and chlorproguanil-dapsone treatment of Plasmodium falciparum malaria. *The Journal of infectious diseases*, 185(3):380–388, 2002.

[124] J. G. Kublin, J. F. Cortese, E. M. Njunju, R. A. G. Mukadam, J. J. Wirima, P. N. Kazembe, A. A. Djimdé, B. Kouriba, T. E. Taylor, and C. V. Plowe. Reemergence of chloroquine-sensitive Plasmodium falciparum malaria after cessation of chloroquine use in Malawi. *The Journal of infectious diseases*, 187(12):1870–5, 2003.

[125] C. K. Kum, D. Thorburn, G. Ghilagaber, P. Gil, and A. Björkman. On the effects of malaria treatment on parasite drug resistance–probability modelling of genotyped malaria infections. *The international journal of biostatistics*, 9(1):135–148, 2013.

[126] M. P. Kyaw, M. H. Nyunt, K. Chit, M. M. Aye, K. H. Aye, N. Lindegardh, J. Tarning, M. Imwong, C. G. Jacob, C. Rasmussen, J. Perin, P. Ringwald, and M. M. Nyunt. Reduced Susceptibility of Plasmodium falciparum to Artesunate in Southern Myanmar. *PloS one*, 8(3):e57689, 2013.

[127] T. Laver, J. Harrison, P. A. O'Neill, K. Moore, A. Farbos, K. Paszkiewicz, and D. J. Studholme. Assessing the performance of the Oxford Nanopore Technologies MinION. *Biomolecular Detection and Quantification*, 3:1–8, 2015.

[128] M. Lawrence, W. Huber, H. Pages, P. Aboyoun, M. Carlson, R. Gentleman, M. T. Morgan, and V. J. Carey. Software for Computing and Annotating Genomic Ranges. *PLoS Computational Biology*, 9(8):1–10, 2013.

[129] X. Li, A. S. Foulkes, R. M. Yucel, and S. M. Rich. An expectation maximization approach to estimate malaria haplotype frequencies in multiply infected children. *Statistical applications in genetics and molecular biology*, 6(1), 2007.

[130] X. Li, B. N. Thomas, S. M. Rich, D. Ecker, J. K. Tumwine, and A. S. Foulkes. Estimating and testing haplotype-trait associations in non-diploid populations. *Journal of the Royal Statistical Society. Series C, Applied statistics*, 58(5):663–678, 2009.

[131] X. Li. EM Estimation of Malaria Haplotype Probabilities from Multiply Infected Human Blood Samples. *https://cran.r-project.org/src/contrib/Archive/malaria.em/*, R package, 2012.

[132] J. Little, Roderick and B. Rubin, Donald. *Statistical Analysis with Missing Data*. John Wiley & Sons, second edition, 2014.

[133] S. Liu, J. Mu, H. Jiang, and X.-z. Su. Effects of Plasmodium falciparum mixed infections on in vitro antimalarial drug tests and genotyping. *The American journal of tropical medicine and hygiene*, 79(2):178–84, 2008.

[134] Q. Long, D. C. Jeffares, Q. Zhang, K. Ye, V. Nizhynska, Z. Ning, C. Tyler-Smith, and M. Nordborg. PoolHap: inferring haplotype frequencies from pooled samples by next generation sequencing. *PloS one*, 6(1):e15292, 2011.

[135] N. W. Lucchi, F. Komino, S. A. Okoth, I. Goldman, P. Onyona, R. E. Wiegand, E. Juma, Y. P. Shi, J. W. Barnwell, V. Udhayakumar, and S. Kariuki. In vitro and molecular surveillance for antimalarial drug resistance in Plasmodium falciparum parasites in western Kenya reveals sustained artemisinin sensitivity and increased chloroquine sensitivity. *Antimicrobial Agents and Chemotherapy*, 59(12):7540–7547, 2015.

[136] D. Lunn, J. Barrett, M. Sweeting, and S. Thompson. Fully Bayesian hierarchical modelling in two stages, with application to meta-analysis. *Journal of the Royal Statistical Society: Series C (Applied statistics)*, 62(4):551–572, 2013.

[137] G. Macdonald. The analysis of infection rates in diseases in which superinfection occurs. *Tropical diseases bulletin*, 47(10):907–15, 1950.

[138] O. Maïga-Ascofaré, J. Le Bras, R. Mazmouz, E. Renard, S. Falcão, E. Broussier, D. Bustos, M. Randrianarivelojosia, S. A. Omar, A. Aubouy, J.-F. Lepère, V. Jean-François, A. A. Djimdé, and J. Clain. Adaptive differentiation of Plasmodium falciparum populations inferred from single-nucleotide polymorphisms (SNPs) conferring drug resistance and from neutral SNPs. *The Journal of infectious diseases*, 202(7):1095–1103, 2010.

[139] Malaria Control Programme Ministry of Health. Uganda Malaria Control Strategic Plan. Technical report, 2005.

[140] A. Malisa, R. Pearce, B. Mutayoba, S. Abdullah, H. Mshinda, P. Kachur, P. Bloland, and C. Roper. Quantification of markers of antimalarial drug resistance from an area of high malaria transmission: Comparing frequency with prevalence. *African Journal of Biotechnology*, 11(69):13250–13260, 2012.

[141] M. Malmberg, B. Ngasala, P. E. Ferreira, E. Larsson, I. Jovel, A. Hjalmarsson, M. Petzold, Z. Premji, J. P. Gil, A. Björkman, and A. Mårtensson. Temporal trends of molecular markers associated with artemether-lumefantrine tolerance/resistance in Bagamoyo district, Tanzania. *Malaria Journal*, 12(1):103, 2013.

[142] M. Manske, O. Miotto, S. Campino, S. Auburn, J. Almagro-Garcia, G. Maslen, J. O'Brien, A. Djimde, O. Doumbo, I. Zongo, J.-B. Ouedraogo, P. Michon, I. Mueller, P. Siba, A. Nzila, S. Borrmann, S. M. Kiara, K. Marsh, H. Jiang, X.-Z. Su, C. Amaratunga, R. Fairhurst, D. Socheat, F. Nosten, M. Imwong, N. J. White, M. Sanders, E. Anastasi, D. Alcock, E. Drury, S. Oyola, M. A. Quail, D. J. Turner, V. Ruano-Rubio, D. Jyothi, L. Amenga-Etego, C. Hubbart, A. Jeffreys, K. Rowlands, C. Sutherland, C. Roper, V. Mangano, D. Modiano, J. C. Tan, M. T. Ferdig, A. Amambua-Ngwa, D. J. Conway, S. Takala-Harrison, C. V. Plowe, J. C. Rayner, K. A. Rockett, T. G. Clark, C. I. Newbold, M. Berriman, B. MacInnis, and D. P. Kwiatkowski. Analysis of Plasmodium falciparum diversity in natural infections by deep sequencing. *Nature*, 487:375–379, 2012.

[143] E. R. Mardis. Next-Generation DNA Sequencing Methods. *Annual Review of Genomics and Human Genetics*, 9(1):387–402, 2008.

[144] J. Marfurt, T. A. Smith, I. M. Hastings, I. Müller, A. Sie, O. Oa, M. Baisor, J. C. Reeder, H.-P. Beck, and B. Genton. Plasmodium falciparum resistance to anti-malarial drugs in Papua New Guinea: evaluation of a community-based approach for the molecular monitoring of resistance. *Malaria Journal*, 9:8, 2010.

[145] J. Marfurt. *Drug resistant malaria in Papua New Guinea and molecular monitoring of parasite resistance*. PhD thesis, 2006.

[146] E. Marinari and G. Parisi. Simulated Tempering: A New Monte Carlo Scheme. *EPL (Europhysics Letters)*, 19(6):451–458, 1992.

[147] K. Marsh. Malaria disaster in Africa. *The Lancet*, 352(9132):924, 1998.

[148] R. J. Maude, W. Pontavornpinyo, S. Saralamba, R. Aguas, S. Yeung, A. M. Dondorp, N. P. J. Day, N. J. White, and L. J. White. The last man standing is the most resistant: eliminating artemisinin-resistant malaria in Cambodia. *Malaria Journal*, 8:31, 2009.

[149] G. W. Mbogo, S. Nankoberanyi, S. Tukwasibwe, F. N. Baliraine, S. L. Nsobya, M. D. Conrad, E. Arinaitwe, M. Kamya, J. Tappero, S. G. Staedke, G. Dorsey, B. Greenhouse, and P. J. Rosenthal. Temporal Changes in Prevalence of Molecular Markers Mediating Antimalarial Drug Resistance in a High Malaria Transmission Setting in Uganda. *The American journal of tropical medicine and hygiene*, 2014.

[150] D. Ménard, N. Khim, J. Beghain, A. A. Adegnika, M. Shafiul-Alam, O. Amodu, G. Rahim-Awab, C. Barnadas, A. Berry, Y. Boum, M. D. Bustos, J. Cao, J.-H. Chen, L. Collet, L. Cui, G.-D. Thakur, A. Dieye, D. Djallé, M. A. Dorkenoo, C. E. Eboumbou-Moukoko, F.-E.-C. J. Espino, T. Fandeur, M.-F. Ferreira-da Cruz, A. A. Fola, H.-P. Fuehrer, A. M. Hassan, S. Herrera, B. Hongvanthong, S. Houzé, M. L. Ibrahim, M. Jahirul-Karim, L. Jiang, S. Kano, W. Ali-Khan, M. Khanthavong, P. G. Kremsner, M. Lacerda, R. Leang, M. Leelawong, M. Li, K. Lin, J.-B. Mazarati, S. Ménard, I. Morlais, H. Muhindo-Mavoko, L. Musset, K. Na-Bangchang, M. Nambozi, K. Niaré, H. Noedl, J.-B. Ouédraogo, D. R. Pillai, B. Pradines, B. Quang-Phuc, M. Ramharter, M. Randrianarivelojosia, J. Sattabongkot, A. Sheikh-Omar, K. D. Silué, S. B. Sirima, C. Sutherland, D. Syafruddin, R. Tahar, L.-H. Tang, O. A. Touré, P. Tshibangu-wa Tshibangu, I. Vigan-Womas, M. Warsame, L. Wini, S. Zakeri, S. Kim, R. Eam, L. Berne, C. Khean, S. Chy, M. Ken, K. Loch, L. Canier, V. Duru, E. Legrand, J.-C. Barale, B. Stokes, J. Straimer, B. Witkowski, D. A. Fidock, C. Rogier, P. Ringwald, F. Ariey, and O. Mercereau-Puijalon. A Worldwide Map of Plasmodium falciparum K13-Propeller Polymorphisms. *The New England journal of medicine*, 374(25):2453–2464, 2016.

[151] M. L. Metzker. Sequencing technologies - the next generation. *Nature reviews. Genetics*, 11(1):31–46, 2010.

[152] L. H. Miller. Distribution of mature trophozoites and schizonts of Plasmodium falciparum in the organs of Aotus trivirgatus, the night monkey. *American Journal of Tropical Medicine and Hygiene*, 18(6):860–865, 1969.

[153] O. Miotto, J. Almagro-Garcia, M. Manske, B. Macinnis, S. Campino, K. A. Rockett, C. Amaratunga, P. Lim, S. Suon, S. Sreng, J. M. Anderson, S. Duong, C. Nguon, C. M. Chuor, D. Saunders, Y. Se, C. Lon, M. M. Fukuda, L. Amenga-Etego, A. V. O. Hodgson, V. Asoala, M. Imwong, S. Takala-Harrison, F. Nosten, X.-Z. Su, P. Ringwald, F. Ariey, C. Dolecek, T. T. Hien, M. F. Boni, C. Q. Thai, A. Amambua-Ngwa, D. J. Conway, A. A. Djimdé, O. K. Doumbo, I. Zongo, J.-B. Ouedraogo, D. Alcock, E. Drury, S. Auburn, O. Koch, M. Sanders, C. Hubbart, G. Maslen, V. Ruano-Rubio, D. Jyothi, A. Miles, J. O'Brien, C. Gamble, S. O. Oyola, J. C. Rayner, C. I. Newbold, M. Berriman, C. C. A. Spencer, G. McVean, N. P. Day, N. J. White, D. Bethell, A. M. Dondorp, C. V. Plowe, R. M. Fairhurst, and D. P. Kwiatkowski. Multiple populations of artemisinin-resistant Plasmodium falciparum in Cambodia. *Nature Genetics*, 45(April):648–55, 2013.

[154] O. Miotto, R. Amato, E. A. Ashley, B. MacInnis, J. Almagro-Garcia, C. Amaratunga, P. Lim, D. Mead, S. O. Oyola, M. Dhorda, M. Imwong, C. Woodrow, M. Manske, J. Stalker,

E. Drury, S. Campino, L. Amenga-Etego, T.-N. N. Thanh, H. T. Tran, P. Ringwald, D. Bethell, F. Nosten, A. P. Phyo, S. Pukrittayakamee, K. Chotivanich, C. M. Chuor, C. Nguon, S. Suon, S. Sreng, P. N. Newton, M. Mayxay, M. Khanthavong, B. Hongvanthong, Y. Htut, K. T. Han, M. P. Kyaw, M. A. Faiz, C. I. Fanello, M. Onyamboko, O. A. Mokuolu, C. G. Jacob, S. Takala-Harrison, C. V. Plowe, N. P. Day, A. M. Dondorp, C. C. A. Spencer, G. McVean, R. M. Fairhurst, N. J. White, and D. P. Kwiatkowski. Genetic architecture of artemisinin-resistant Plasmodium falciparum. *Nature genetics*, 47(3):226–34, 2015.

[155] T. Mita, A. Kaneko, J. K. Lum, B. Bwijo, M. Takechi, I. L. Zungu, T. Tsukahara, K. Tanabe, T. Kobayakawa, and A. Björkman. Recovery of chloroquine sensitivity and low prevalence of the Plasmodium falciparum chloroquine resistance transporter gene mutation K76T following the discontinuance of chloroquine use in Malawi. *American Journal of Tropical Medicine and Hygiene*, 68(4):413–415, 2003.

[156] T. Mita, M. Venkatesan, J. Ohashi, R. Culleton, N. Takahashi, T. Tsukahara, M. Ndounga, L. Dysoley, H. Endo, F. Hombhanje, M. U. Ferreira, C. V. Plowe, and K. Tanabe. Limited geographical origin and global spread of sulfadoxine-resistant dhps alleles in plasmodium falciparum populations. *Journal of Infectious Diseases*, 204(12):1980–1988, 2011.

[157] F. P. Mockenhaupt, S. Ehrhardt, R. Otchwemah, T. A. Eggelte, S. D. Anemana, K. Stark, U. Bienzle, and E. Kohne. Limited influence of haemoglobin variants on Plasmodium falciparum msp1 and msp2 alleles in symptomatic malaria. *Transactions of the Royal Society of Tropical Medicine and Hygiene*, 98(5):302–310, 2004.

[158] A. Mohammed, A. Ndaro, A. Kalinga, A. Manjurano, J. F. Mosha, D. F. Mosha, M. van Zwetselaar, J. B. Koenderink, F. W. Mosha, M. Alifrangis, H. Reyburn, C. Roper, and R. A. Kavishe. Trends in chloroquine resistance marker, Pfcrt-K76T mutation ten years after chloroquine withdrawal in Tanzania. *Malaria Journal*, 12:415, 2013.

[159] J. F. Monahan. *Numerical methods of statistics*. Cambridge University Press, 2011.

[160] M. Morgan, H. Pagès, V. Obenchain, and N. Hayden. Rsamtools: Binary alignment (BAM), FASTA, variant call (BCF), and tabix file import. *http://bioconductor.org/packages/release/bioc/html/Rsamtools.html*.

[161] S. Muangnoicharoen, D. J. Johnson, S. Looareesuwan, S. Krudsood, and S. A. Ward. Role of known molecular markers of resistance in the antimalarial potency of piperaquine and dihydroartemisinin in vitro. *Antimicrobial Agents and Chemotherapy*, 53(4):1362–1366, 2009.

[162] L. Mwai, S. M. Kiara, A. Abdirahman, L. Pole, A. Rippert, A. Diriye, P. Bull, K. Marsh, S. Borrmann, and A. Nzila. In Vitro Activities of Piperaquine, Lumefantrine, and Dihydroartemisinin in Kenyan Plasmodium falciparum Isolates and Polymorphisms in pfcrt and pfmdr1. *Antimicrobial agents and chemotherapy*, 53(12):5069–5073, 2009.

[163] L. Mwai, A. Diriye, V. Masseno, S. Muriithi, T. Feltwell, J. Musyoki, J. Lemieux, A. Feller, G. R. Mair, K. Marsh, C. Newbold, A. Nzila, and C. K. Carret. Genome Wide Adaptations of Plasmodium falciparum in Response to Lumefantrine Selective Drug Pressure. *PloS one*, 7(2), 2012.

[164] F. Mwingira, G. Nkwengulila, S. Schoepflin, D. Sumari, H.-P. Beck, G. Snounou, I. Felger, P. Olliaro, and K. Mugittu. Plasmodium falciparum msp1, msp2 and glurp allele frequency and diversity in sub-Saharan Africa. *Malaria Journal*, 10(79), 2011.

[165] D. N. Nabarro and E. M. Tayler. The "Roll Back Malaria" Campaign. *Science*, 280(5372):2067–2068, 1998.

[166] I. Naidoo and C. Roper. Following the path of most resistance: dhps K540E dispersal in African Plasmodium falciparum. *Trends in parasitology*, 26(9):447–456, 2010.

[167] I. Naidoo and C. Roper. Drug resistance maps to guide intermittent preventive treatment of malaria in African infants. *Parasitology*, 138:1469–1479, 2011.

[168] I. Naidoo and C. Roper. Mapping 'partially resistant', 'fully resistant', and 'super resistant' malaria. *Trends in parasitology*, 29(10):505–15, 2013.

[169] S. Nair, B. Miller, M. Barends, A. Jaidee, J. Patel, M. Mayxay, P. Newton, F. Nosten, M. T. Ferdig, and T. J. C. Anderson. Adaptive copy number evolution in malaria parasites. *PLoS Genetics*, 4(10), 2008.

[170] S. Nair, S. C. Nkhoma, D. Serre, P. A. Zimmerman, K. Gorena, B. J. Daniel, F. Nosten, T. J. C. Anderson, and I. H. Cheeseman. Single-cell genomics for dissection of complex malaria infections. *Genome Research*, 24(6):1028–1038, 2014.

[171] J. A. Nájera, M. González-Silva, and P. L. Alonso. Some lessons for the future from the Global Malaria Eradication Programme (1955-1969). *PLoS medicine*, 8(1):e1000412, 2011.

[172] M. Nanyunja, J. Nabyonga Orem, F. Kato, M. Kaggwa, C. Katureebe, and J. Saweka. Malaria Treatment Policy Change and Implementation: The Case of Uganda. *Malaria Research and Treatment*, 2011:1–14, 2011.

[173] I. Nasell. *Hybrid Models of Tropical Infections*. Springer, first edition, 1985.

[174] I. Nåsell. On superinfection in malaria. *IMA journal of mathematics applied in medicine and biology*, 3(3):211–27, 1986.

[175] T. C. Nchinda. Malaria: A reemerging disease in Africa. *Emerging Infectious Diseases*, 4(3):398–403, 1998.

[176] M. Ndiaye, B. Faye, R. Tine, J. L. Ndiaye, A. Lo, A. Abiola, Y. Dieng, D. Ndiaye, R. Hallett, M. Alifrangis, and O. Gaye. Assessment of the molecular marker of Plasmodium falciparum chloroquine resistance (Pfcrt) in Senegal after several years of chloroquine withdrawal. *American Journal of Tropical Medicine and Hygiene*, 87(4):640–645, 2012.

[177] D. E. Neafsey, M. Juraska, T. Bedford, D. Benkeser, C. Valim, A. Griggs, M. Lievens, S. Abdulla, S. Adjei, T. Agbenyega, S. T. Agnandji, P. Aide, S. Anderson, D. Ansong, J. J. Aponte, K. P. Asante, P. Bejon, A. J. Birkett, M. Bruls, K. M. Connolly, U. D'Alessandro, C. Dobaño, S. Gesase, B. Greenwood, J. Grimsby, H. Tinto, M. J. Hamel, I. Hoffman, P. Kamthunzi, S. Kariuki, P. G. Kremsner, A. Leach, B. Lell, N. J. Lennon, J. Lusingu, K. Marsh, F. Martinson, J. T. Molel, E. L. Moss, P. Njuguna, C. F. Ockenhouse, B. R. Ogutu, W. Otieno, L. Otieno, K. Otieno, S. Owusu-Agyei, D. J. Park, K. Pellé, D. Robbins, C. Russ, E. M. Ryan, J. Sacarlal, B. Sogoloff, H. Sorgho, M. Tanner, T. Theander, I. Valea, S. K.

Volkman, Q. Yu, D. Lapierre, B. W. Birren, P. B. Gilbert, and D. F. Wirth. Genetic Diversity and Protective Efficacy of the RTS,S/AS01 Malaria Vaccine. *The New England journal of medicine*, 373(21):2025–37, 2015.

[178] J. Nedelman. Estimation for a model of multiple malaria infections. *Biometrics*, 41(2):447–53, 1985.

[179] J. A. Nelder and R. Mead. A simplex method for function minimization. *The Computer Journal*, 7(4):308–313, 1965.

[180] S. C. Nkhoma, S. Nair, I. H. Cheeseman, C. Rohr-Allegrini, S. Singlam, F. Nosten, and T. J. C. Anderson. Close kinship within multiple-genotype malaria parasite infections. *Proceedings of the Royal Society of London B: Biological Sciences*, 279(1738):2589–98, 2012.

[181] H. Noedl, Y. Se, K. Schaecher, B. L. Smith, D. Socheat, and M. M. Fukuda. Evidence of artemisinin-resistant malaria in western Cambodia. *The New England journal of medicine*, 359(24):2619–20, 2008.

[182] C. Nsanzabana, I. M. Hastings, J. Marfurt, I. Müller, K. Baea, L. Rare, A. Schapira, I. Felger, B. Betschart, T. A. Smith, H.-P. Beck, and B. Genton. Quantifying the evolution and impact of antimalarial drug resistance: drug use, spread of resistance, and drug failure over a 12-year period in Papua New Guinea. *The Journal of infectious diseases*, 201(3):435–43, 2010.

[183] F. Ntoumi, H. Contamin, C. Rogier, S. Bonnefoy, J. F. Trape, O. Mercereau-Puijalon, and O. Mercereau- Puijalon. Age-dependent carriage of multiple Plasmodium falciparum merozoite surface antigen-2 alleles in asymptomatic malaria infections. *The American journal of tropical medicine and hygiene*, 52(1):81–8, 1995.

[184] D. Nwakanma, A. Kheir, M. Sowa, S. Dunyo, M. Jawara, M. Pinder, P. Milligan, D. Walliker, and H. A. Babiker. High gametocyte complexity and mosquito infectivity of Plasmodium falciparum in the Gambia. *International Journal for Parasitology*, 38(2):219–227, 2008.

[185] J. D. O'Brien, X. Didelot, Z. Iqbal, L. Amenga-Etego, B. Ahiska, and D. Falush. A Bayesian Approach to Inferring the Phylogenetic Structure of Communities from Metagenomic Data. *Genetics*, pages 1–27, 2014.

[186] J. D. O'Brien, Z. Iqbal, and L. Amenga-Etego. An integrative statistical model for inferring strain admixture within clinical Plasmodium falciparum isolates. *arXiv:1505.08171v1*, pages 1–20, 2015.

[187] A. O'Hagan. *Kendall's advanced theory of statistics, volume 2B: Bayesian inference*. Arnold, 1994.

[188] P. Olliaro. Mode of action and mechanisms of resistance for antimalarial drugs. *Pharmacology & therapeutics*, 89(2):207–19, 2001.

[189] S. Owusu-Agyei, T. Smith, H. P. Beck, L. Amenga-Etego, and I. Felger. Molecular epidemiology of Plasmodium falciparum infections among asymptomatic inhabitants of a holoendemic malarious area in northern Ghana. *Tropical Medicine and International Health*, 7(5):421–428, 2002.

[190] H. Pages, P. Aboyoun, R. Gentleman, and S. DebRoy. Biostrings: String objects representing biological sequences, and matching algorithms.

[191] N. Papa Mze, Y. D. Ndiaye, C. K. Diedhiou, S. Rahamatou, B. Dieye, R. F. Daniels, E. J. Hamilton, M. Diallo, A. K. Bei, D. F. Wirth, S. Mboup, S. K. Volkman, A. D. Ahouidi, and D. Ndiaye. RDTs as a source of DNA to study Plasmodium falciparum drug resistance in isolates from Senegal and the Comoros Islands. *Malaria Journal*, 14:373, 2015.

[192] O. Papaspiliopoulos, G. O. Roberts, and M. Sköld. A general framework for the parametrization of hierarchical models. *Statistical Science*, 22(1):59–73, 2007.

[193] D. Payne. Spread of chloroquine resistance in Plasmodium falciparum. *Parasitology Today*, 3(8):241–246, 1987.

[194] D. Payne. Did medicated salt hasten the spread of chloroquine resistance in Plasmodium falciparum? *Parasitology Today*, 4(4):112–115, 1988.

[195] R. J. Pearce, H. Pota, M.-S. Evehe, E.-H. Bâ, G. Mombo-Ngoma, A. L. Malisa, R. Ord, W. Inojosa, A. Matondo, D. A. Diallo, W. Mbacham, d. B. Van, T. D. Swarthout, A. Getachew, S. Dejene, M. P. Grobusch, F. Njie, S. Dunyo, M. Kweku, S. Owusu-Agyei, D. Chandramohan, M. Bonnet, J.-P. Guthmann, S. Clarke, K. I. Barnes, E. Streat, S. T. Katokele, P. Uusiku, C. O. Agboghoroma, O. Y. Elegba, B. Cissé, I. A-Elbasit, H. A. Giha, S. P. Kachur, C. Lynch, J. B. Rwakimari, P. Chanda, M. Hawela, B. Sharp, I. Naidoo, and C. Roper. Multiple Origins and Regional Dispersal of Resistant dhps in African Plasmodium falciparum Malaria. *PLoS Med*, 6(4):e1000055, 2009.

[196] S. Pelleau, E. L. Moss, S. K. Dhingra, B. Volney, J. Casteras, and S. J. Gabryszewski. Adaptive evolution of malaria parasites in French Guiana : Reversal of chloroquine resistance by acquisition of a mutation in pfcrt. *Proceedings of the National Academy of Sciences*, 112(37), 2015.

[197] P. Perlmann and M. Troye-blomberg. Malaria and the Immune System in Humans. *Malaria Immunity in Humans*, 80:229–235, 2002.

[198] I. Petersen, R. Eastman, and M. Lanzer. Drug-resistant malaria: molecular mechanisms and implications for public health. *FEBS letters*, 585(11):1551–62, 2011.

[199] D. S. Peterson, D. Walliker, and T. E. Wellems. Evidence that a point mutation in dihydrofolate reductase-thymidylate synthase confers resistance to pyrimethamine in falciparum malaria. *Proceedings of the National Academy of Sciences of the United States of America*, 85(23):9114–8, 1988.

[200] D. S. Peterson, W. K. Milhous, and T. E. Wellems. Molecular basis of differential resistance to cycloguanil and pyrimethamine in Plasmodium falciparum malaria. *Proceedings of the National Academy of Sciences of the United States of America*, 87(8):3018–22, 1990.

[201] A. P. Phyo, S. Nkhoma, K. Stepniewska, E. A. Ashley, S. Nair, R. McGready, C. ler Moo, S. Al-Saai, A. M. Dondorp, K. M. Lwin, P. Singhasivanon, N. P. Day, N. J. White, T. J. C. Anderson, and F. Nosten. Emergence of artemisinin-resistant malaria on the western border of Thailand: a longitudinal study. *The Lancet*, 379(9830):1960–1966, 2012.

[202] S. Picot, P. Olliaro, F. de Monbrison, A.-L. Bienvenu, R. N. Price, and P. Ringwald. A systematic review and meta-analysis of evidence for correlation between molecular markers of parasite resistance and treatment outcome in falciparum malaria. *Malaria Journal*, 8:89, 2009.

[203] C. V. Plowe, A. Djimde, M. Bouare, O. Doumbo, and T. E. Wellems. Pyrimethamine and proguanil resistance-conferring mutations in Plasmodium falciparum dihydrofolate reductase: polymerase chain reaction methods for surveillance in Africa. *The American journal of tropical medicine and hygiene*, 52(6):565–8, 1995.

[204] C. V. Plowe, J. F. Cortese, A. Djimde, O. C. Nwanyanwu, W. M. Watkins, P. A. Winstanley, J. G. Estrada-Franco, R. E. Mollinedo, J. C. Avila, J. L. Cespedes, D. Carter, and O. K. Doumbo. Mutations in Plasmodium falciparum dihydrofolate reductase and dihydropteroate synthase and epidemiologic patterns of pyrimethamine-sulfadoxine use and resistance. *The Journal of infectious diseases*, 176:1590–6, 1997.

[205] C. V. Plowe, J. G. Kublin, and O. K. Doumbo. P. falciparum dihydrofolate reductase and dihydropteroate synthase mutations: epidemiology and role in clinical resistance to antifolates. *Drug Resistance Updates*, 1(6):389–396, 1998.

[206] C. V. Plowe. Monitoring antimalarial drug resistance: making the most of the tools at hand. *Journal of Experimental Biology*, 206(21):3745–3752, 2003.

[207] M. Plummer, N. Best, K. Cowles, and K. Vines. CODA: convergence diagnosis and output analysis for MCMC. *R News*, 6(1):7–11, 2006.

[208] T. Ponnudurai, A. H. W. Lensen, G. J. A. van Gemert, M. G. Bolmer, and J. H. E. Th. Meuwissen. Feeding behaviour and sporozoite ejection by infected Anopheles stephensi. *Transactions of the Royal Society of Tropical Medicine and Hygiene*, 85(2):175–180, 1991.

[209] S. Portugal, H. Drakesmith, and M. M. Mota. Superinfection in malaria: Plasmodium shows its iron will. *EMBO reports*, 12(12):1233–1242, 2011.

[210] R Core Team. R: A language and environment for statistical computing. *https://www.R-project.org/*, 2013.

[211] A. Rhoads and K. F. Au. PacBio Sequencing and Its Applications. *Genomics, Proteomics and Bioinformatics*, 13(5):278–289, 2015.

[212] C. P. Robert. *The Bayesian Choice*. Springer, second edition, 2007.

[213] G. O. Roberts and S. K. Sahu. Updating Schemes, Correlation Structure, Blocking and Parameterization for the Gibbs Sampler. *Journal of the Royal Statistical Society. Series B (Methodological)*, 59(2):291–317, 1997.

[214] G. O. Roberts and R. L. Tweedie. Exponential convergence of Langevin distributions and their discrete approximations. *Bernoulli*, 2(4):341–363, 1996.

[215] S. J. Rogerson, L. Hviid, P. E. Duffy, R. F. Leke, and D. W. Taylor. Malaria in pregnancy: pathogenesis and immunity. *Lancet Infectious Diseases*, 7(2):105–117, 2007.

[216] C. Roper, R. Pearce, S. Nair, B. Sharp, F. Nosten, and T. J. C. Anderson. Intercontinental spread of pyrimethamine-resistant malaria. *Science*, 305(5687):1124, 2004.

[217] C. Roper, M. Alifrangis, F. Ariey, A. Talisuna, D. Menard, O. Mercereau-Puijalon, and P. Ringwald. Molecular surveillance for artemisinin resistance in Africa. *The Lancet. Infectious diseases*, 14(8):668–70, 2014.

[218] R. Rosenberg, R. A. Wirtz, I. Schneider, and R. Burge. An estimation of the number of malaria sporozoites ejected by a feeding mosquito. *Transactions of the Royal Society of Tropical Medicine and Hygiene*, 84(2):209–212, 1990.

[219] A. Ross, C. Koepfli, X. Li, S. Schoepflin, P. Siba, I. Mueller, I. Felger, and T. Smith. Estimating the numbers of malaria infections in blood samples using high-resolution genotyping data. *PloS one*, 7(8):e42496, 2012.

[220] J. M. Rubio, A. Benito, J. Roche, P. J. Berzosa, M. L. García, M. Micó, M. Edú, J. Alvar, M. L. Garcia, M. Mico, and M. Edu. Semi-nested, multiplex polymerase chain reaction for detection of human malaria parasites and evidence of Plasmodium vivax infection in Equatorial Guinea. *American Journal of Tropical Medicine and Hygiene*, 60(2):183–187, 1999.

[221] W. Sama, S. Owusu-Agyei, I. Felger, P. Vounatsou, and T. Smith. An immigration-death model to estimate the duration of malaria infection when detectability of the parasite is imperfect. *Statistics in Medicine*, 24(21):3269–3288, 2005.

[222] W. Sama, S. Owusu-Agyei, I. Felger, K. Dietz, and T. Smith. Age and seasonal variation in the transition rates and detectability of Plasmodium falciparum malaria. *Parasitology*, 132(Pt 1):13–21, 2006.

[223] K. A. Schneider and A. A. Escalante. A likelihood approach to estimate the number of co-infections. *PloS one*, 9(7):e97899, 2014.

[224] A. G. Schneider, Z. Premji, I. Felger, T. Smith, S. Abdulla, H.-P. Beck, and H. Mshinda. A point mutation in codon 76 of pfcrt of P. falciparum is positively selected for by Chloroquine treatment in Tanzania. *Infection, genetics and evolution : journal of molecular epidemiology and evolutionary genetics in infectious diseases*, 1(3):183–9, 2002.

[225] S. Schoepflin, F. Valsangiacomo, E. Lin, B. Kiniboro, I. Mueller, and I. Felger. Comparison of Plasmodium falciparum allelic frequency distribution in different endemic settings by high-resolution genotyping. *Malaria Journal*, 8:250, 2009.

[226] K. F. Scmidt. Inbred parasites may spur resistance (News). *Science*, 269(22 September):1670, 1995.

[227] J. M. G. Shelton, P. Corran, P. Risley, N. Silva, C. Hubbart, A. Jeffreys, K. Rowlands, R. Craik, V. Cornelius, M. Hensmann, S. Molloy, N. Sepulveda, T. G. Clark, G. Band, G. M. Clarke, C. C. A. Spencer, A. Kerasidou, S. Campino, S. Auburn, A. Tall, A. B. Ly, O. Mercereau-Puijalon, A. Sakuntabhai, A. Djimde, B. Maiga, O. Toure, O. K. Doumbo,

A. Dolo, M. Troye-Blomberg, V. D. Mangano, F. Verra, D. Modiano, E. Bougouma, S. B. Sirima, M. Ibrahim, A. Hussain, N. Eid, A. Elzein, H. Mohammed, A. Elhassan, I. Elhassan, T. N. Williams, C. Ndila, A. Macharia, K. Marsh, A. Manjurano, H. Reyburn, M. Lemnge, D. Ishengoma, R. Carter, N. Karunaweera, D. Fernando, R. Dewasurendra, C. J. Drakeley, E. M. Riley, D. P. Kwiatkowski, K. A. Rockett, and MalariaGen Consortium. Genetic determinants of anti-malarial acquired immunity in a large multi-centre study. *Malaria Journal*, 14(333):1–18, 2015.

[228] C. H. Sibley, J. E. Hyde, P. F. Sims, C. V. Plowe, J. G. Kublin, E. K. Mberu, A. F. Cowman, P. A. Winstanley, W. M. Watkins, and A. M. Nzila. Pyrimethamine-sulfadoxine resistance in Plasmodium falciparum: what next? *Trends in parasitology*, 17(12):582–8, 2001.

[229] T. Smith and P. Vounatsou. Estimation of infection and recovery rates for highly polymorphic parasites when detectability is imperfect, using hidden Markov models. *Statistics in medicine*, 22(10):1709–24, 2003.

[230] T. Smith, H. P. Beck, A. Kitua, S. Mwankusye, I. Felger, N. Fraser-Hurt, A. Irion, P. Alonso, T. Teuscher, and M. Tanner. Age dependence of the multiplicity of Plasmodium falciparum infections and of other malariological indices in an area of high endemicity. *Transactions of the Royal Society of Tropical Medicine and Hygiene*, 93(suppl 1):15–20, 1999.

[231] R. W. Snow, J. F. Trape, and K. Marsh. The past, present and future of childhood malaria mortality in Africa. *Trends in parasitology*, 17(12):593–597, 2001.

[232] A. F. Somé, Y. Y. Séré, C. Dokomajilar, I. Zongo, N. Rouamba, B. Greenhouse, J. B. Ouédraogo, and P. J. Rosenthal. Selection of known plasmodium falciparum resistance-mediating polymorphisms by artemether-lumefantrine and amodiaquine-sulfadoxine-pyrimethamine but not dihydroartemisinin-piperaquine in Burkina Faso. *Antimicrobial Agents and Chemotherapy*, 54(5):1949–1954, 2010.

[233] D. J. Spiegelhalter, N. G. Best, B. P. Carlin, and A. Van Der Linde. Bayesian measures of model complexity and fit. *Journal of the Royal Statistical Society: Series B (Statistical Methodology)*, 64(4):583–639, 2002.

[234] S. G. Staedke, A. Mpimbaza, M. R. Kamya, B. K. Nzarubara, G. Dorsey, and P. J. Rosenthal. Combination treatments for uncomplicated falciparum malaria in Kampala, Uganda: randomised clinical trial. *The Lancet*, 364:1950–1957, 2004.

[235] Stan Development Team. Stan Modeling Language: User's Guide and Reference Manual. *http://mc-stan.org/*, Stan Versi, 2015.

[236] J. Straimer, N. F. Gnädig, B. Witkowski, C. Amaratunga, V. Duru, A. P. Ramadani, M. Dacheux, N. Khim, L. Zhang, S. Lam, P. D. Gregory, F. D. Urnov, O. Mercereau-Puijalon, F. Benoit-Vical, R. M. Fairhurst, D. Ménard, and D. A. Fidock. K13-propeller mutations confer artemisinin resistance in Plasmodium falciparum clinical isolates. *Science*, 2624(1985):428–431, 2015.

[237] S.-Y. Su, J. E. Asher, M.-R. Jarvelin, P. Froguel, A. I. F. Blakemore, D. J. Balding, and L. J. M. Coin. Inferring combined CNV/SNP haplotypes from genotype data. *Bioinformatics*, 26(11):1437–45, 2010.

[238] P. L. Sutton, V. Neyra, J. N. Hernandez, and O. H. Branch. Plasmodium falciparum and Plasmodium vivax infections in the Peruvian Amazon: Propagation of complex, multiple allele-type infections without super-infection. *American Journal of Tropical Medicine and Hygiene*, 81(6):950–960, 2009.

[239] P. Tabernero, M. Mayxay, M. J. Culzoni, P. Dwivedi, I. Swamidoss, E. L. Allan, M. Khanthavong, C. Phonlavong, C. Vilayhong, S. Yeuchaixiong, C. Sichanh, S. Sengaloundeth, H. Kaur, F. M. Fernandez, M. D. Green, and P. N. Newton. A Repeat Random Survey of the Prevalence of Falsified and Substandard Antimalarials in the Lao PDR: A Change for the Better. *American Journal of Tropical Medicine and Hygiene*, 92(suppl 6):95–104, 2015.

[240] S. L. Takala, D. L. Smith, O. C. Stine, D. Coulibaly, M. A. Thera, O. K. Doumbo, and C. V. Plowe. A high-throughput method for quantifying alleles and haplotypes of the malaria vaccine candidate Plasmodium falciparum merozoite surface protein-1 19 kDa. *Malaria Journal*, 5(31), 2006.

[241] S. Takala-Harrison, T. G. Clark, C. G. Jacob, M. P. Cummings, O. Miotto, A. M. Dondorp, M. M. Fukuda, F. Nosten, H. Noedl, M. Imwong, D. Bethell, Y. Se, C. Lon, S. D. Tyner, D. L. Saunders, D. Socheat, F. Ariey, A. P. Phyo, P. Starzengruber, H.-P. Fuehrer, P. Swoboda, K. Stepniewska, J. Flegg, C. Arze, G. C. Cerqueira, J. C. Silva, S. M. Ricklefs, S. F. Porcella, R. M. Stephens, M. Adams, L. J. Kenefic, S. Campino, S. Auburn, B. Macinnis, D. P. Kwiatkowski, X.-Z. Su, N. J. White, P. Ringwald, and C. V. Plowe. Genetic loci associated with delayed clearance of Plasmodium falciparum following artemisinin treatment in Southeast Asia. *Proceedings of the National Academy of Sciences of the United States of America*, 110(1):240–245, 2012.

[242] S. Takala-Harrison, C. G. Jacob, C. Arze, M. P. Cummings, J. C. Silva, A. M. Dondorp, M. M. Fukuda, T. T. Hien, M. Mayxay, H. Noedl, F. Nosten, M. P. Kyaw, N. T. T. Nhien, M. Imwong, D. Bethell, Y. Se, C. Lon, S. D. Tyner, D. L. Saunders, F. Ariey, O. Mercereau-Puijalon, D. Menard, P. N. Newton, M. Khanthavong, B. Hongvanthong, P. Starzengruber, H.-P. Fuehrer, P. Swoboda, W. A. Khan, A. P. Phyo, M. M. Nyunt, M. H. Nyunt, T. S. Brown, M. Adams, C. S. Pepin, J. Bailey, J. C. Tan, M. T. Ferdig, T. G. Clark, O. Miotto, B. MacInnis, D. P. Kwiatkowski, N. J. White, P. Ringwald, and C. V. Plowe. Independent Emergence of Artemisinin Resistance Mutations Among Plasmodium falciparum in Southeast Asia. *Journal of Infectious Diseases*, 211(5):670–679, 2015.

[243] A. O. Talisuna, A. Nalunkuma-Kazibwe, N. Bakyaita, P. Langi, T. K. Mutabingwa, W. W. Watkins, E. Van Marck, U. D'Alessandro, and T. G. Egwang. Efficacy of sulphadoxine-pyrimethamine alone or combined with amodiaquine or chloroquine for the treatment of uncomplicated falciparum malaria in Ugandan children. *Tropical medicine & international health*, 9(2):222–9, 2004.

[244] A. Talisuna, S. Adibaku, G. Dorsey, M. R. Kamya, and P. J. Rosenthal. Malaria in Uganda: challenges to control on the long road to elimination. II. The path forward. *Acta tropica*, 121(3):196–201, 2012.

[245] M. A. Tanner and W. H. Wong. The Calculation of Posterior Distributions by Data Augmentation. *Journal of the American Statistical Association*, 82(398):528–540, 1987.

[246] S. M. Taylor, A. Antonia, G. Feng, V. Mwapasa, E. Chaluluka, M. Molyneux, F. O. ter Kuile, S. J. Rogerson, and S. R. Meshnick. Adaptive evolution and fixation of drug-resistant

Plasmodium falciparum genotypes in pregnancy-associated malaria: 9-year results from the QuEERPAM study. *Infection, genetics and evolution*, 12(2):282–90, 2012.

[247] S. M. Taylor, C. M. Parobek, N. Aragam, B. E. Ngasala, A. Mårtensson, S. R. Meshnick, and J. J. Juliano. Pooled deep sequencing of Plasmodium falciparum isolates: an efficient and scalable tool to quantify prevailing malaria drug-resistance genotypes. *The Journal of infectious diseases*, 208(12):1998–2006, 2013.

[248] A. R. Taylor, J. A. Flegg, S. L. Nsobya, A. Yeka, M. R. Kamya, P. J. Rosenthal, G. Dorsey, C. H. Sibley, P. J. Guerin, and C. C. Holmes. Estimation of malaria haplotype and genotype frequencies: a statistical approach to overcome the challenge associated with multiclonal infections. *Malaria Journal*, 13(1):102, 2014.

[249] A. Taylor Bright and E. A. Winzeler. Resistance mapping in malaria. *Nature*, 498:446–447, 2013.

[250] The Four Artemisinin-Based Combinations Study group. A head-to-head comparison of four artemisinin-based combinations for treating uncomplicated malaria in african children: A randomized trial. *PLoS Medicine*, 8(11), 2011.

[251] The Roll Back Malaria (RBM) Partnership. Executive Summary: The Global Malaria Action Plan. Technical report, 2008.

[252] The RTSS Clinical Trials Partnership. A Phase 3 Trial of RTS,S/AS01 Malaria Vaccine in African Infants. *New England Journal of Medicine*, 367(24):2284–2295, 2012.

[253] T. T. Thomsen, L. B. Madsen, H. H. Hansson, E. V. E. Tomás, D. Charlwood, I. C. Bygbjerg, and M. Alifrangis. Rapid Selection of Plasmodium falciparum Chloroquine Resistance Transporter Gene and Multidrug Resistance Gene-1 Haplotypes Associated with Past Chloroquine and Present Artemether-Lumefantrine Use in Inhambane District, Southern Mozambique. *The American journal of tropical medicine and hygiene*, 88(3):536–41, 2013.

[254] J. F. Trape, G. Pison, M. P. Preziosi, C. Enel, A. Desgrées du Loû, V. Delaunay, B. Samb, E. Lagarde, J. F. Molez, and F. Simondon. Impact of chloroquine resistance on malaria mortality. *Medical Sciences*, 321(8):689–97, 1998.

[255] T. Triglia, P. Wang, P. F. Sims, J. E. Hyde, and A. F. Cowman. Allelic exchange at the endogenous genomic locus in Plasmodium falciparum proves the role of dihydropteroate synthase in sulfadoxine-resistant malaria. *The EMBO journal*, 17(14):3807–15, 1998.

[256] Y. Tsumori, M. Ndounga, T. Sunahara, N. Hayashida, M. Inoue, S. Nakazawa, P. Casimiro, R. Isozumi, H. Uemura, K. Tanabe, O. Kaneko, and R. Culleton. Plasmodium falciparum: Differential Selection of Drug Resistance Alleles in Contiguous Urban and Peri-Urban Areas of Brazzaville, Republic of Congo. *PloS one*, 6(8):e23430, 2011.

[257] K. M. Tun, M. Imwong, K. M. Lwin, A. A. Win, T. M. Hlaing, T. Hlaing, K. Lin, M. P. Kyaw, K. Plewes, M. A. Faiz, M. Dhorda, P. Y. Cheah, S. Pukrittayakamee, E. A. Ashley, T. J. C. Anderson, S. Nair, M. McDew-White, J. A. Flegg, E. P. M. Grist, P. Guerin, R. J. Maude, F. Smithuis, A. M. Dondorp, N. P. J. Day, F. Nosten, N. J. White, and C. J. Woodrow. Spread of artemisinin-resistant Plasmodium falciparum in Myanmar: a cross-sectional survey of the K13 molecular marker. *The Lancet Infectious Diseases*, 15(4):415–421, 2015.

[258] L. S. Tusting, T. Bousema, D. L. Smith, and C. Drakeley. Measuring Changes in Plasmodium falciparum Transmission: Precision, Accuracy and Costs of Metrics. In *Advances in parasitology*, volume 84, pages 151–208. Elsevier Ltd., first edition, 2014.

[259] United Nations General Assembly. United Nations Millennium Declaration. Technical report, 2000.

[260] M. Vafa, M. Troye-Blomberg, J. Anchang, A. Garcia, and F. Migot-Nabias. Multiplicity of Plasmodium falciparum infection in asymptomatic children in Senegal: relation to transmission, age and erythrocyte variants. *Malaria Journal*, 7:17, 2008.

[261] J. C. Venter, K. Remington, J. F. Heidelberg, A. L. Halpern, D. Rusch, J. A. Eisen, D. Wu, I. Paulsen, K. E. Nelson, W. Nelson, D. E. Fouts, S. Levy, A. H. Knap, M. W. Lomas, K. Nealson, O. White, J. Peterson, J. Hoffman, R. Parsons, H. Baden-Tillson, C. Pfannkoch, Y.-H. Rogers, and H. O. Smith. Environmental genome shotgun sequencing of the Sargasso Sea. *Science*, 304(5667):66–74, 2004.

[262] S. K. Volkman, P. C. Sabeti, D. Decaprio, D. E. Neafsey, S. F. Schaffner, D. A. Milner, J. P. Daily, O. Sarr, D. Ndiaye, O. Ndir, S. Mboup, M. T. Duraisingh, A. Lukens, A. Derr, N. Stange-thomann, S. Waggoner, R. Onofrio, L. Ziaugra, E. Mauceli, S. Gnerre, D. B. Jaffe, J. Zainoun, R. C. Wiegand, B. W. Birren, D. L. Hartl, J. E. Galagan, E. S. Lander, and D. F. Wirth. A genome-wide map of diversity in Plasmodium falciparum. *Nature genetics*, 39(1):113–119, 2007.

[263] L. Von Seidlein and B. M. Greenwood. Mass administrations of antimalarial drugs. *Trends in Parasitology*, 19(10):452–460, 2003.

[264] P. Wang, C. S. Lee, R. Bayoumi, A. Djimde, O. Doumbo, G. Swedberg, L. D. Dao, H. Mshinda, M. Tanner, W. M. Watkins, P. F. Sims, and J. E. Hyde. Resistance to antifolates in Plasmodium falciparum monitored by sequence analysis of dihydropteroate synthetase and dihydrofolate reductase alleles in a large number of field samples of diverse origins. *Molecular and biochemical parasitology*, 89(2):161–77, 1997.

[265] Z. Wang, S. Shrestha, X. Li, J. Miao, L. Yuan, M. Cabrera, C. Grube, Z. Yang, and L. Cui. Prevalence of K13-propeller polymorphisms in Plasmodium falciparum from China-Myanmar border in 2007-2012. *Malaria Journal*, 14(1):168, 2015.

[266] H. Wanzira, A. Kakuru, E. Arinaitwe, V. Bigira, M. K. Muhindo, M. Conrad, P. J. Rosenthal, M. R. Kamya, J. W. Tappero, and G. Dorsey. Longitudinal Outcomes in a Cohort of Ugandan Children Randomized to Artemether-lumefantrine Verus Dihydroartemisinin-piperaquine for the Treatment of Malaria. *Clinical infectious diseases*, 59(4):509–516, 2014.

[267] D. A. Warrell and H. M. Gilles. *Essential malariology*, volume 1. ARNOLD, London, fourth edition, 2002.

[268] T. E. Wellems and C. V. Plowe. Chloroquine-resistant malaria. *The Journal of infectious diseases*, 184(6):770–6, 2001.

[269] K. A. Wetterstrand. DNA Sequencing Costs: Data from the NHGRI Genome Sequencing Program (GSP). *http://www.genome.gov/sequencingcosts/*, 2016.

[270] N. J. White, F. Nosten, S. Looareesuwan, W. M. Watkins, K. Marsh, R. W. Snow, G. Kokwaro, J. Ouma, T. T. Hien, M. E. Molyneux, T. E. Taylor, C. I. Newbold, T. K. Ruebush, M. Danis, B. M. Greenwood, R. M. Anderson, and P. Olliaro. Averting a malaria disaster. *Lancet*, 353:1965–1967, 1999.

[271] M. T. White, R. Verity, J. T. Griffin, K. P. Asante, S. Owusu-Agyei, B. Greenwood, C. Drakeley, S. Gesase, J. Lusingu, D. Ansong, S. Adjei, T. Agbenyega, B. Ogutu, L. Otieno, W. Otieno, S. T. Agnandji, B. Lell, P. Kremsner, I. Hoffman, F. Martinson, P. Kamthunzu, H. Tinto, I. Valea, H. Sorgho, M. Oneko, K. Otieno, M. J. Hamel, N. Salim, A. Mtoro, S. Abdulla, P. Aide, J. Sacarlal, J. J. Aponte, P. Njuguna, K. Marsh, P. Bejon, E. M. Riley, and A. C. Ghani. Immunogenicity of the RTS,S/AS01 malaria vaccine and implications for duration of vaccine efficacy: secondary analysis of data from a phase 3 randomised controlled trial. *The Lancet Infectious Diseases*, 3099(15):1–9, 2015.

[272] N. J. White. Assessment of the pharmacodynamic properties of antimalarial drugs in vivo. *Antimicrobial Agents and Chemotherapy*, 41(7):1413–1422, 1997.

[273] N. J. White. Antimalarial drug resistance. *Trends in Parasitology*, 113(8):1084–1092, 2004.

[274] N. J. White. Antimalarial drug resistance. *Trends in Parasitology*, 113(8):1084–1092, 2004.

[275] N. J. White. Qinghaosu (Artemisinin): The Price of Success. *Science*, 320:330–334, 2008.

[276] L. Wigger, J. E. Vogt, and V. Roth. Malaria haplotype frequency estimation. *Statistics in medicine*, 32(21):3737–3751, 2013.

[277] B. G. Williams and C. Dye. Maximum likelihood for parasitologists. *Parasitology Today*, 10(12):489–93, 1994.

[278] C. M. Wilson, S. K. Volkman, S. Thaithong, R. K. Martin, D. E. Kyle, W. K. Milhous, and D. F. Wirth. Amplification of pfmdr1 associated with mefloquine and halofantrine resistance in Plasmodium falciparum from Thailand. *Molecular and Biochemical Parasitology*, 57(1):151–160, 1993.

[279] C. Wongsrichanalai and S. R. Meshnick. Declining artesunate-mefloquine efficacy against falciparum malaria on the Cambodia-Thailand border. *Emerging infectious diseases*, 14(5):716–9, 2008.

[280] J. C. Wootton, X. Feng, M. T. Ferdig, R. A. Cooper, J. Mu, D. I. Baruch, A. J. Magill, and X.-Z. Su. Genetic diversity and chloroquine selective sweeps in Plasmodium falciparum. *Nature*, 418(6895):320–323, 2002.

[281] World Health Organization. Antimalarial drug combination therapy: report of a WHO technical consultation. Technical report, Geneva, 2001.

[282] World Health Organization. Recommended Genotyping Procedures (RGPs) to identify parasite populations. Technical report, Amsterdam, 2007.

[283] World Health Organization. Global plan for artemisinin resistance containment. Technical report, Geneva, 2011.

[284] World Health Organization. Emergency Response to Artemisinin Resistance in the Greater Mekong Subregion. Technical report, Geneva, 2013.

[285] World Health Organization. Severe malaria. *Tropical Medicine and International Health*, 19(suppl 1):7–131, 2014.

[286] World Health Organization. Global technical strategy for malaria 2016-2030. Technical report, Geneva, 2015.

[287] World Health Organization. Guidelines for the treatment of malaria: third edition. Technical report, World Health Organization, Geneva, 2015.

[288] World Health Organization. Status report on artemisinin and ACT resistance. Technical report, Geneva, 2015.

[289] World Health Organization. Strategy for Malaria Elimination in the Greater Mekong Subregion (2015-2030). Technical report, Geneva, 2015.

[290] World Health Organization. World Malaria Report 2015. Technical report, Geneva, 2015.

[291] World Health Organization. Action and investment to defeat malaria 2016-2030: for a malaria-free World. Technical report, Geneva, 2016.

[292] World Health Organization. Weekly epidemiological record: relevé épidémiologique hebdomadaire. *World Health Organization*, 91(4):33–52, 2016.

[293] WWARN. Molecular Testing for Malaria Standard Operating Procedure (SOP) DNA Extraction by Chelex.

[294] WWARN. PCR-RFLP for genotyping candidate P. falciparum artemisinin Procedure Molecular Module. *http://www.wwarn.org/tools-resources/procedures*.

[295] WWARN Artemisinin based Combination Therapy (ACT) Africa Baseline Study Group. Clinical determinants of early parasitological response to ACTs in African patients with uncomplicated falciparum malaria: a literature review and meta-analysis of individual patient data. *BMC Medicine*, 13(212), 2015.

[296] WWARN. Molecular surveyor dhfr & dhps. *http://www.wwarn.org/tracking-resistance/molecular-surveyor-dhfr-dhps*, 2016.

[297] WWARN. Molecular surveyor K13. *http://www.wwarn.org/molecular-surveyor-k13*, 2016.

[298] WWARN. Molecular surveyor pfmdr1 & pfcrt. *http://www.wwarn.org/tracking-resistance/molecular-surveyor-pfmdr1-pfcrt*, 2016.

[299] WWARN. Molecular Testing for Malaria Standard Operating Procedure (SOP): collection of blood on filterpaper. *http://www.wwarn.org/tools-resources/procedures*, 2016.

[300] WWARN. Molecular Testing for Malaria Standard Operating Procedure (SOP) DNA Extraction by QIAamp DNA Mini Kit. *http://www.wwarn.org/tools-resources/procedures*, 2016.

[301] A. Yeka, K. Banek, N. Bakyaita, S. G. Staedke, M. R. Kamya, A. Talisuna, F. Kironde, S. L. Nsobya, A. Kilian, M. Slater, and Others. Artemisinin versus nonartemisinin combination therapy for uncomplicated malaria: randomized clinical trials from four sites in Uganda. *PLoS medicine*, 2(7):e190–e190, 2005.

[302] A. Yeka, A. Gasasira, A. Mpimbaza, J. Achan, J. Nankabirwa, S. Nsobya, S. G. Staedke, M. J. Donnelly, F. Wabwire-Mangen, A. Talisuna, G. Dorsey, M. R. Kamya, and P. J. Rosenthal. Malaria in Uganda: challenges to control on the long road to elimination: I. Epidemiology and current control efforts. *Acta tropica*, 121(3):184–95, 2012.

[303] A. Yeka, R. Kigozi, M. D. Conrad, M. Lugemwa, P. Okui, C. Katureebe, K. Belay, B. K. Kapella, M. A. Chang, M. R. Kamya, S. G. Staedke, G. Dorsey, and P. J. Rosenthal. Artesunate/amodiaquine versus artemether/lumefantrine for the treatment of uncomplicated malaria in Uganda: a randomized trial. *Journal of Infectious Diseases*, pages 1–9, 2015.

[304] S. Yeung, W. Van Damme, D. Socheat, N. J. White, and A. Mills. Access to artemisinin combination therapy for malaria in remote areas of Cambodia. *Malaria Journal*, 7(96), 2008.

[305] S. Yeung, H. L. S. Lawford, P. Tabernero, C. Nguon, A. van Wyk, N. Malik, M. DeSousa, O. Rada, M. Boravann, P. Dwivedi, D. M. Hostetler, I. Swamidoss, M. D. Green, F. M. Fernandez, and H. Kaur. Quality of Antimalarials at the Epicenter of Antimalarial Drug Resistance: Results from an Overt and Mystery Client Survey in Cambodia. *The American journal of tropical medicine and hygiene*, 92(suppl 6):39–50, 2015.

[306] O. Zagordi, L. Geyrhofer, V. Roth, and N. Beerenwinkel. Deep sequencing of a genetically heterogeneous sample: local haplotype reconstruction and read error correction. *Journal of computational biology*, 17(3):417–28, 2010.
